# Natural Language Processing Technologies for Public Health in Africa: Scoping Review

**DOI:** 10.2196/68720

**Published:** 2025-03-05

**Authors:** Songbo Hu, Abigail Oppong, Ebele Mogo, Charlotte Collins, Giulia Occhini, Anna Barford, Anna Korhonen

**Affiliations:** 1 Language Technology Lab University of Cambridge Cambridge United Kingdom; 2 Cambridge Centre for Human Inspired Artificial Intelligence University of Cambridge Cambridge United Kingdom

**Keywords:** public health, global health, health promotion, essential public health functions, Africa, natural language processing, artificial intelligence, machine learning, technology, mobile phone

## Abstract

**Background:**

Natural language processing (NLP) has the potential to promote public health. However, applying these technologies in African health systems faces challenges, including limited digital and computational resources to support the continent’s diverse languages and needs.

**Objective:**

This scoping review maps the evidence on NLP technologies for public health in Africa, addressing the following research questions: (1) What public health needs are being addressed by NLP technologies in Africa, and what unmet needs remain? (2) What factors influence the availability of public health NLP technologies across African countries and languages? (3) What stages of deployment have these technologies reached, and to what extent have they been integrated into health systems? (4) What measurable impact has these technologies had on public health outcomes, where such data are available? (5) What recommendations have been proposed to enhance the quality, cost, and accessibility of health-related NLP technologies in Africa?

**Methods:**

This scoping review includes academic studies published between January 1, 2013, and October 3, 2024. A systematic search was conducted across databases, including MEDLINE via PubMed, ACL Anthology, Scopus, IEEE Xplore, and ACM Digital Library, supplemented by gray literature searches. Data were extracted and the NLP technology functions were mapped to the World Health Organization’s list of essential public health functions and the United Nations’ sustainable development goals (SDGs). The extracted data were analyzed to identify trends, gaps, and areas for future research. This scoping review follows the PRISMA-ScR (Preferred Reporting Items for Systematic Reviews and Meta-Analyses Extension for Scoping Reviews) reporting guidelines, and its protocol is publicly available.

**Results:**

Of 2186 citations screened, 54 studies were included. While existing NLP technologies support a subset of essential public health functions and SDGs, language coverage remains uneven, with limited support for widely spoken African languages, such as Kiswahili, Yoruba, Igbo, and Zulu, and no support for most of Africa’s >2000 languages. Most technologies are in prototyping phases, with only one fully deployed chatbot addressing vaccine hesitancy. Evidence of measurable impact is limited, with 15% (8/54) studies attempting health-related evaluations and 4% (2/54) demonstrating positive public health outcomes, including improved participants’ mood and increased vaccine intentions. Recommendations include expanding language coverage, targeting local health needs, enhancing trust, integrating solutions into health systems, and adopting participatory design approaches. The gray literature reveals industry- and nongovernmental organizations–led projects focused on deployable NLP applications. However, these projects tend to support only a few major languages and specific use cases, indicating a narrower scope than academic research.

**Conclusions:**

Despite growth in NLP research for public health, major gaps remain in deployment, linguistic inclusivity, and health outcome evaluation. Future research should prioritize cross-sectoral and needs-based approaches that engage local communities, align with African health systems, and incorporate rigorous evaluations to enhance public health outcomes.

**International Registered Report Identifier (IRRID):**

RR2-doi:10.1101/2024.07.02.24309815

## Introduction

### Public Health Needs in Africa

Most African countries face major challenges in meeting the sustainable development goal (SDG) 3 targets for good health and well-being [1-3]. Key public health challenges include high rates of infectious diseases, maternal and child health inequities, and a growing burden of noncommunicable diseases, alongside the critical need for resilient emergency response systems [4,5].

Some of these challenges stem from acute shortages in the health workforce and weak public health surveillance systems, among other weaknesses in public health systems [6,7]. For instance, Africa has only 1400 epidemiologists, despite an estimated need for 6000 [4]. These issues are further amplified by structural weaknesses in health systems and insufficient multisectoral coordination for health [5,8], which are particularly exposed by public health emergencies [9], such as the COVID-19 pandemic and the mpox outbreaks. During the COVID-19 pandemic, while several African countries were able to rapidly leverage their past experiences with outbreaks to respond to COVID-19, they also faced challenges, such as inadequate adherence to infection control, insufficient personal protective equipment, poor contact tracing, supply chain shortages, and a lack of training for key personnel [10].

To systematically strengthen public health capacities, the World Health Organization (WHO) has outlined 12 essential public health functions (EPHFs) [11]. These functions include a broad range of activities, from disease surveillance and health promotion to emergency preparedness and equitable access to health care services. However, many countries across the region, especially those with lower income levels (note that in 2024, Africa comprises upper-middle–income, lower-middle–income, and low-income countries [12]), face substantial challenges in fully implementing these functions, mainly because of limited financial, infrastructural, and health care workforce resources [5]. In resource-constrained settings, innovative technologies, such as artificial intelligence (AI) technologies, could play a crucial role in supporting the implementation of EPHFs [5,13], thereby improving public health outcomes and advancing progress toward achieving health-related SDGs.

### Natural Language Processing Technologies for Public Health

Natural language processing (NLP) is a vibrant interdisciplinary field within AI research, known by various terms in different disciplines, such as NLP in computer science, computational linguistics in linguistics, speech recognition in engineering, computational psycholinguistics in psychology, and language technologies in public discourse [14]. Despite the diversity in terminologies and research focuses within these disciplines, they share the common goal of enabling computers to interpret, understand, and generate human language [14,15]. NLP allows computers to perform a wide range of language-based tasks, including facilitating human-machine communication; improving human-to-human interactions; and processing text and speech data for practical NLP applications across different sectors, including public health.

NLP holds significant potential for advancing public health in Africa by addressing the ongoing challenges faced by many countries. By appropriately leveraging NLP, countries can improve health communication, enhance disease surveillance, support workforce training, and optimize limited resources [2,16,17], all of which are crucial for achieving SDG 3 targets. NLP technologies can be used to process and analyze large volumes of health data from diverse sources, including social media, medical records, and public health reports, to identify emerging health threats and track disease patterns in real time. This capability is especially valuable in regions with limited health workforce and surveillance infrastructure, as it enables faster, data-driven responses to public health emergencies.

In practice, NLP-driven tools have already shown promise in Africa. For instance, during the COVID-19 pandemic, WhatsApp chatbots in South Africa, Rwanda, and Senegal were used to disseminate reliable information and facilitate rapid COVID-19 testing, while a Telegram-based chatbot in Ghana was developed to combat misinformation and provide accurate data to the public [18]. Such tools can bridge communication gaps by delivering health information in local languages, empowering communities to recognize symptoms, prevent disease transmission, and respond more effectively. These innovations could play a transformative role in strengthening health systems across the continent, making them more resilient and responsive to both everyday health needs and unexpected crises.

Africa has made progress toward achieving some of its innovation and technology targets [19]. Specifically, the continent is making strides in mobile network coverage, with approximately 89% of the total African population now having access to mobile networks. Countries like Mali, Namibia, and Guinea-Bissau have achieved 100% 2G mobile network coverage [20]. This expanding network coverage creates new opportunities for cloud-based NLP applications in public health. Cloud computing, which uses remote servers to store, manage, and process data, allows African countries to access computing power that was previously unattainable [13]. This scalability is crucial for deploying NLP-based health solutions in resource-constrained settings where local infrastructure may be insufficient or absent. The synergy of cloud technology and increasing network accessibility opens the door to the expansion of NLP technologies in Africa, providing promising opportunities to improve public health outcomes across the continent.

However, a primary obstacle to the development of NLP applications is a lack of essential digital datasets for the >2000 languages spoken on the continent [21,22]. The development of modern NLP-based health applications for African language communities requires large-scale datasets to fully unlock the capabilities of deep learning models; however, there is a scarcity of digitized, in-language (ie, datasets collected in the specific languages spoken by the target user of the NLP technology), and in-domain (ie, datasets tailored to a specific use case or application, such as health education or disease surveillance, rather than general-purpose language) data. This scarcity is particularly profound in the health sector, where data tailored to specific African languages and contexts are often completely absent. This conjunction of linguistic diversity and data scarcity creates significant obstacles to developing effective NLP technologies tailored to Africa’s specific public health needs. Moreover, even when NLP technologies are developed, their successful deployment, validation, and integration into existing health systems are critical for achieving a meaningful positive impact. In resource-constrained environments, lessons learned from previous experience suggest that NLP technologies should be integrated into existing systems and institutions, rather than aiming to replace them [23]. This requires overcoming various obstacles, including the development of a nuanced understanding of local public health needs, the creation of sustainable and scalable solutions, and ensuring equitable access for all users [13,23-25].

### Research Gaps

Most previous reviews related to NLP technologies in public health have focused on global health [2,16,26] or low- and middle-income countries [13] as a whole, examined AI applications without focusing specifically on NLP [2,13,16,27], or focused on a single type of NLP application, such as chatbots [18]. Unlike other AI technologies, NLP applications are heavily influenced by the languages and cultures they are designed to serve [28,29]. Given Africa’s vast linguistic diversity and the complex spectrum of public health challenges faced by countries in the region, an Africa-focused review is critical for a more nuanced understanding of how NLP can be tailored to meet the diverse health needs across the continent. This approach aligns with pan-African initiatives, such as the African Union’s Agenda 2063 [30], which seeks to address health challenges and promote collaboration across borders. At the same time, modern NLP technologies often share similar development paradigms, meaning that advancements in one type of application can provide valuable insights and sometimes resources to others. These benefits extend beyond the experience gained during application development and include the shared use of digital resources across applications, often improving performance through NLP’s *transfer learning* techniques [31]. Therefore, a broader review of NLP technologies, compared to one focused on a specific application, provides researchers and developers with a more comprehensive set of evidence to guide future development.

To the best of our knowledge, this is the first scoping review to comprehensively examine the application of NLP technologies to public health in Africa. By mapping the current evidence, this review aims to provide insights into the key barriers and opportunities for the development and deployment of these technologies. Specifically, the review aims to answer five main research questions: (1) *Needs and availability:* What public health needs are being addressed by NLP technologies in Africa, and what unmet needs remain? (2) *Prevalence and distribution:* What factors influence the availability of public health NLP technologies across African countries and languages? (3) *Deployment and integration:* What stages of deployment have these technologies reached, and to what extent have they been integrated into health systems? (4) *Public health impact:* What measurable impact has these technologies had on public health outcomes, where such data are available? (5) *Outlook:* What recommendations have been proposed to enhance the quality, cost, and accessibility of health-related NLP technologies in Africa? By answering these questions, the review aims to provide actionable recommendations for future research and development.

## Methods

### Overview

The paradigms of developing NLP technologies have evolved significantly since the origin of NLP in the 1940s. The early rule-based systems, such as ELIZA [32], were followed by a shift toward machine learning–based methods in the 1990s. This new approach leveraged large datasets, reducing reliance on manually crafted rules. In 2013, the introduction of Word2Vec marked a major milestone for NLP [33], by representing words as vectors. This approach formed the foundation for neural language models. Subsequently, pretrained language models, such as BERT (Bidirectional Encoder Representations from Transformers) [34] and GPT-2 (Generative Pre-trained Transformer 2) [31], have become the backbone for NLP development, allowing systems to be fine-tuned and developed using datasets of thousands of examples.

Recent advances in large language models [35,36] have further simplified NLP development, allowing systems to achieve optimal performance after learning on just a handful of task-specific examples.

In the context of public health in Africa, the development of NLP technologies will likely use a mixture of paradigms, depending on the availability of task-specific datasets and computational resources. For the purposes of this scoping review, we define *NLP technologies* broadly to include *any computational systems that process natural language, either as input or output*. This inclusive definition ensures that the review captures a wide range of applications in public health across Africa. In Multimedia Appendix 1 [18,37-108], we provide examples of technologies that fall within or outside the scope of this review. The scoping review maps the current evidence on NLP technologies within the framework of EPHFs. The review follows the PRISMA-ScR (Preferred Reporting Items for Systematic Reviews and Meta-Analyses extension for Scoping Reviews) reporting guidelines [109] (see the PRISMA-ScR checklist in Multimedia Appendix 2), and its review protocol is available on *medRxiv* [110].

### Search Strategy

A systematic search of *academic literature* was conducted on May 13, 2024, and updated on October 3, 2024, using the following five electronic bibliographic databases:

1. *MEDLINE via PubMed:* Medical and public health literature
2. *ACL Anthology:* NLP and language science literature
3. *Scopus:* Broad interdisciplinary scope, including medical research
4. *IEEE Xplore:* Engineering literature, particularly in NLP and health informatics
5. *ACM Digital Library:* Computing literature, including NLP and health informatics

The search included studies published from January 1, 2013, to October 3, 2024, with the aim being to capture recent developments in NLP, particularly after the introduction of neural language models in 2013 [33]. No language restrictions were applied, although the search terms were in English.

Search terms were developed around three key areas:

1. *Africa:* The names of all 55 African Union member countries and African languages with >1 million native speakers
2. *Public health:* On the basis of the 12 EPHFs outlined by the WHO
3. *NLP:* As suggested by a team of experts in the field

These terms were combined with general phrases and Medical Subject Headings. The search strategy for each database was tailored with database-specific features to enhance the retrieval of relevant studies. The complete search strategy for MEDLINE (PubMed) and the full list of search terms are detailed in Multimedia Appendix 1. Reference chaining of relevant articles was also conducted.

Discussion of NLP technologies for public health in Africa occurs beyond the academic literature, spanning a diverse array of contributors, formats, and outlets. As such, sparse academic literature on this topic does not necessarily indicate a lack of progress [13]. Many promising technologies addressing public health challenges in Africa are introduced through media outlets, as well as by the individuals, companies, governments, and nongovernmental organizations (NGOs) that develop and use them. These contributions are often presented on the web or shared at events, such as conferences. Therefore, this scoping review also mapped evidence from a broad gray literature. In addition to structured academic databases, the following gray literature sources were included: (1) preprints, non–peer-reviewed studies, and reports; (2) media articles and blog posts; (3) commercial products from startups and established companies; (4) initiatives led by NGOs; and (5) proceedings and presentations from events and conferences. The complete search strategy for gray literature is available in Multimedia Appendix 1.

### Screening and NLP Technology Selection Criteria

This review includes NLP technologies designed to support public health in Africa. In addition, we consider digital and computational resources essential for the development and deployment of these technologies, such as digital datasets, hardware, and software toolkits.

The selection of sources for this scoping review followed a systematic 2-step screening process. Initially, titles and abstracts were reviewed by one reviewer (AO) to exclude studies meeting any of the predefined exclusion criteria (Textbox 1), such as those not involving NLP technologies, unrelated to public health, or lacking a focus on Africa. Studies that passed this initial screening were then subjected to full-text screening, where studies meeting all the inclusion criteria (Textbox 1) were included. The full-text screening was conducted by the same reviewer (AO), and the reasons for exclusion at this stage were documented.

Before the formal screening process, pilot screenings were conducted to refine our screening guidelines (Multimedia Appendix 1) and to ensure consistency and accuracy in study selection. In these pilots, 10% (179/1791 for title and abstract screening and 36/361 for full-text screening) of candidate papers were randomly selected and independently reviewed by 2 trained reviewers (AO and CC) following the predefined screening guidelines. Interreviewer agreement was assessed using exact match rate and Cohen κ to ensure reliability. The screening process and guidelines were iteratively refined by a review coordinator (SH), and the pilot screening was repeated until both an exact match rate of 0.9 and a Cohen κ score of 0.8, indicating almost perfect agreement, were achieved. Following the pilot, formal screening was conducted by a single reviewer (AO), with any concerns resolved by a review coordinator (SH), a subject matter expert in NLP.

**Inclusion and exclusion criteria.**

**Inclusion criteria**

- All types of scientific publications aimed at an academic audience (eg, peer-reviewed articles, conference proceedings, and book chapters); for gray literature search, other web-based publications (eg, blog posts and media outlets)
- Studies focusing on the development, evaluation, or adaptation of natural language processing (NLP) technologies specifically promoting public health
- Studies demonstrating direct or indirect relevance to the population in the continent of Africa
- Studies published between January 1, 2013, and October 3, 2024
- Studies published in any language

**Exclusion criteria**

- Articles without full-text availability; for articles not available through Cambridge University libraries, full text was requested by emailing the authors
- Studies unrelated to NLP technologies or their application to public health; for example, non-NLP applications, where no language technologies were involved, and the technology was used to perform tasks, such as predicting outcomes solely from structured datasets or images
- Studies focused on non-African contexts, except where such studies offer comparative insights relevant to African NLP technologies
- Studies published before January 1, 2013, or after October 3, 2024
- No language requirement specified

### Data Extraction and Synthesis

Data were extracted for each included study based on a predefined data extraction template (Multimedia Appendix 1), capturing key information on study descriptions, the characterization of NLP technologies (Textbox 2), and their contributions to EPHFs, SDGs, and SDG 3 targets specifically. In addition, where such data were available, any public health outcomes measured and recommendations for future development were documented. One reviewer (AO) completed the data extraction for all included studies, with any concerns resolved through team discussions. Due to the heterogeneity of study goals, methodologies, evaluation methods, and outcomes, a formal meta-analysis was not attempted. Instead, a narrative synthesis of the results was conducted, with introduced NLP technologies characterized according to the categories outlined in the data extraction template. The extracted data were analyzed to identify trends, gaps, and areas for future research. In addition, the authors’ recommendations for future development were documented and summarized to provide guidance for advancing NLP technologies in public health.

A similar pipelined approach of screening, data extraction, and synthesis was applied to the gray literature. Given that our gray literature search covered sources beyond academic publications, we omitted undisclosed data extraction items, as commercial products often lack full disclosure of their design and implementation. A detailed description of our approach to identifying and synthesizing the gray literature is available in Multimedia Appendix 1.

**Selected characterization of natural language processing technologies in public health.**

- Natural language processing (NLP) applications: The NLP application each system performs, such as conversational assistant, language translation, or automated diagnosis
- Modality: Type of data processed by the NLP application (eg, text, audio, and image)
- Supported languages: The set of languages supported by the NLP technology; languages are documented using ISO (International Organization for Standardization) 639-2 codes
- Target countries: Countries or regions where the introduced NLP technology is applied or intended to be used; countries are documented using the Alpha-3 code from the ISO 3166 standard
- Evaluation method (Adapted from Laranjo et al [111])
  Technical performance: Intrinsic evaluation measures such as accuracy, precision, recall, and F1 score
  User experience: Results on usability testing, user satisfaction surveys, and qualitative feedback from health care providers
  Health-related measure: Extrinsic evaluation measures such as patient engagement rates, reduction in diagnostic errors, or improvements in treatment outcomes
- Domain coverage
  General domain: Data concerning general language processing outside specialized contexts
  Research domain: Research articles and professional materials for expert audiences
  Clinical domain: Clinical notes, patient interactions, and other health care–specific communications
- Target users
  Health care providers: Direct care providers including physicians, nurses, practitioners, community health workers, and other health care professionals
  Public health officials and policy makers: Individuals involved in public health policy, administration, and epidemiology
  Researchers and data scientists: Academics and professionals focused on public health research and data analysis
  Specific equity-seeking groups: Populations grouped by protected demographic characteristics, such as people with disabilities, children, LGBTQ+ (lesbian, gay, bisexual, trans, queer) individuals, and older adults, who advocate for health equity within and beyond their group
  General public: The broader community, especially those at higher risk or in need of specific health interventions
  Others: Any target users that do not fit into the above categories
- Deployment stage
  Conceptualization: This initial stage is when the need for an NLP application is identified, and its feasibility is considered
  Design and prototyping: Development of initial prototypes; these prototypes are usually evaluated based on their technical performance
  Validation: Rigorous testing of the system with public health outcomes to validate its effectiveness and efficiency in real-world settings
  Deployment and operational: Deployment of the NLP technology in actual public health settings, where it is actively used
  Not applicable: The study does not introduce or use any new NLP technologies
- Level of accessibility
  Open-source: Publicly accessible datasets and tools that are open-source for future research and analysis
  Publicly available: Datasets and NLP applications that are accessible to the general public via web or mobile but not necessarily open-source
  Limited access: Datasets and NLP applications available only to certain users or under specific conditions
  Closed access: Datasets and applications that are not openly accessible outside the group of authors but may be available upon request or through collaboration
- Available platform
  Mobile apps: Technologies accessible via mobile apps
  Web-based applications: Technologies accessible via web applications or web-based platforms
  Web service: Technologies accessible via web-based application programming interfaces without user interfaces
  Dataset: Specific datasets published in the study
  NLP tool and library: Specific NLP tools and libraries; these tools usually require installations on each deployed computer, which require expertise in computer science

## Results

### Overview

**Figure 1:**
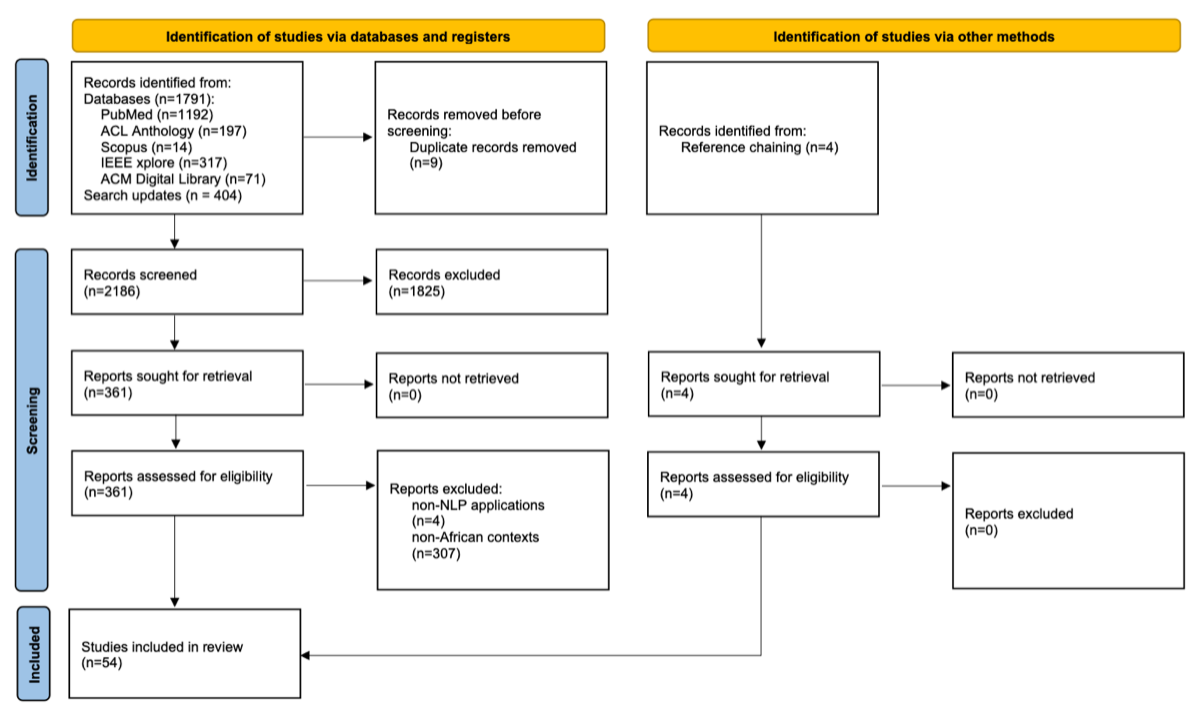
PRISMA (Preferred Reporting Items for Systematic Reviews and Meta-Analyses) flow diagram showing the search and study selection process for 54 included studies. NLP: natural language processing.

The initial database search on May 13, 2024, retrieved 1791 citations, and the final updated search on October 3, 2024, retrieved an additional 404 citations (Figure 1). The updated search retrieved 289 additional articles from PubMed, 70 from ACL Anthology, 2 from Scopus, 36 from IEEE Xplore, and 7 from ACM Digital Library, resulting in 6 additional papers being identified for full-text eligibility assessment. After removing 9 duplicate citations, 2186 unique records were screened. During the title and abstract screening, 1825 articles were excluded. Full-text reviews were conducted for the remaining 361 articles, which included 6 articles identified through the final updated search. Following the full-text screening, 311 articles were excluded, resulting in the inclusion of 2.29% (50/2186) studies. An additional 4 studies were identified through reference chaining of the included studies. Before the formal screening process, 3 rounds of pilot screenings, covering 10% (179/1791 for title and abstract screening and 36/361 for full-text screening) of the citations, were conducted to ensure consistency and reliability. The final round achieved interreviewer agreement scores of 0.97 for accuracy and 0.89 for Cohen κ in title and abstract screening, and perfect agreement (ie, 1.0 for both measures) in full-text screening.

In this section, we provide an overview of the academic literature on NLP technologies for public health in Africa and present our findings in response to the 5 aforementioned research questions. In addition, we separately summarize relevant gray literature, which provides complementary perspectives to the academic literature. By combining these 2 sources of evidence, we aim to provide a comprehensive and up-to-date analysis of the landscape, while adhering to the rigorous methodological standards of this scoping review.

### Description of Academic Literature

Over the past decade, there has been a rapid increase in the number of publications on NLP for public health in Africa, with a notable spike in 2022, where 6 (43%) out of the 14 papers published that year were in response to the COVID-19 pandemic (Textbox 3). The number of academic papers from authors affiliated with African and non-African institutions has been similar (Textbox 3). Of the 54 included citations, 38 (70%) papers were contributed by authors affiliated with African institutions [18,37-73], while 35 (65%) papers [38,40-44,46,48,50-52,59,61,62,64,66,69,71,73-89] were authored by researchers affiliated with institutions outside Africa. For readability, we do not provide a full list of in-text citations for all our categorizations throughout this review, instead highlighting specific papers where necessary. A complete table with all the categorizations and their corresponding references is available in Multimedia Appendix 1. Notably, 19 (35%) papers [38,40-44,46,48,50-52,59,61,62,64,66,69,71,73] stem from collaborations between African and non-African institutions, highlighting the prevalence and importance of cross-border and cross-continental collaborations at the intersection of NLP and public health research. Among these 19 papers, 16 (84%) involved coauthorship between researchers from North America and Africa, 6 (32%) involved coauthorship between Asia and Africa, 4 (21%) involved coauthorship between Europe and Africa, and 1 (5%) involved coauthorship between Oceania and Africa. Researchers affiliated with institutions in the United States and South Africa (18/54, 33% papers each) emerged as the leading contributors to NLP research for public health in Africa.

**Number of papers by publication years, author institutional affiliations by country, languages supported, and African countries and regions supported in the 54 included studies.**

**Publication years**

- 2015: n=1; 2016: n=2; 2017: n=2; 2018; n=1; 2019: n=5; 2020: n=9; 2021: n=8; 2022: n=14; 2023: n=9; 2024: n=3
- Note that data gathering ended on October 3, 2024, providing only 9 months of data for 2024, which means the 2024 data are not directly comparable to the other years.

**Author institutional affiliations by country**

- South Africa: n=18; the United States: n=18; Kenya: n=8; Canada: n=6; India: n=5; Germany: n=3; Iran: n=3; Rwanda: n=3; Saudi Arabia: n=3; the United Kingdom: n=3; others: n=23
- Countries with fewer than 2 papers are grouped under others, including Belgium, Brazil, Cameroon, Egypt, Eswatini, Ethiopia, France, Hungary, Indonesia, Italy, Lebanon, Lesotho, Morocco, Netherlands, New Zealand, Nigeria, Qatar, Senegal, Sierra Leone, Spain, Switzerland, Tanzania, and Uganda.
- Note that a single paper can have authors from multiple countries, so the total number of country affiliations exceeds the total number of papers reviewed.

**Language supported**

- English: n=40; Arabic: n=8; Kiswahili: n=7; French: n=4; Zulu: n=4; Amharic: n=3; Hausa: n=3; Hindi: n=3; Northern Sotho: n=3; Xhosa: n=3; others: n=64
- Languages supported by <3 technologies are grouped under others, which includes Afrikaans (n=2), Bengali (n=2), Chinese (n=2), Gujarati (n=2), Igbo (n=2), Indonesian (n=2), Japanese (n=2), Korean (n=2), Marathi (n=2), North Ndebele (n=2), Portuguese (n=2), Sinhala (n=2), Sotho (n=2), Spanish (n=2), Urdu (n=2), Assamese (n=1), Central Atlas Tamazight (n=1), Czech (n=1), Dutch (n=1), German (n=1), Iloko (n=1), Italian (n=1), Kannada (n=1), Kikuyu (n=1), Kinyarwanda (n=1), Luo (n=1), Malay (n=1), Malayalam (n=1), Nepali (n=1), Nyankole (n=1), Panjabi (n=1), Persian (n=1), Polish (n=1), Pushto (n=1), Russian (n=1), Shona (n=1), Somali (n=1), Swati (n=1), Tagalog (n=1), Tamil (n=1), Telugu (n=1), Thai (n=1), Tigrinya (n=1), Tsonga (n=1), Tswana (n=1), Turkish (n=1), Uighur (n=1), Venda (n=1), and Yoruba (n=1).
- Languages are identified using the ISO 639-2 code, and different dialectal variants of a language (eg, Arabic) are not distinguished.
- A single technology may support multiple languages.

**African countries and regions supported**

- South Africa: n=25; Kenya: n=14; Nigeria: n=9; Rwanda: n=7; Egypt: n=5; Ethiopia: n=5; Uganda: n=5; Zimbabwe: n=4; Cameroon: n=3; Eritrea: n=3; Morocco: n=3; Somalia: n=3; Tunisia: n=3; others: n=55
- Countries and regions supported by <3 technologies are grouped under others, which includes Algeria (n=2), Botswana (n=2), Democratic Republic of the Congo (n=2), Eswatini (n=2), Lesotho (n=2), Malawi (n=2), Mozambique (n=2), Namibia (n=2), Niger (n=2), Senegal (n=2), South Sudan (n=2), Sudan (n=2), Tanzania (n=2), Angola (n=1), Benin (n=1), Burkina Faso (n=1), Burundi (n=1), Cabo Verde (n=1), Central African Republic (n=1), Chad (n=1), Comoros (n=1), Congo (n=1), Côte d’Ivoire (n=1), Djibouti (n=1), Equatorial Guinea (n=1), Gabon (n=1), Gambia (n=1), Ghana (n=1), Guinea (n=1), Guinea-Bissau (n=1), Liberia (n=1), Libya (n=1), Madagascar (n=1), Mali (n=1), Mauritania (n=1), Mauritius (n=1), Sao Tome and Principe (n=1), Seychelles (n=1), Sierra Leone (n=1), Togo (n=1), Western Sahara (n=1), and Zambia (n=1).
- Countries and regions are identified using the ISO 3166 code.

Approximately 48% (26/54) of the papers included in this review did not disclose their source of funding. Of the 28 (52%) papers that did report funding, some of them received funding from more than one source, with the vast majority of papers (26/28, 93%) supported by public entities. This included government grants, NGOs, and research councils. Only 2 (4%) papers were funded by industry actors [38,88]. Geographically, with international funding sources determined by their headquarters’ location, most of the papers that disclosed funding were financially supported by institutions in North America (14/54, 26%) and Europe (9/54, 17%). Reflecting the global nature of these research contributions, funding was also sourced from other continents, including Africa and Asia, demonstrating the wide range of financial support for these studies. Notably, no funding was recorded from Oceania and South America.

The data used to develop NLP technologies for public health in Africa is generally up-to-date. Among the 36 (67%) papers that reported the year of data collection, most studies (30/54, 56%) used data collected either in the same year or within 1 year before publication. Specifically, 13 (24%) papers used data collected in the same year, while 17 (31%) used data with a 1-year delay. In this review, for papers introducing a dataset, unless otherwise specified, assume the year of data collection is the same as the year of publication. For papers that introduce NLP applications or perform an analysis using a data set from other sources, the year of data collection is the year when the data was originally published.

Most of the data (36/54, 67% papers) fall within the general domain, primarily produced and consumed by the general public, such as social media data. In addition, 15 (28%) studies covered clinical domains, including clinical notes, patient interactions, and other health care–specific communications, while 8 (15%) papers focused on the research domain, covering research articles and professional materials aimed at expert audiences. It should be noted that one study can cover multiple domains simultaneously, as seen in 5 (9%) studies. Regarding data modality, most of the data (53/54, 98% studies) used to develop these technologies were text-based, with minimal use of other modalities. Only 4 (7%) papers used audio [47,63] or image [44,48] data, highlighting a limited exploration of non–text-based data in NLP applications for public health in Africa.

### Needs and Availability

Most of the reviewed papers focused on conversational assistants (17/54, 31%) and sentiment analysis (15/54, 28%). Additional applications included machine translation (3/54, 6%), thematic analysis (3/54, 6%), information extraction (3/54, 6%), and outbreak detection (2/54, 4%). Fewer papers addressed tasks, such as infection detection, misinformation detection, disease prediction, optical character recognition, question-answering, hate speech detection, medical report generation, and speech recognition, with each of these applications covered by only 1 study. A smaller subset of studies focused on fundamental NLP challenges in the context of public health, such as syntax parsing (1/54, 2%), word embedding (1/54, 2%), and lexical processing (1/54, 2%), rather than user-facing applications.

**Figure 2:**
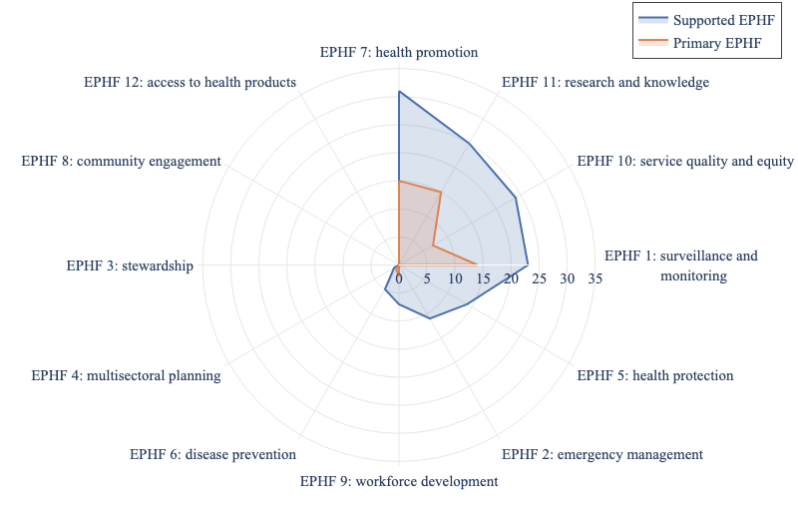
Distribution of all the essential public health functions (EPHFs) to which the 54 included papers contribute. The blue line represents EPHF coverage, where each paper may contribute to multiple EPHFs. The orange line indicates the primary EPHF, where each paper is assigned a single, most relevant EPHF focus.

Most available NLP technologies for public health in Africa were designed to serve expert users, such as researchers (45/54, 83%), policy makers (38/54, 70%), and health care providers (30/54, 56%). Fewer than half of the systems were public-facing (25/54, 46%) and targeted toward equity-seeking groups (8/54, 15%). This focus on expert-driven systems suggests an opportunity to develop more public-facing NLP technologies that engage and empower communities to proactively manage their health. After mapping the currently available NLP technologies into the WHO’s EPHF framework, Figure 2 shows that 9 (75%) out of 12 EPHFs were addressed by existing NLP technologies in Africa, with EPHF 3 (ie, public health stewardship), EPHF 8 (ie, community engagement and social participation), and EPHF 12 (ie, access to and utilization of health products, supplies, equipment, and technologies) remaining unaddressed. Most studies predominantly focused on 4 EPHFs: EPHF 7 (ie, health promotion; 31/54, 57%), EPHF 11 (ie, public health research, evaluation, and knowledge; 25/54, 46%), EPHF 10 (ie, health service quality and equity; 24/54, 44%), and EPHF 1 (ie, public health surveillance and monitoring; 23/54, 43%). Work on other EPHFs remains relatively sparse, with only a handful of papers addressing them. When each paper was assigned one primary EPHF, only 6 EPHFs were the main focus of these studies, leaving 6 EPHFs unaddressed, including EPHF 2 (ie, public health emergency management), EPHF 3, EPHF 4 (ie, multisectoral planning, financing, and management for public health), EPHF 5 (ie, health protection), EPHF 8, and EPHF 12.

**Figure 3:**
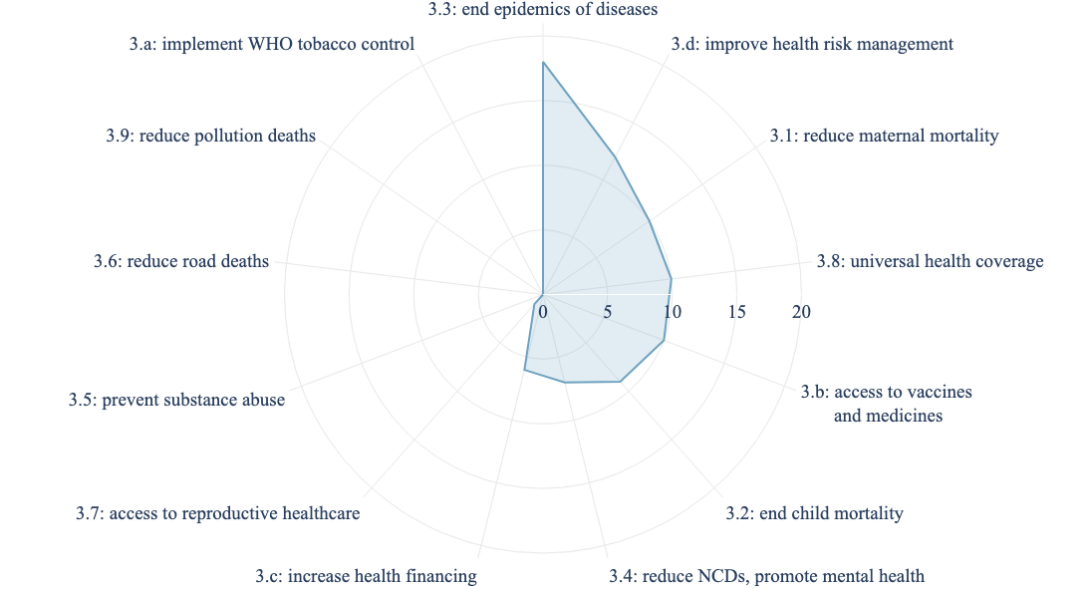
Distribution of specific targets (3.1-3.9) and means of implementation (3.a-3.d) under Sustainable Development Goal 3 (good health and well-being) to which the 54 included papers contribute. NCD: noncommunicable disease; WHO: World Health Organization.

When mapping the included NLP technologies to the United Nations’ SDGs, all the reviewed NLP technologies contributed to SDG 3 (good health and well-being). The interconnected nature of the SDGs means that contributing to one SDG often supports progress in others. For example, 15/54 (28%) studies contributed to SDG 10 (reduced inequality), 10/54 (19%) studies to SDG 9 (industry, innovation, and infrastructure), 6/54 (11%) studies to SDG 8 (decent work and economic growth), 5/54 (9%) studies to SDG 4 (quality education), 4/54 (7%) studies to SDG 5 (gender equality), 1 (2%) study to SDG 15 (life on land), and 1 (2%) study to SDG 16 (peace, justice, and strong institutions). Thus, even with a primary focus on health, these projects may have a far-reaching impact on other SDGs [112]. When zooming in on the targets of SDG 3, currently available NLP technologies in Africa only cover 6 of the 13 specific targets (Figure 3). Of the 4 means of implementation listed for SDG 3, available technologies engage 3 (ie, tobacco control, access to vaccines and medicines, and health financing).

### Prevalence and Distribution

The availability of NLP technologies for public health in Africa is strongly influenced by the languages these technologies support. Most NLP technologies predominantly serve widely spoken high-resource languages (Textbox 3), such as English (40/54, 74%), Arabic (8/54, 15%), and French (4/54, 7%), reflecting their status as official languages in academic, governmental, and professional sectors across the continent. In contrast, support for indigenous African languages is significantly limited. While some widely spoken African languages, such as Kiswahili (7/54, 13%) and Zulu (4/54, 7%), are represented, many other indigenous languages remain underrepresented or entirely absent from these technologies. Overall, 59 languages were supported by the 54 studies included in this review, a number that falls far short of covering Africa’s linguistic diversity.

The availability of NLP technologies for public health in Africa varies significantly across countries and regions. As shown in Textbox 3, South Africa is the primary target country for these technologies, with 25 (46%) out of 54 studies targeting this country, followed by Kenya (14/54, 26%) and Nigeria (9/54, 17%). In contrast, 29 African countries and regions, including Angola, Benin, and Burkina Faso, are supported by only 1 technology each, highlighting uneven distributions of NLP technologies across the continent.

**Figure 4:**
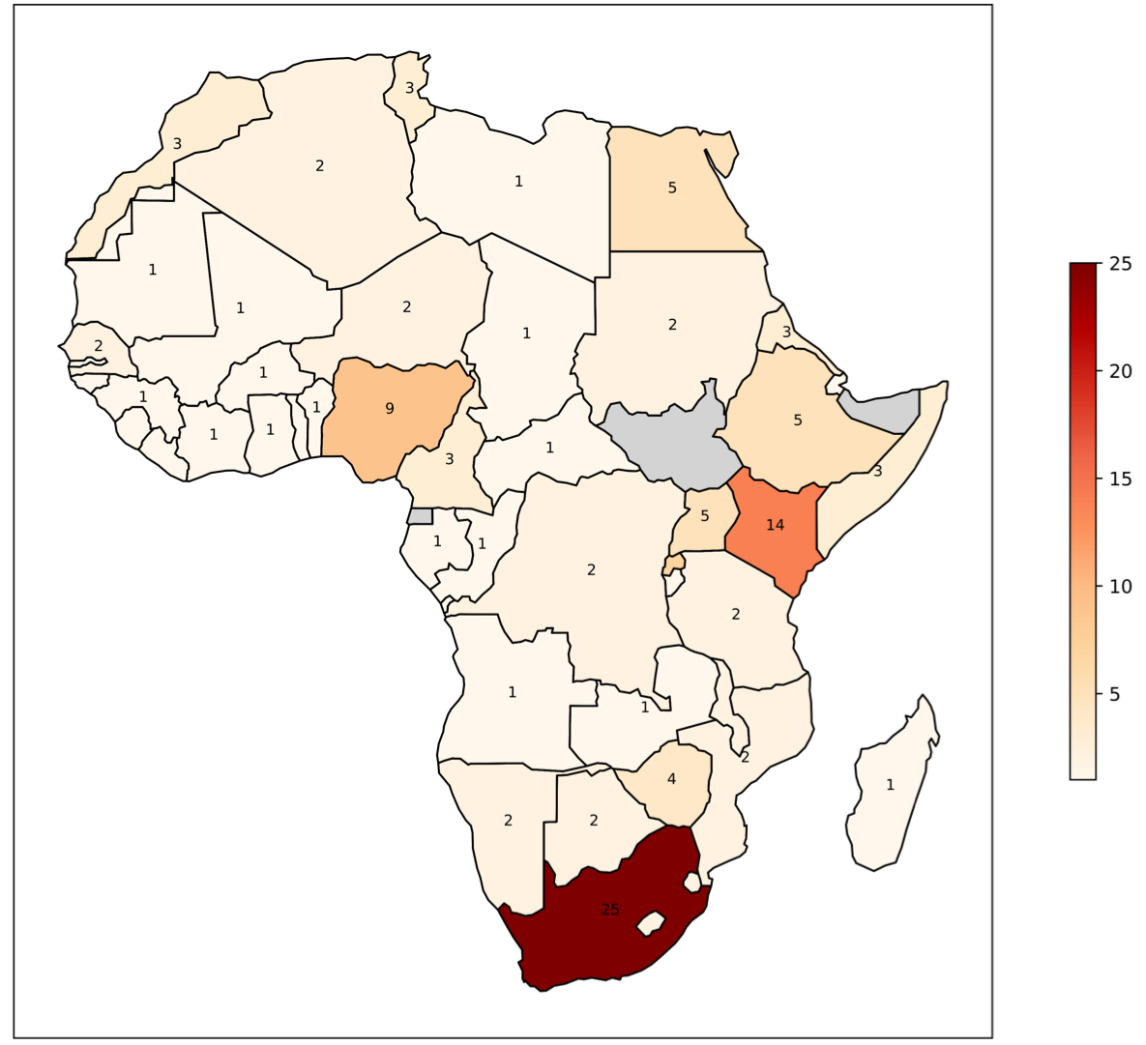
A map illustrating the distribution of target countries across Africa based on the 54 studies included in this review. Darker shades indicate a higher number of studies per country, as outlined in Textbox 3. Note that small African island states are missing from this map.

**Figure 5:**
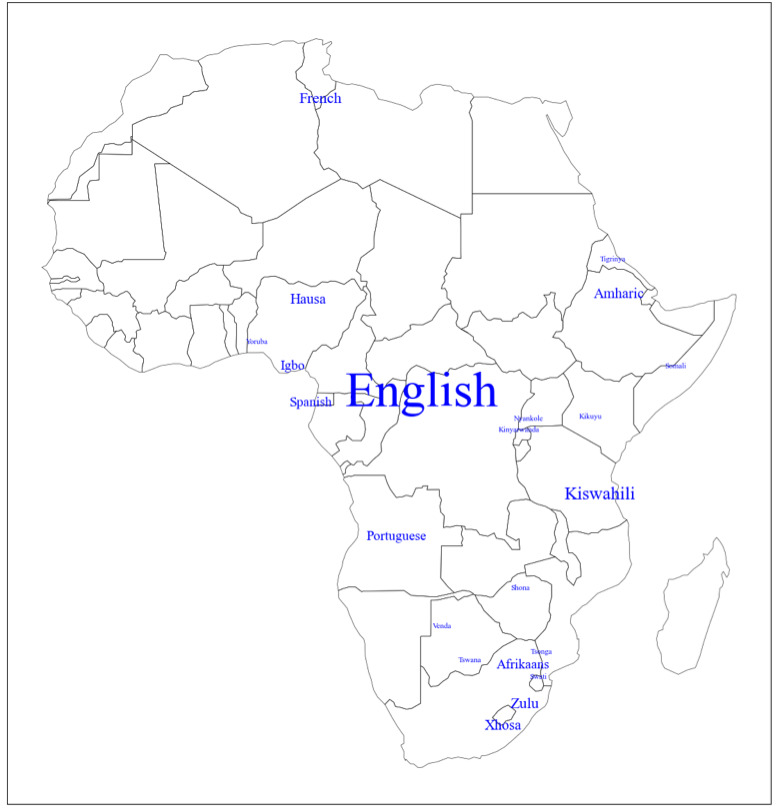
A map illustrating the distribution of supported languages across Africa based on the 54 studies included in this review. Each name is positioned near the geographic center of its primary speaking population, with a larger font size indicating a greater number of studies available. The data on supported languages is based on Textbox 3. Note that our study used English language search terms and small African island states are missing from this map.

Our review reveals a geographic concentration of available NLP technologies in certain countries, especially South Africa, Kenya, and Nigeria, suggesting a need for future efforts to expand NLP technology development to underserved regions (Figure 4). The results further highlight a major gap in linguistic inclusivity within the existing NLP technologies across the continent, where languages spoken in these better-supported regions receive more attention compared to those in other areas (Figure 5).

### Deployment and Integration

Among the 54 included studies, 4 (7%) were reviews of existing NLP technologies related to health chatbots [18], HIV prevention in Africa [84], HIV prevention in Malawi specifically [85], and chatbots for HIV prevention [59]. These reviews do not introduce new NLP technologies but rather summarize findings from other research that has introduced new technologies and reported primary results. As such, the concept of deployment is less relevant to these review studies. Therefore, this subsection focuses on the 50 studies that directly introduce new NLP applications.

Most NLP applications for public health in Africa are still in the early stages of development, with only 1 (2%) out of the 50 studies fully deployed and operational. This deployed system is a Facebook messenger chatbot designed to address vaccine hesitancy in Kenya and Nigeria, collecting real-time data on vaccine hesitancy trends from user interactions [89]. Most studies (44/50, 88%) are in the *design and prototyping* phase, where they are evaluated only based on their technical performance in controlled, lab-based environments. Meanwhile, 5 (10%) studies have reached the *validation* stage, where their effectiveness has been tested in real-world settings through methods, such as expert reviews [37,74] and user testing [39,44,45]. Specifically, 2 (4%) studies [37,74] involved health care professionals who reviewed system performance in practical scenarios. The remaining 3 (6%) studies [39,44,45] conducted evaluations with small samples of target users to test the developed NLP technologies. However, these systems were accessible only to a limited number of users and have not yet achieved full deployment.

Regarding accessibility (as defined in Textbox 2), >half (29/50, 58%) of the NLP applications are publicly accessible, allowing general use without significant restrictions. Of these, 12 (24%) are *open-source*, enabling researchers and developers to build new NLP applications based on their published systems. By contrast, a significant number of systems are categorized as having *limited access* (18/50, 36%) or are *closed access* (3), likely due to the sensitive nature of health-related data, raising concerns around privacy and data security.

In terms of platform support, most NLP technologies for public health in Africa are offered as tools and libraries (29/50, 58%), datasets (5/50, 10%), or web services (4/50, 8%), all of which require a certain level of technical expertise in computer science to exploit effectively. A smaller proportion of technologies are provided as mobile apps (11/50, 22%) and web-based applications (9/50, 18%), offering more user-friendly interfaces that can be accessed by a broader range of users, including public health practitioners and the general public. This distribution suggests an opportunity to develop NLP technologies with more accessible interfaces to promote wider adoption and usability of these technologies for public health across Africa.

Of the 50 reviewed NLP technologies, 40 (80%) indicated an intent to integrate their solutions into existing public health systems, and 41 (82%) were designed to be interoperable with various health infrastructures. This reflects a clear recognition among researchers and developers of the importance of ensuring that these technologies function seamlessly within current health frameworks. However, despite this intent, only 1 (2%) study has reached the stage of deployment, highlighting the need to move these technologies from development into operational use.

### Scope and Public Health Impact

Out of the 54 studies reviewed, 30 (56%) aimed to develop NLP applications or inform public policies to improve public health outcomes in Africa. Of these, 22 (41%) specifically focused on addressing public health challenges within African countries, directly targeting the health issues faced by local communities. The remaining 8 (15%) studies adopted a broader global health perspective. While they do not exclusively tailor their approaches to Africa, these studies aim to promote public health on a global scale, with intended outcomes that also benefit Africa. The other 24 (44%) studies were divided into 18 (33%) studies that advance NLP technologies for public health using African data (eg, social media, health records, or public health data) without directly targeting specific health challenges, and 6 (11%) studies that contribute to global health discussions, with Africa serving as a case study or example.

In terms of evaluation, nearly all studies (51/54, 94%) reported technical performance using a variety of automatic evaluation metrics. Classification metrics were the most commonly used, such as accuracy (used in 22/54, 41% studies), precision (13/54, 24%), *F*_1_-score (15/54, 28%), and recall (11/54, 20%), making these the 4 most frequently applied automatic metrics. However, because of the wide variation in evaluation approaches, direct comparisons between the studies were impractical and not attempted. In contrast, only 11 (20%) studies reported evaluation results based on user experiences, such as usability testing, user satisfaction surveys, or qualitative feedback from health care providers. Furthermore, only 8 (15%) studies attempted to evaluate these technologies using health-related measures. Among these, 2 (4%) studies confirmed a positive impact on public health outcomes, with NLP-based interventions shown to improve participants’ mood [71] and increase vaccine intentions and willingness [89].

### Outlook and Ethical Consideration

Among the 54 included papers, 20 (37%) provided recommendations for the future development of NLP technologies for public health in Africa. A thematic analysis of these recommendations identified 6 key areas for future research: addressing specific public health challenges with NLP (11/54, 20%), expanding data coverage for underrepresented languages (8/54, 15% studies), contextualizing solutions to local health needs (6/54, 11% studies), enhancing trust and ethical standards (5/54, 9% studies), integrating NLP technologies with existing health systems (5/54, 9% studies), and incorporating participatory design with domain expert input (3/54, 6% studies).

In terms of ethical considerations, 46 (85%) out of 54 studies attempted to engage stakeholders during the study design and implementation, while 38 (70%) studies explicitly addressed data privacy compliance. Approximately half of the studies (26/54, 48%) involved the local community in their research. However, only 16 (30%) studies reported receiving explicit ethics approval from an independent review board, and 10 (19%) studies mentioned obtaining informed consent from human participants. It is important to note that not all types of studies involved human participants or required explicit ethics approval in advance. In addition to these considerations, 45 (83%) papers highlighted other ethical concerns, including bias and fairness (11/54, 20%), cultural relevance and appropriateness (11/54, 20%), avoiding miscommunication by NLP technologies (9/54, 17%), preventing misuse of NLP technologies (9/54, 17%), data sharing and accessibility (7/54, 13%), adherence to regulatory standards (2/54, 4%), data representativeness (1/54, 2%), and fair compensation for participants (1/54, 2%).

### Description of Gray Literature

Our gray literature review covered two types of sources: (1) academic literature, including unpublished preprints and peer-reviewed articles not indexed in the 5 structured databases, and (2) nonacademic sources, such as online articles, blog posts, products from startups and established companies, initiatives from NGOs, and proceedings from events and conferences. Full results of the gray literature search are detailed in Multimedia Appendix 1, with key findings highlighted below.

Within the academic gray literature, we identified 11 relevant articles from the first 100 Google Scholar results, with 9 (9%) peer-reviewed articles and 2 (2%) preprints. These studies generally aligned with the patterns observed in the aforementioned structured database search. Each study involved researchers affiliated with at least one African institution, with contributions from South Africa (6/11, 55% studies) [90-95], Nigeria (4/11, 36%) [95-98], Guinea (1/11, 9%) [99], and Rwanda (1/11, 9%) [100]. In addition, 4 (36%) studies involved collaborations with international researchers from institutions based in the United States (3/11, 27% studies), Canada (3/11, 27%), Germany (1/11, 9%), and Mexico (1/11, 9%). Funding was disclosed in 6 (55%) of the 11 studies, all supported by public entities.

The primary NLP applications developed in these studies were conversational assistants (4/11, 36% studies) and sentiment analysis tools (3/11, 27% studies). These studies primarily supported EPHF 7 (ie, health promotion, 8/11, 73% studies). Regarding language coverage, nearly all studies (11/11, 100%) reported support for English. A smaller number addressed African languages, including Ndebele (2/11, 18% studies), Sotho (2/11, 18%), Kiswahili (2/11, 18%), Swati (2/11, 18%), Venda (2/11, 18%), Xhosa (2/11, 18%), Zulu (2/11 18%), with one study each for Afrikaans, Hausa, Kinyarwanda, Northern Sotho, Shona, Tsonga, and Tswana. For target countries, Nigeria and South Africa were the primary focus, each covered in 5 (45%) studies. Notably, none of the studies provided performance evaluations based on health-related measures or reported reaching the stage of actual deployment.

Outside academia, commercial products and NGO-led initiatives have focused on creating practical NLP solutions aimed at real-world public health impact. On the basis of our search results, 4 NLP technologies were developed as commercial products by companies [101-104], and another 4 were created as part of initiatives led by NGOs [105-108]. These projects were often in partnership with charitable organizations like the Bill and Melinda Gates Foundation, international bodies, such as the WHO, and industry partners like Google or Meta, frequently collaborating with telecom providers to reach populations with lower literacy levels and limited access to public health resources. The primary focus of these NLP technologies was on disseminating public health information through conversational assistants, with applications supporting EPHF 7 (ie, health promotion) and SDG 3 (good health and well-being). Most tools were designed in English with the limited inclusion of widely spoken African languages like Hausa, Kiswahili, and Zulu. In contrast to academic literature, NLP technologies from these nonacademic sources typically disclosed only limited details about their design and implementation.

Furthermore, our review of events and conferences did not introduce additional evidence of NLP technologies tailored to African public health challenges. A lack of standardized protocols for reporting NLP technologies, such as established reporting standards or controlled vocabularies for indexing, may explain why no relevant NLP technologies were retrieved during our search, likely due to limited keyword overlap.

## Discussion

### Principal Findings

Research into NLP technologies for public health in Africa is an emerging field, with significant growth since 2019. Current studies primarily focus on 2 applications: conversational agents for public health information dissemination and sentiment analysis tools that track public health attitudes on social media. Most studies target high-resource languages like English, Arabic, and French, with limited support for widely spoken African languages, such as Kiswahili and Zulu, and no support for most of Africa’s >2000 languages.

Most NLP applications remain in the prototype stage, with evaluations often limited to technical performance metrics in controlled settings. Only a handful of studies have validated their systems in real-world contexts, and just 1 has reached full deployment. Until now, most systems have been developed as technical NLP tools rather than targeted health interventions, with limited evaluation of their impact on public health outcomes through rigorous study designs and implementation research approaches.

While current research highlights the potential of NLP to address public health needs in Africa, this potential remains largely unrealized in terms of measurable public health outcomes. The following discussion explores pathways for public health and NLP researchers to contribute to the development and deployment of NLP technologies toward achieving positive health impacts in Africa. In addition, we reviewed the strengths and limitations of our review approach, providing context for readers to critically evaluate the subsequent discussion.

### Bridging Technical NLP Performance With Health-Related Outcomes

The review of 54 studies highlights the growing effort to leverage NLP technology for health improvement in Africa. However, it identifies a significant gap in evaluating real-world health outcomes or the behavioral antecedents of these outcomes. Most studies (51/54, 94%) emphasized technical performance, using metrics, such as accuracy, precision, *F*_1_-score, and recall. In comparison, only 11 (20%) studies incorporated user-centered evaluations, such as usability testing or health care provider feedback. While some studies [45,47,54] assessed user outcomes like the accuracy of health communications and improvements in health care interactions, only 2 (4%) studies [71,89] measured explicit health-related impacts. Specifically, 1 (2%) paper [71] demonstrated improvements in participants’ mood through an automated intervention targeting maternal mental health in Kenya, while another paper [89] showed increased vaccine willingness via a chatbot addressing individual concerns. These examples illustrate the potential of NLP interventions to influence public health, while their rarity highlights the need for more research focused on evaluating health impacts.

An overreliance on technical NLP metrics limits our understanding of whether these technologies effectively address real-world health challenges. To ensure NLP solutions meet their intended public health goals, future research should incorporate tools to evaluate health-related measures and behavioral outcomes of NLP solutions alongside technical performance. Tools and frameworks already exist to guide the evaluation of health interventions, such as the WHO’s “Monitoring and Evaluating Digital Health Interventions” framework [113], which provides standardized guidelines for assessing the impact of digital health technologies on health outcomes and behaviors. Despite the availability of such resources, they remain underutilized in the evaluation of NLP technologies. To fully realize the potential of NLP for public health, it is essential that future studies adopt these established frameworks to rigorously measure both health outcomes and behavioral changes. Integrating these tools will strengthen evidence on the real-world effectiveness of NLP interventions and support more impactful, data-driven public health strategies.

### Deployment, Integration, and Cross-Sectoral Development

For NLP technologies to be deployed in an impactful way, they must be integrated into African health systems and broader public health infrastructure, ensuring accessibility to diverse groups of users. The results of our review of the academic literature have shown the nascent nature of NLP deployment in Africa, with only 1 technology, a Facebook messenger chatbot collecting data on vaccine hesitancy [89], having reached full deployment. Other described technologies (40/50, 80%) were designed with the potential for integration into public health systems, and most apps under development are available without significant restrictions (ie, either open-source or publicly available). However, many of these apps require substantial expertise in computer science for installation and use, limiting their accessibility. For effective integration, these technologies need to be accessible to their intended users, such as health care workers, patients, and nonspecialists. Approximately 20 apps in this review were designed to be delivered via mobile- or web-based interfaces, increasing their potential usability.

In contrast, industry-led commercial products and NGO-driven initiatives have generally progressed further, often yielding immediate, tangible impacts for African communities. These initiatives commonly partner with organizations [114] like the Bill and Melinda Gates Foundation [115], the WHO, and companies [116], such as Google or Meta, and frequently collaborate with telecom providers to enhance accessibility for populations with limited resources and lower literacy levels. Unlike academic studies, which typically prioritize proof-of-concept and feasibility testing, these projects aim for direct public health impact, real-world validation, and, at times, profitability. However, as highlighted in this review, nonacademic projects tend to focus on narrower applications, primarily conversational assistants, offer limited language support, serve smaller populations, and address a more focused range of public health challenges compared to the diverse objectives often seen in academic research.

Moving forward, bridging the gap between NLP research and accessible, real-world applications will be essential for delivering positive public health impacts. The narrower focus of nonacademic projects highlights a need for extended collaboration between academic and nonacademic researchers, combining priorities, expertise, and resources to enhance NLP’s potential in addressing Africa’s public health needs. Cross-sectoral partnerships offer a promising model for advancing academic NLP technologies from proof-of-concept to impactful public health solutions across the continent.

### Toward Needs-Based Approaches

As we move toward the SDGs’ 2030 deadline, it is sobering to note that “current progress falls far short of what is required to meet the SDGs” [117]. Within this, the world is off track to achieve SDG 3 [117]. The SDG dashboard map offers a country-by-country breakdown of each of the SDG 3 indicators [118]. Progress toward SDG 3 in all but one mainland African country (ie, Tunisia) is described as “major challenges remain” (ie, the most concerning category), while Tunisia and the island nations of Cabo Verde, Mauritius, and the Seychelles are in the less severe category of “significant challenges remain.” In terms of progress, no African countries are currently considered to be “on track” or “decreasing” their progress; instead, they are all described as having major challenges in their progress toward the SDG 3 (ie, good health and well-being) targets [3].

In response to this somewhat bleak outlook, the United Nations prescribes that “changing course requires prioritizing the achievement of universal health coverage, strengthening health systems, investing in disease prevention and treatment, and addressing disparities in access to care and services, especially for vulnerable populations” [117]. Furthermore, it should be recognized that poverty and inequality constrain the possibilities for health gains [119], highlighting the need for a paradigm focused not only on treatment but on prevention, equity, and intersectional, multisectoral approaches to health promotion.

There is also a need to address technological and infrastructural limitations which still exist. Globally, a third of people remain offline—that is 30% of men and 35% of women [117]. In 2015, 15.6% of people in sub-Saharan Africa had internet access, rising to 37% by 2023 (*ibid*). Furthermore, a study of 15 countries (of which 7 were African countries) demonstrated how access to this technology often varies, with lower phone ownership in rural compared to urban areas, and varied ownership levels between poorer and wealthier income groups [120].

A review of the successes and limitations of telemedicine deployment in Africa during the COVID-19 pandemic [121] demonstrates what this means in practice. The study found the following technologies were used “videos, telephones, smart wearable digital devices, messaging mobile apps, virtual programs, online health education modules, SMSs, live audio–visual communication, and other digital platforms.” Among these, phones were the most widely used. Some of the difficulties faced included an array of digital challenges ranging from low connectivity and high data costs to the inaccessibility of smartphones, nondelivery of messages, and insufficient digital skills. This was in a broader context characterized by a lack of telemedicine frameworks and policies to support a roll out; some patients and health care personnel preferred not to use these technologies, and there was an underlying shortage of health care personnel [121].

For NLP technologies to address real-world health challenges, they should be viewed not just as technical solutions but as tools shaped by and responsive to the local context. Developing effective NLP applications will require a community-centered approach [122,123], grounded in local needs, ethical principles, infrastructures, and capacities to ensure these tools are truly accessible and impactful. Engaging people in research, including coresearchers, can facilitate a closer understanding of local needs and suitable ways to address these [124].

### The Need for Culturally and Linguistically Inclusive NLP Applications in Africa

Africa has exceptional linguistic diversity, with >2000 languages spoken across the continent [21,22]. This includes widely used official languages, such as Arabic, English, and French, alongside popular indigenous languages, such as Zulu, as well as a large majority of underrepresented languages spoken by smaller communities. Kiswahili spans several African countries, uniting East Africa as the shared language of politics, trade, music, literary tradition, and religion (both Islam and Christianity) [125]. Nigeria is the most linguistically diverse, with >500 indigenous languages [126]. While official languages tend to have relatively sufficient digital data to support NLP development, most indigenous African languages fall into the category of being low-resource, extremely low-resource, or even no-resource, often lacking any digital data essential for NLP technologies. The scarcity of digital language resources forms significant performance disparities in NLP systems [127]. These disparities, including higher error rates for underrepresented languages (ie, error rate disparities [128]), contribute to broader inequities, limiting access to advancements in NLP technology and impeding speakers of underrepresented languages from fully benefiting from progress in NLP technology.

To develop inclusive NLP applications that equitably serve African populations, strategically expanding digital datasets for underserved languages is essential. This is particularly the case for languages with limited online representation [129]. Concurrently, advancements in multilingual NLP and cross-lingual transfer learning provide promising opportunities [130-132]. These approaches allow neural language models, the backbones of most modern NLP applications, to leverage knowledge from high-resource languages to perform well in low-resource contexts, even with minimal in-language data. By combining efforts in data collection with advancements in NLP research, these technologies can better support Africa’s linguistic diversity, contributing to public health solutions that promote, rather than hinder, health equity.

In addition to linguistic inclusivity, cultural relevance is essential for NLP technologies [29,133] to meet the diverse needs of African communities effectively. Distinct cultural practices, health beliefs, and communication styles influence how groups perceive and interact with technology.

Embedding cultural practices into the design and implementation of NLP technologies is essential for ensuring their relevance and successful integration into Africa’s health systems and institutions. For example, a health education dialogue system advising users to visit a *general practitioner*, a term commonly associated with primary care doctors in the United Kingdom’s National Health Service, may be irrelevant or confusing to many users in Africa. Furthermore, Loveys et al [134] highlighted how expressions of depression in text, such as the ratio of positive to negative emotions, vary across cultures. Similarly, the extensive use of traditional medicine in many African countries [135] highlights the need for NLP technologies to incorporate culturally recognized terms and references to these practices. Another good example is the recent work by Olatunji et al [136], which introduces a geo-culturally diverse dataset of clinically diverse questions and answers annotated by health experts. This dataset enables the development of question-answering systems serving African patients.

Respecting and integrating these cultural nuances into NLP design and implementation can enhance trust, ensure alignment with the expectations and needs of each community, and ultimately promote equitable public health outcomes.

### Integrative Development for NLP in Public Health

Our analysis shows that NLP technologies focusing on health in Africa intersect most frequently with SDG 10 (ie, reduced inequality) and SDG 9 (ie, industry, innovation, and infrastructure). This study did not identify any intersections with other SDGs, even those that are particularly relevant to public health, such as SDG 1 (ie, end poverty), SDG 2 (ie, zero hunger), SDG 6 (ie, clean water and sanitation), SDG 7 (ie, affordable and clean energy), SDG 11 (ie, sustainable cities and communities), SDG 12 (ie, responsible consumption and production), SDG 13 (ie, climate action), and SDG 17 (ie, partnership for the goals) are absent. This highlights an opportunity for more cross-cutting intersections between NLP applications for health and broader sustainable economic development efforts, as well as the importance of a more integrated approach to achieving the SDGs [112,137].

The current coverage of the SDG 3 targets and means of implementation by existing NLP technologies (Figure 3) highlights the need for more investment in the WHO framework convention on tobacco control (SDG 3.a), substance abuse (SDG 3.5), road traffic (SDG 3.6), and environmental health (SDG 3.9) for sustained and long-term health impacts. While the goals of ending epidemics (SDG 3.3), reducing maternal mortality (SDG 3.1), and achieving universal health coverage (SDG 3.8) are strongly represented in the literature, there remains significant potential to invest more in cross-cutting activities that have long-term impacts on health systems.

The limited attention given to 6 key EPHFs (ie, highlighted by the orange line in Figure 2) also highlights a significant gap in the research landscape, with critical public health functions, such as emergency management, stewardship, and multisectoral planning being overlooked. These underaddressed EPHFs and SDG 3 targets play a fundamental role in building resilient health systems, especially in low-resource settings. Therefore, there is a need for more balanced research efforts to ensure all aspects of public health are adequately supported.

Developing NLP technologies to address these underresearched EPHFs and SDG 3 targets requires a deep understanding of local contexts and the integration of NLP technologies within them. For instance, addressing EPHF 12 (ie, access to health products) requires integrating NLP technologies into existing logistics and supply chain systems, which often vary significantly between countries. Such highly specialized and localized contexts introduce additional challenges and requirements for system development.

Future studies should aim to bridge these gaps by aligning NLP development with more integrative, cross-disciplinary collaborative approaches that promote system-wide impact. By embedding NLP technologies within broader health system goals and leveraging collaborative input across fields, these solutions can become more effective, resilient, and responsive to public health challenges. Such an approach will help guide interventions and inform policy development, ensuring that public health improvements are enduring and responsive to Africa’s evolving health needs.

### Strengths and Limitations of the Review

This review has several strengths. We developed and followed a protocol guided by the PRISMA-P (Preferred Reporting Items for Systematic Review and Meta-Analyses—Protocols) guidelines [138], ensuring a transparent and systematic approach throughout the study. Our literature search was comprehensive and multidisciplinary, integrating both academic and gray literature sources to capture a broad range of perspectives and to provide a current reflection of the scope of research. The inclusion of gray literature, such as reports from industry and NGOs, enabled a wider view of NLP applications in public health that extends beyond the academic literature. Screening and data extraction were conducted following predefined guidelines created by a team of domain experts, enhancing both the consistency and relevance of the data collected. The extracted data is publicly available in a machine-readable format to facilitate future research (Multimedia Appendix 3).

However, there are some limitations to consider. Our review relied primarily on English search terms, which may have inadvertently excluded studies published in other languages without English annotations. Although we included 5 representative databases spanning public health, NLP, computing, and engineering, some relevant studies may have been missed because of the highly interdisciplinary nature of the field. In addition, NLP research is frequently disseminated as preprints, blog posts (eg, OpenAI’s introduction of ChatGPT), and in peer-reviewed machine learning conference proceedings (such as International Conference on Learning Representations and Conference on Neural Information Processing Systems) not indexed by our selected databases. Consequently, certain emerging studies may not have been captured, especially given the rapid development of NLP applications in public health. However, the results of our gray literature search indicate that most academic papers, including preprints and conference proceedings, have been covered in this review.

Although this review included a broad range of gray literature, the search for nonacademic sources was far from exhaustive, and some relevant NLP technologies may have been overlooked. The primary focus of this review was on peer-reviewed academic literature, with gray literature providing supplementary insights. This work could be further expanded through a series of expert interviews, such as with representatives from the WHO or the SDG by 2030 committees, to interrogate the factors shaping the development, or lack thereof, of NLP applications for public health in Africa [139,140]. In addition, the heterogeneity of NLP methodologies, applications, and evaluation metrics hindered our ability to conduct a formal meta-analysis, resulting in a reliance on narrative synthesis. Despite efforts to maintain consistency during data extraction, inconsistencies in reporting approaches for included studies presented challenges, particularly in evaluating public health focus and outcomes, which impacts comparability across different technologies.

These challenges reveal a broader misalignment between the rigorous methodological standards of systematic reviews and the agile, fast-evolving, and highly interdisciplinary nature of NLP research. Future research should explore methodological adaptations that better align with the field of NLP, especially for applications in health contexts.

### Conclusions

The application of NLP technologies to public health in Africa is a promising and rapidly evolving field, with the potential to enhance health care accessibility, equity, and efficiency across the continent. However, significant gaps persist in real-world deployment, language inclusivity, and the rigorous evaluation of health outcomes. The identification of, and responses to, such gaps would be greatly enabled by the establishment of reporting standards for NLP technologies.

Future research should adopt a needs-based and cross-sectoral approach, engaging expertise from diverse fields, including the expertise of local communities on their own needs and possible solutions, and using existing frameworks for evaluating their public health impacts and outcomes. This approach will help build a deeper understanding of needs and support the tailored design of NLP technologies to effectively address public health challenges where this technology can be useful and is wanted. Furthermore, qualitative research, such as expert interviews, can contribute to better understand the dynamics and demand for progress in this area. By bridging existing gaps in meaningful local engagement, NLP research can better support resilient, culturally relevant, and equitable public health systems in Africa.

## Appendix

Multimedia Appendix 1 Definition of natural language processing, search strategy, screening guidelines, data extraction template, results, and supplementary materials.

Multimedia Appendix 2 The PRISMA-ScR (Preferred Reporting Items for Systematic Reviews and Meta-Analyses Extension for Scoping Reviews) checklist for the scoping review.

Multimedia Appendix 3 Extracted data from included studies in JSON format.

## Abbreviations

AI: artificial intelligence
BERT: Bidirectional Encoder Representations
EPHF: essential public health function
GPT-2: Generative Pre-trained Transformer 2
NGO: nongovernmental organization
NLP: natural language processing
PRISMA-P: Preferred Reporting Items for Systematic Review and Meta-Analyses—Protocols
PRISMA-ScR: Preferred Reporting Items for Systematic Reviews and Meta-Analyses Extension for Scoping Reviews
SDG: sustainable development goal
WHO: World Health Organization
