# Supplementary material for: Natural Language Processing Technologies for Public Health in Africa: Scoping Review
Source: J Med Internet Res. 2025 Mar 5;27:e68720. doi: 10.2196/68720 (PMC11923465; doi:10.2196/68720)
Supplement: Multimedia Appendix 1 [file jmir_v27i1e68720_app1.pdf]

Multimedia Appendix: Definition of NLP, Search Strategy, Screening Guidelines, Data Extraction Template and Results, and Supplementary Materials

Contents

1 Introduction **2**

2 Definition of NLP Technologies **2**

3 Search Strategy for Academic Literature **4**

3.1 Choices of Databases 4

3.2 Search Strategy 4

3.3 Search Dates and Publication Period: 5

3.4 Language and Publication Restrictions 5

4 Search Strategy for Grey Literature **5**

5 Screening Guidelines **6**

5.1 Title and Abstract Screening 6

5.2 Full-Text Screening 7

6 Data Extraction Template **8**

6.1 Study Description and Metadata 8

6.2 Linguistic and Geographic Coverage 9

6.3 NLP Application and Evaluation 9

6.4 Public Health Functions and Impact 10

6.5 SDGs Coverage 11

6.6 Timeliness and Deployment 11

6.7 Ethical Considerations and User Engagement 13

7 Categorisations of Included Studies **13**

8 Companies, NGOs, Events, and Research Communities in Literature Search **15**

8.1 Startups and Established Companies 15

8.2 Initiatives and NGOs 15
8.3 Events and Conferences 16
8.4 Events and Conferences 16
8.5 African NLP Research Communities 16

9 Data Extraction Results **17**
9.1 Academic Literature 17
9.2 Grey Academic Literature 71
9.3 Commercial Products and Initiatives 82

A Complete Search Terms for MEDLINE via PubMed **95**

1 Introduction

We have conducted a scoping review on the application of natural language processing (NLP) technologies that are available to support public health in Africa. NLP is the field of research for a range of computational techniques that enable computers to process and understand human languages, thereby facilitating a variety of tasks such as text analysis and language generation. An example NLP application is a virtual assistant, such as ChatGPT[1] by OpenAI and Siri[2] by Apple. In the context of public health, these technologies are employed to improve the accessibility, personalisation, and efficiency of health services. For example, IBM has developed a medical assistant[3] with NLP technologies to provide more accessible healthcare services. For an operational definition of NLP technologies within the context of this review, please see Section 2.

In this scoping review, we are interested in any application that utilises NLP technologies to process language data derived from public health settings to enhance public health outcome in Africa. This includes, but is not limited to, patient records, medical literature, and health monitoring data. We focus on systems developed in a data-driven manner that are tailored to the linguistic and cultural contexts of African communities. This scoping review aims to answer five research questions:

1. **Needs and Availability:** What public health needs are being addressed by NLP technologies in Africa, and what unmet needs remain?
2. **Prevalence and Distribution:** What factors influence the availability of public health NLP technologies across African countries and languages?
3. **Deployment and Integration:** What stages of deployment have these technologies reached, and to what extent have they been integrated into existing health systems?
4. **Public Health Impact:** What measurable impact have these technologies had on public health outcomes, where such data are available?
5. **Outlook:** What recommendations have been proposed to enhance the quality, cost, and accessibility of health-related NLP technologies in Africa?

2 Definition of NLP Technologies

For the purposes of this review, we define NLP technologies broadly to include **any computational systems that process natural language, either as input or output**. This includes, for example, dialogue systems that interpret user utterances and generate responses in natural language.

Additionally, our interest extends to **computational and digital resources** that support the development of NLP technologies. For example, **digital datasets, computer hardware, and software**

[1] `https://openai.com/chatgpt/`
[2] `https://www.apple.com/uk/siri/`
[3] `https://www.ibm.com/products/watsonx-assistant/healthcare`

toolkits are included within the scope of NLP technologies for this review. These components, crucial for the development and deployment of NLP systems, are considered parts of NLP technologies.

In the following, we will provide examples of systems that are not considered NLP technologies within the scope of this review.

Example 1

Consider a system designed to predict medical outcomes from survey results, where the questions in the survey are phrased in natural language, such as:

- Has the patient experienced any fever since the surgery? (yes/no)
- Has the patient experienced pain around the incision? (yes/no)

Example 1 is recognised as an NLP technology within this review. Not all NLP technologies directly process textual data as input or output. For instance, the implementation of the system described in Example 1 may only handle responses to questions presented as binary labels, rather than the natural language text of the questions themselves. The data this system is designed to process, however, originate in natural language. Our primary concern is not the specific implementation details of these systems; instead, we focus on their capability to process natural language data in practical applications.

Example 2

Consider an Optical Character Recognition (OCR) system that inputs images of documents and outputs the text contained within those documents.

Example 2 illustrates a typical NLP technology. Although the input for this technology is an image, not natural language, the output is natural language. The primary function of this system involves transforming visual data into textual format.

Example 3

Consider a dataset containing 2000 dialogues between patients and doctors discussing a new type of vaccine. These dialogues capture doctors responding to patient inquiries about various aspects of this vaccine.

Example 3, which is a digital language dataset, is considered part of NLP technologies for the purposes of this review. While this example primarily consists of a dataset and does not directly involve the application of a computer program, the digital resource it provides is essential for the development of future NLP technology. Thus, it falls within our scope of interest.

Example 4

Consider a system that analyses medical images of patients to predict the risks of certain diseases.

Example 4 is not considered an NLP technology in this review. While the system may employ advanced machine learning methods, it does not involve natural language processing as defined in this review because the system does not include converting or processing natural language data.

Example 5

Consider a system that predicts the risks of certain diseases based on a set of structured features. These features may include:

- the temperature of the patient: 37.2°C
- allergies: none

Example 5 is not considered an NLP technology in this review. Unlike Example 1, which involves natural language in its initial data construction, Example 5 processes structured data instead of natural language data, thereby excluding it from our definition of NLP technology.

3 Search Strategy for Academic Literature

3.1 Choices of Databases

We searched the following well-established electronic bibliographic databases:

- MEDLINE via PubMed[4]: medical and public health literature.
- ACL Anthology[5]: NLP and language science literature.
- Scopus[6]: broad interdisciplinary scope, including medical research.
- IEEE Xplore[7]: engineering literature, particularly in NLP and health informatics.
- ACM Digital Library[8]: computing literature, including NLP and health informatics.

During our initial search phase, we excluded preprint servers such as arXiv[9] and bioRxiv[10], and conferences, such as ICML[11] and ICLR[12], which do not offer dedicated search engines for their proceedings. We included a subsequent phase of grey literature searches, however, using Google Scholar[13]. This allowed us to capture relevant papers from these excluded sources. For details, please refer to Section 4.

3.2 Search Strategy

Our search strategy integrated search terms related to the three key concepts of our review: **African communities**, **public health**, and **NLP**. Specifically:

- **Africa:** including the names of all 55 African Union member countries[14] and African languages with over 1 million native speakers[15].
- **Public Health**: based on the 12 EPHFs[16] outlined by the WHO.
- **NLP:** as suggested by a team of experts in the field.

These terms were combined with general phrases and MeSH headings. The search strategy for each database was refined with database-specific features to enhance the retrieval of relevant studies. The complete search strategy for MEDLINE (PubMed) and the full list of search terms are detailed in Appendix A.

---

[4] https://pubmed.ncbi.nlm.nih.gov
[5] https://aclanthology.org
[6] https://www.scopus.com
[7] https://ieeexplore.ieee.org
[8] https://dl.acm.org
[9] https://arxiv.org
[10] https://www.biorxiv.org
[11] https://icml.cc
[12] https://iclr.cc
[13] https://scholar.google.com
[14] https://au.int/en/member_states/countryprofiles2
[15] Statistics are based on https://www.ethnologue.com.The names of African languages with more than 1 million native speakers. This decision to use this specific threshold is to avoid common English words that are also names of African languages, such as "Siri", "So", and "Day". Including all the 2,220 documented African languages would retrieve a large number of irrelevant papers and significantly decrease the precision of our search results. Besides, our search terms focus on the names of languages and countries rather than specific demographic groups. This approach aligns with the practice of developing and deploying NLP technologies: language adaptation and localisation.
[16] https://www.who.int/teams/primary-health-care/health-systems-resilience/essential-public-health-functions

3.3 Search Dates and Publication Period:

- Initial search cut-off: January 2013
- First search date: 13 May 2024
- Update search date: 3 October 2024

3.4 Language and Publication Restrictions

There were no restrictions based on the language of publication. However, it is worth noting that our search terms were in English.

4 Search Strategy for Grey Literature

In our review, we conducted searches for grey literature on four resources: 1) **preprints, non-peer reviewed studies, and reports**, 2) **media articles and blog posts**, 3) **commercial products from startups and established companies**, 4) **initiatives led by non-profit organisations**, and 5) **proceedings and presentations from events and conferences**.

Preprints, Non-Peer Reviewed Studies, and Reports. Wwe conducted a search on Google Scholar[17]. We applied the following search terms: "natural language processing", "health", and "Africa" and reviewed the first 100 results returned by the search. We applied the same two-stage screening process (title and abstract, followed by full text) as our structured database searches.

Media Articles and Blog Posts. We conducted a search on Google Search[18] with the following search terms: "natural language processing", "health", "Africa", "article", and "blog". We reviewed the first 100 results and conduct a two-stage screening process (title and snippet, followed by full text). The snippet is the brief explanation or snapshot provided below the title in the search results.

Commercial Products from Startups and Established Companies. We searched for startups and companies on the following platforms: Crunchbase[19], Angellist[20], VC4Africa[21], Disrupt Africa[22], and Startuplist Africa[23]. Due to the different designs of these platforms, our search was adapted to each platform's specific search capabilities, rather than taking a uniform approach. The exact search strategies, including the keywords used, will be documented in our review paper. Additionally, we searched for NLP startups and companies in Africa, by conducting a search on Google Search with the search terms: "natural language processing", "health", "Africa", "startup", and "company". From the search results, we reviewed the first 100 results and conducted a two-stage screening process (title and snippet, followed by full text).

Initiatives Led by Non-Profit Organisations. Through Google Search, we searched for initiatives and non-profit organisations in Africa focusing on developing NLP applications for health. The search terms were: "natural language processing", "health", "Africa", "initiative", and "organisation". We reviewed the first 100 search results and conducted a two-stage screening process (title and snippet, followed by full text).

Proceedings and Presentations from Events and Conferences. We conducted a search on Google Search for events and conferences focused on developing NLP applications for health in Africa. The search terms used were: "natural language processing", "health", "Africa", "event", and "conference". We reviewed the first 100 search results and employed a two-stage screening process (screening by title and snippet, followed by a full-text review).

[17] `https://scholar.google.com`
[18] `https://www.google.com`
[19] `https://www.crunchbase.com`
[20] `https://www.angellist.com`
[21] `https://vc4a.com/search/`
[22] `https://disruptafrica.com`
[23] `https://startuplist.africa`

5 Screening Guidelines

To select relevant evidence to answer the aforementioned research questions, we will undertake two tasks: title and abstract screening, and full-text screening.

5.1 Title and Abstract Screening

In this initial screening phase, we evaluate a list of papers based **solely** on their titles and abstracts. Our objective is to **independently** determine whether each paper meets the specified exclusion criteria. Papers that fulfil **any** of the exclusion criteria should be **rejected**.

Exclusion criteria:

- **Non-NLP Applications**, when no language technologies are involved, and the technology is used to do other tasks such as predict outcomes solely from structured datasets or images.
- **Non-Health Applications**, including when NLP technologies are used for public health but were not developed for health purposes (e.g., general-purpose machine translation systems).
- **Non-African Contexts**, where the focus is on settings outside Africa, except where such studies offer comparative insights relevant to African NLP technologies.

To enhance the precision of our screening process and prevent the inclusion of a large number of machine learning studies that are irrelevant to NLP, it is required that the title or abstract explicitly provide evidence that the systems discussed involve natural language as either input or output. Papers must demonstrate this involvement to be considered for inclusion and not rejected.

In this review, we recognise African languages as those spoken within the continent by African populations, including both indigenous languages and widely used official languages such as Arabic, English, French, and Portuguese. Therefore, systems that support only English may still be relevant to our objectives and should not be rejected.

In the NLP literature, it is common for papers not to specify the languages their models or systems support, often stating that they support multiple languages without listing them. During the title and abstract screening, papers should be included unless there is clear evidence that they are not relevant to African languages or African users. For instance, papers should be excluded if they explicitly state they are discussing public health issues outside of African contexts, such as in the UK. This method ensures we do not prematurely exclude studies that could potentially contribute to our review. Decisions on ambiguous cases should be deferred until the full-text screening phase.

If the title and abstract provide sufficient evidence that the paper meet any of the exclusion criteria, mark it as "false". If there is insufficient evidence to reject a paper or make a decision, mark the paper as "true" to ensure it proceeds to the next stage of full-text screening. Do not access the full text of the paper during this task.


```
{
    "title": "Machine Learning based Malaria Prediction using Clinical Findings",
    "year": 2021,
    "venue": "2021 International Conference on Emerging Smart Computing and Informatics (ESCI)",
    "url": "https://ieeexplore.ieee.org/stamp/stamp.jsp?arnumber=9396850",
    "database": "ieee",
    "abstract": "Even today, Malaria is the most deadly disease in Asia and sub-Saharan Africa and particularly in Senegal. This is mainly due to
     inadequate medical care support with frequent late and error-diagnoses by medical professionals. Besides, mostly used diagnostic standards such as
     the rapid diagnostic test is not fully reliable. With the development and widespread acceptance of automated systems in the healthcare system,
     machine learning algorithms can support medical professionals in their decision-making procedure. An experimental analysis of different machine
     learning techniques to predict Malaria is proposed in this work. These techniques attempt to determine whether or not a patient suffersfrom Malaria
      using various clinical findings like signs and symptoms. The algorithms' efficiency has been thoroughly validated and analysed over two actual
     data sets of malaria patients' taken from Senegal. The results obtained show that Random Forest, Support Vector Machine with Gaussian Kernel and
     Artificial Neural Networks are promising and offer the best overall accuracy to predict the appearance or not of the disease with precision, recall
      and F1-score at least equal to 92%, 85% and 89% respectively on both datasets on which they outperform the Rapid Diagnostic Test.",
    "should_include": "false",
    "task_id": "62"
},
```


Figure 1: An example paper entry for the title and abstract screening task. If the paper meets all inclusion criteria and none of the exclusion criteria, update the field "should_include" to "true".

Tasks are provided as a list of paper entries formatted in JSON format, which facilitates easier processing by computers. An example entry is shown in Figure 1. For each paper, every field should be thoroughly reviewed and a judgement made based on the criteria provided.

The following are examples of papers that should or should not be included in the title and abstract screening process.


```
{
    "title": "Hypertension Diagnosis and Management in Africa Using Mobile Phones: A Scoping Review",
    "year": 2024,
    "venue": "IEEE Reviews in Biomedical Engineering",
    "url": "https://ieeexplore.ieee.org/stamp/stamp.jsp?arnumber=9809807",
    "database": "ieee",
    "abstract": "Target 3.4 of the third Sustainable Development Goal (SDG) of the United Nations (UN) General Assembly proposes to reduce premature mortality from
     non-communicable diseases (NCDs) by one-third. Epidemiological data presented by the World Health Organization (WHO) in 2016 show that out of a total of 57 million deaths
      worldwide, approximately 41 million deaths occurred due to NCDs, with 78% of such deaths occurring in low-and-middle-income countries (LMICs). The majority of
     investigations on NCDs agree that the leading risk factor for mortality worldwide is hypertension. Over 75% of the world's mobile phone subscriptions reside in LMICs,
     hence making the mobile phone particularly relevant to mHealth deployment in Africa. This study is aimed at determining the scope of the literature available on
     hypertension diagnosis and management in Africa, with particular emphasis on determining the feasibility, acceptability and effectiveness of interventions based on the
     use of mobile phones. The bulk of the evidence considered overwhelmingly shows that SMS technology is yet the most used medium for executing interventions in Africa.
     Consequently, the need to define novel and superior ways of providing effective and low-cost monitoring, diagnosis, and management of hypertension-related NCDs delivered
     through artificial intelligence and machine learning techniques is clear.",
},
```


Figure 2: Example for title and abstract screening. This paper should be included (not rejected).

The example paper in Figure 2 should be included. Its title explicitly mentions Africa and healthcare, thus not meeting the exclusion criteria for non-health applications and non-African contexts. Additionally, the mention of SMS technology in the abstract suggests potential relevance to processing textual data with computer programs, indicating the involvement of NLP.


```
{
    "title": "Disparities in a provision of in-hospital post-arrest interventions for out-of-hospital cardiac arrest (OHCA) in the elderly population-protocol for a systematic
      review",
    "year": 2016,
    "venue": "Syst Rev",
    "url": "",
    "database": "pubmed",
    "abstract": "BACKGROUND: Out-of-hospital cardiac arrest (OHCA) is a significant cause of \ndeath in developed countries. The majority of OHCA patients are elderly (≥65
    \nyears), and it was documented that they were less likely than younger patients \nto receive the evidence-based interventions, even though the improvement in \nsurvival
    in the elderly age group was higher than in younger population. Our \ngoal is to investigate any disparity in the provision of post-arrest care for \nthe elderly with
    OHCA and a sustained return of spontaneous circulation (ROSC).\nMETHODS/DESIGN: Eight relevant, electronic databases will be systematically \nsearched to identify
    eligible studies. The searches will be supplemented with \ngray literature searching of theses, dissertations, and hand searching of \npertinent journals. Two independent
     reviewers will screen the titles and \nabstracts and select studies for full text analysis using Preferred Reporting \nItems for Systematic Reviews and Meta-analyses
    (PRISMA) method, and both will \nextract information from the selected studies employing a form based on the Data \nExtraction Template for Cochrane Reviews. A team of
    three reviewers will assess \nthe quality of the studies with the modified Downs and Black scale. Statistical \nmethods for evidence synthesis, such as meta-analysis and
    meta-regression, will \nbe applied to compare and combine the evidence regarding the association between \nage and intervention provision/utilization, adjusting for a
    number of \nsignificant confounders, such as patient characteristics and co-morbidities and \navailability of intervention techniques, as well as study specific
    \ncharacteristics. The strength of evidence from the selected studies will be \nassessed using a modified Grades of Recommendation, Assessment, Development, and
    \nEvaluation (GRADE) system.\nDISCUSSION: The findings obtained from this systematic review should inform \nwhether disparity exists in the provision of post-arrest care
    for the elderly (≥ \n65 years old) with OHCA or not. Addressing this problem has a potential to \nsubstantially increase the number of > 65-year-old, long-term survivors.
     The \nresults of our review might also point to the gaps in the published literature \nthat specifically examines disparity in provision of care for this population.
    \nThis systematic review was designed in accordance with the Preferred Reporting \nGuidelines for Systematic reviews and Meta-analyses (PRISMA statement), while \nthe
    protocol follows the Preferred Reporting items for Systematic review and \nMeta-analysis protocols (PRISMA-P) statement.\nSYSTEMATIC REVIEW REGISTRATION: PROSPERO
    CRD42015027822.\n\nDOI: 10.1186/s13643-016-0234-4\nPMCID: PMC4853855\nPMID: 27142791 [Indexed for MEDLINE]",
},
```


Figure 3: Example for title and abstract screening. This paper should be rejected.

The example paper shown in Figure 3 should be rejected because both its title and abstract lack evidence of technologies involving the use of computer programs to process textual data. Additionally, our review focuses exclusively on technologies used or potentially usable by African communities. Although there is a possibility that the authors might employ computer programs for searching gray literature, this does not qualify as an NLP application within the scope of our review since it does not directly serve African communities.

5.2 Full-Text Screening

For this task, we review papers that were classified as "true" during the title and abstract screening phase. Here, we need to make **independent** evaluations based on the title, abstract, and the full text of each paper.

We define the following inclusion and exclusion criteria. The task is to find papers that satisfy our inclusion and exclusion criteria. All selected papers must meet these criteria.

Inclusion criteria:

- **Development** of NLP technologies specifically promoting public health in Africa.
- **Evaluations** of the effectiveness of NLP technologies in enhancing public health outcomes in Africa.
- **Adaptation** of existing NLP technologies to address public health challenges in Africa.

- **Resources**, such as digital datasets, hardware, or software toolkits, which support the development, evaluation, or adaptation of the aforementioned NLP technologies.

Exclusion criteria:

- **Non-NLP Applications**, when no language technologies are involved, and the technology is used to conduct other tasks such as the prediction of outcomes solely from structured datasets or images.
- **Non-Health Applications**, including when NLP technologies are used for public health but were not developed for health purposes (e.g., general-purpose machine translation systems).
- **Non-African Contexts**, where the focus is on settings outside Africa, except where such studies offer comparative insights relevant to African NLP technologies.


```
{
    "title": "Cross-Lingual Suicidal-Oriented Word Embedding toward Suicide Prevention",
    "year": 2020,
    "venue": "Findings of the Association for Computational Linguistics: EMNLP 2020 Association for Computational Linguistics",
    "url": "https://aclanthology.org/2020.findings-emnlp.200",
    "database": "acl",
    "abstract": "Early intervention for suicide risks with social media data has increasingly received great attention. Using a suicide dictionary
     created by mental health experts is one of the effective ways to detect suicidal ideation. However, little attention has been paid to validate
     whether and how the existing dictionaries for other languages (i.e., English and Chinese) can be used for predicting suicidal ideation for a
     low-resource language (i.e., Korean) where a knowledge-based suicide dictionary has not yet been developed. To this end, we propose a cross-lingual
      suicidal ideation detection model that can identify whether a given social media post includes suicidal ideation or not. To utilize the existing
     suicide dictionaries developed for other languages (i.e., English and Chinese) in word embedding, our model translates a post written in the target
      language (i.e., Korean) into English and Chinese, and then uses the separate suicidal-oriented word embeddings developed for English and Chinese,
     respectively. By applying an ensemble approach for different languages, the model achieves high accuracy, over 87{\\%}. We believe our model is
     useful in accessing suicidal ideation using social media data for preventing potential suicide risk in an early stage.",
    "should_include": "false",
    "task_id": "483",
    "reason_to_exclude": ""
},
```


Figure 4: An example paper entry for the full text screening task. The full paper can be accessed through the "url" provided. If the "url" field is empty, attempt to access the paper by searching its title. Similarly, if the paper meets all inclusion criteria and none of the exclusion criteria, update the field "should_include" to "true". For papers that do not meet the criteria, provide a brief explanation in the "reason_to_exclude" field. If there is insufficient evidence to make a definitive decision, update the field "should_include" to "unknow".

Figure 4 shows an example paper entry for this task. The full paper can be accessed through the "url" provided. If the "url" field is empty, attempt to access the paper by searching its title. Read through the full-text to evaluate whether the paper satisfies the inclusion and exclusion criteria. Annotate the paper as "true" if there is enough evidence to confirm that it meets the inclusion criteria. Annotate the paper as "false" if it does not meet the inclusion criteria. For papers that do not meet the criteria, provide a brief explanation in the "reason_to_exclude" field. If there is insufficient evidence to make a definitive decision, annotate the paper as "unknow". Papers marked "unknow" will be subsequently evaluated by another independent annotator or a domain expert.

You are encouraged to make a decision as soon as you find sufficient evidence to support your judgement. There is no requirement to read the entire paper if you have already gathered enough information to make an informed decision.

6 Data Extraction Template

Below, we outline the items of our data extraction form used for this scoping review. These items are grouped into five groups: study description and metadata, linguistic and geographic coverage, NLP application and evaluation, public health functions and impact, Sustainable Development Goals (SDGs) coverage, timeliness and deployment, and ethical considerations and user engagement.

6.1 Study Description and Metadata

1. **Paper Title:** Full title of the paper. Copy the title as it appears in the paper.

2. **Origin of the Work**: Record *all* the countries of the authors' affiliations using the Alpha-3 code listed in the ISO 3166 codes[24]. For instance, if an international team of researchers affiliated with the University of Cambridge publishes a paper, the country of affiliation is GBR.
3. **Affiliations**: Record *all* the authors' affiliations. For instance, if an international team of researchers affiliated with the University of Cambridge publishes a paper, the affiliation is the University of Cambridge.
4. **Types of Funding**: Types of funding received for the study. Select *all* funding sources from the following categories:
    - **Research Grant**: Funding provided by academic institutions or research councils.
    - **Industry Funding**: Financial support from private companies or industry stakeholders to promote research that aligns with their commercial interests.
    - **Government Funding**: Funds allocated by governmental bodies or public agencies to support research and development projects, often with a focus on public interest and policy objectives.
    - **NGO Funding**: Support provided by non-governmental organisations, which may include charitable foundations and international organisations.
    - **Private Funding**: Financial donations from individual philanthropists or private investors not directly affiliated with corporate or government entities.
    - **Others**: Any other types of financial support that do not fall into the above categories. For example, a study may be self-funded by the team of researchers without external funding.
    - **Not Mentioned**: The financial support is not disclosed in the paper.
5. **Continents of Funders**: Record the continent in which each funder is based. List *all* applicable continents if multiple funders supported the work. State "not mentioned" if the financial support is not disclosed in the paper.

6.2 Linguistic and Geographic Coverage

1. **Supported Languages:** Identify the set of languages supported by the NLP technology introduced in the paper. The languages should be documented using ISO 639-2 codes[25]. Different dialectal variants of a language (e.g., Arabic) are not distinguished.
2. **Target African Countries or Regions**: Identify specific African countries or regions where the introduced NLP technology is applied or intended to be used. Document the Alpha-3 code listed in the ISO 3166 codes[26] for countries or regions within the African continent. For example, if an NLP system is developed by researchers in the UK and deployed in the Republic of Kenya, complete the form with KEN.

6.3 NLP Application and Evaluation

1. **NLP Applications**: Document *one* specific NLP application each system performs, such as conversational assistant, language translation, or automated diagnosis.
2. **Evaluation Method:** Document how NLP systems are evaluated in each study. Select *all* that are applicable from the following categories:
    - **Technical Performance:**Intrinsic evaluation measures such as accuracy, precision, recall, and F1 score[27]. These are also referred to as intrinsic evaluation measures.
    - **User Experience:** Results on usability testing, user satisfaction surveys, and qualitative feedback from healthcare providers.
    - **Health-Related Measure:** Extrinsic evaluation measures such as patient engagement rates, reduction in diagnostic errors, or improvements in treatment outcomes. They are also referred to as extrinsic evaluation measures.
    - **Not Applicable**: The paper does not perform an evaluation of their NLP technologies or performance evaluation is irrelevant.

[24] https://www.iso.org/iso-3166-country-codes.html
[25] https://en.wikipedia.org/wiki/List_of_ISO_639-2_codes
[26] https://www.iso.org/iso-3166-country-codes.html
[27] F1 is the harmonic mean of precision and recall, especially useful for imbalanced data.

3. **Technical Evaluation Metrics**: Automatic evaluation metrics used to evaluate the NLP technologies (e.g., accuracy, F1 score). Denote "Not Applicable" if the paper does not perform an evaluation of a technology or if performance evaluation is irrelevant.
4. **System Performance**: Detail the results of the performance evaluation, noting both absolute values and performance relative to systems developed for resource-rich languages. Denote "Not Applicable" if the paper does not perform an evaluation of an NLP technology or if performance evaluation is irrelevant.
5. **Domain Coverage:** Document the domain of language resources that the NLP applications process. Select *all* that are applicable from the following categories:
   - **General Domain:** Data concerning general language processing outside specialised contexts.
   - **Research Domain:** Research articles and professional materials for expert audiences.
   - **Clinical Domain:** Clinical notes, patient interactions, and other healthcare-specific communications.
6. **Modality**: Type of data processed by the NLP application (e.g., text, audio, and/or image).

6.4 Public Health Functions and Impact

1. **Primary EPHF**: Determine *one* primary WHO's essential public health function[28] that each study addresses or impacts. If there are multiple EPHFs relevant in the paper, select the most relevant one. For example, if a study introduces an NLP system that monitors disease outbreak events based on social media data, the primary EPHF would be Public Health Surveillance and Monitoring (EPHF 1).
2. **Secondary EPHF**: If multiple EPHFs are relevant in the paper, list *all* all additional EPHFs. For example, if a study introduces a chatbot that educates a specific group about a health issue, it may contribute to both Health Promotion (EPHF 7) and Health Service Quality and Equity (EPHF 10). If Health Promotion is identified as the primary EPHF, then list Health Service Quality and Equity as the secondary EPHF here. If more than two EPHFs are relevant, mention all additional EPHFs in this section.
3. **Target Users**: List *all* the target users that the introduced NLP technologies serve. Please summarise the target users with the following categories:
   - **Healthcare Providers**: Direct care providers including doctors, nurses, practitioners, community health workers, and other healthcare professionals.
   - **Public Health Officials and Policymakers**: Individuals involved in public health policy, administration, and epidemiology.
   - **Researchers and Data Scientists**: Academics and professionals focused on public health research and data analysis.
   - **Specific Equity-Seeking Groups**: Populations grouped by protected demographic characteristics, such as people with disabilities, children, LGBTQ+ individuals, and the elderly, who advocate for health equity within and beyond their group.
   - **General Public**: The broader community, especially those at higher risk or in need of specific health interventions.
   - **Others**: Any target users that do not fit into the above categories.

   For example, if a paper introduces an NLP system that assists doctors in making diagnoses, the target users would be categorised as Healthcare Providers.
4. **Public Health Challenge**: Specific the public health challenges addressed by the NLP application, such as mental health, disease surveillance, maternal health, and vaccine hesitancy.
5. **Public Health Outcomes**: Document the measurable impact of the NLP technologies on public health outcomes as introduced in the study, such as any cost-effectiveness analysis. Specify if and how these health impacts were evaluated, such as through assessments of changes in health behaviours following the deployment of the technology in target populations. If no evaluation of health outcomes was performed, leave this entry blank.

[28] `https://www.who.int/teams/primary-health-care/health-systems-resilience/essential-public-health-functions`

6. **Levels of Impact and Engagement**: Categorise research papers based on their impact on, and levels of engagement with, public health in Africa. The categories range from direct interventions within African countries to contributions to global health discussions using African data. Select *one* of the following:

   - **Direct Impact on Public Health in Africa**: NLP technologies specifically designed to solve public health issues in African countries. These systems are often deployed and operational at national, sub-national, and local levels, directly addressing health challenges faced by local communities. This category also includes analyses using NLP tools that have informed or led to public health policies or interventions in African countries.
   - **Global Public Health Promotion with Indirect Impacts in Africa**: Systems that enhance public health on a global scale, indirectly contributing to the improvement of public health in Africa. These systems may not be exclusively focused on Africa but have global health implications that benefit African countries as well. This category also includes analyses using NLP tools that have informed or led to global public health policies or interventions relevant to Africa.
   - **Systems or Analyses Developed with African Public Health Data**: Systems and analyses that utilise data from African countries to develop technologies or insights. These may include social media data, health records, or public health data.
   - **Global Health Discussions with Examples from Africa**: Papers that contribute to broader global health discussions using examples or data from Africa.
   - **Others**: Any papers that do not fit into the above categories, with details provided.

   For example, if a paper introduces a chatbot to educate a specific group about a health issue, but the system has not yet been deployed, it would not fall into the first two categories. If the training data of the system includes data specific to African contexts, then the paper should be categorised under "Systems or Analyses Developed with African Data".

7. **Public Health Infrastructure Integration**: Indicate "True" if the NLP technology was integrated with existing public health infrastructure to align with current health operations, or "False" if it was not.

8. **Interoperability**: Indicate "True" if the NLP technology was designed to be interoperable with existing health information systems, facilitating seamless data exchange and communication, or "False" if it was not.

6.5 SDGs Coverage

1. **SDGs**: List the Sustainable Development Goals (SDGs)[29] targeted by the study. If multiple goals are addressed, list *all* relevant SDGs. For example, if a study introduces NLP techniques that minimise performance disparities between medical chatbots in different languages, it could contribute to both Good Health and Well-being (SDG 3) and Reduced Inequalities (SDG 10).

2. **SDG 3 Target**: Indicate the specific targets of SDG 3[30] addressed by the study. If multiple targets are covered, list *all* relevant targets. If no targets are covered, leave the entry blank. For example, if an NLP system provides the public with vaccine information, it could contribute to the SDG 3 target of research, development, and access to vaccines and medicines.

6.6 Timeliness and Deployment

1. **Year of Publication**: Record the year in which the paper was published. For example, if a paper was published in 2024 and uses data collected in 2020, write down 2024.

2. **Year of Data Collection**: Record the year in which the data used in the paper was collected. For papers introducing a dataset, unless otherwise specified, assume the year of data collection is the same as the year of publication. For papers that introduce an NLP system or perform an analysis using a dataset from other sources, the year of data collection is the year when the data was originally published. For example, if a paper was published in 2024 and uses data collected in 2020, write down 2020.

[29] `https://sdgs.un.org/goals`

[30] `https://sdgs.un.org/goals/goal3#targets_and_indicators`

3. **Deployment Stage**: Detail the current stage of development for each NLP technology reviewed. Select *one* from the following:
    - **Conceptualisation**: This initial stage is when the need for an NLP application is identified and its feasibility is considered. Position papers and extended abstracts fall into this category.
    - **Design and Prototyping**: Development of initial prototypes. These prototypes are usually evaluated based on their technical performance. Most research papers are in this category.
    - **Validation**: Rigorous testing of the system with public health outcomes to validate its effectiveness and efficiency in real-world settings. This includes work with rigorous field testing, such as randomised controlled trials (RCTs).
    - **Deployed and Operational**: Deployment of the NLP technology in actual public health settings, where it is actively used.
    - **Not Applicable**: The study does not introduce or utilise any new NLP technologies.

    For example, if a paper introduces a chatbot for health education, it can be classified into any of the above categories. If the introduced system has been implemented and tested with standard NLP evaluation metrics, such as accuracy, but not with public health outcomes, it should be classified as Design and Prototyping. If the system has been tested with public health outcomes but is not yet operational, it should be classified as Validation.

4. **Level of Accessibility**: Detail the level of accessibility of the NLP technologies introduced in the paper. This includes how wide a range of people could access the technology and whether the service is open-sourced or not. Select *one* from the following categories:
    - **Open-Source**: Publicly accessible datasets and tools that are open-source for future research and analysis.
    - **Publicly Available**: Datasets and NLP applications that are accessible to the general public via web or mobile but not necessarily open-source.
    - **Limited Access**: Datasets and NLP applications available only to certain users or under specific conditions.
        - Datasets that are available to specific groups of professional users but not accessible to the general public
        - NLP applications that can only be accessed by specific groups of professional users, for example, clinical decision support systems
    - **Closed-Access**: Datasets and applications that are not openly accessible outside the group of authors but may be available upon request or through collaboration.
        - Restricted datasets that are not shared outside the authors of the papers
        - NLP applications without access outside the research group
    - **Accessibility Irrelevant**: Papers where the focus is not on introducing or providing any specific resources.
        - Survey papers
        - Review papers
        - Theoretical papers
        - Position papers

    For example, if a paper introduces a medical report generation system for doctors, it can be classified as Limited Access if this system is not Open-Source. However, if the system is Open-Source, it should be documented as Open-Sourced.

5. **Available Platform**: Classify the platform on which the NLP technology is available based on the following categories:
    - **Mobile Applications**: Technologies accessible via mobile apps.
    - **Web-Based Applications**: Technologies accessible via web applications or online platforms.
    - **Web Services**: Technologies accessible via web-based Application Programming Interfaces (APIs) without user interfaces.
    - **Datasets**: Specific datasets used or provided in the study.
    - **NLP Tools and Libraries**: Specific NLP tools and libraries. These tools usually require installations on each deployed computer, which require expertise in computer science.
    - **Not Applicable**: The study does not introduce or utilise any new NLP technologies.

The key issue here is to understand to what extend a NLP system requires computer science expertise to use. For example, if a paper introduces a medical chatbot, and this systems only provide the source code for the system, it requires computer expertise to install this system, therefore is classified as NLP Tools and Libraries.

6.7 Ethical Considerations and User Engagement

1. **Ethical Approval**: Indicate "True" if the study received oversight from an institutional review board or equivalent ethics committee, or "False" if it did not.
2. **Data Privacy Compliance**: Indicate "True" if the study adhered to relevant data privacy regulations (e.g., GDPR, HIPAA) to ensure the protection of personal and sensitive data, or "False" if it did not.
3. **Informed Consent**: Indicate "True" if explicit consent was obtained from human participants after they were informed about the intended use of their data, or "False" if consent was not obtained.
4. **Community Involvement**: Indicate "True" if local communities were actively engaged in the design, implementation, or evaluation phases of the technology to ensure it meets specific population needs, or "False" if they were not.
5. **Stakeholder Collaboration**: Indicate "True" if the study includes documented collaboration with public health authorities, healthcare providers, or other relevant stakeholders to enhance the technology's effectiveness and acceptance, or "False" if it does not.
6. **Other Ethical Considerations**: Document any additional ethical considerations or practices described in the study, such as data anonymisation, measures to prevent misuse, or efforts to support vulnerable populations.

7 Categorisations of Included Studies

Below, we provide the complete list of citations for all categorisations referenced in this paper. This includes the 54 studies identified through our academic literature search [1, 2, 3, 4, 5, 6, 7, 8, 9, 10, 11, 12, 13, 14, 15, 16, 17, 18, 19, 20, 21, 22, 23, 24, 25, 26, 27, 28, 29, 30, 31, 32, 33, 34, 35, 36, 37, 38, 39, 40, 41, 42, 43, 44, 45, 46, 47, 48, 49, 50, 51, 52, 53, 54].

1. 38 papers contributed by authors affiliated with African institution [1, 6, 7, 8, 9, 10, 12, 14, 15, 16, 17, 18, 19, 20, 22, 23, 26, 27, 28, 30, 32, 34, 36, 38, 39, 40, 42, 43, 44, 45, 46, 47, 48, 49, 50, 51, 53, 54].
2. 35 authored by researchers affiliated with institutions outside Africa [2, 3, 4, 5, 6, 8, 9, 10, 11, 12, 13, 14, 16, 18, 20, 21, 22, 23, 24, 25, 29, 31, 33, 35, 37, 38, 40, 41, 42, 44, 46, 49, 51, 52, 54].
3. 19 papers stemmed from collaborations between African and non-African institutions [6, 8, 9, 10, 12, 14, 16, 18, 20, 22, 23, 38, 40, 42, 44, 46, 49, 51, 54].
4. 18 papers authored by researchers affiliated with institutions in the USA [3, 8, 12, 14, 18, 20, 21, 22, 23, 24, 29, 31, 38, 40, 41, 49, 51, 52].
5. 18 papers authored by researchers affiliated with institutions in the South Africa [10, 16, 17, 26, 27, 28, 30, 32, 38, 39, 40, 42, 43, 44, 45, 46, 48, 50].
6. 28 papers reported their funding source [2, 4, 5, 6, 8, 9, 10, 14, 16, 17, 18, 24, 26, 27, 30, 31, 32, 33, 34, 36, 37, 41, 45, 46, 47, 49, 51, 52].
7. 2 papers were funded by industry actors [6, 41].
8. 14 papers financially supported by institutions in North America [4, 6, 8, 10, 14, 16, 18, 24, 31, 32, 36, 46, 51, 52].
9. 9 papers financially supported by institutions in Europe [2, 9, 10, 16, 30, 33, 41, 46, 47].
10. 36 papers that reported the year of data collection [1, 2, 3, 4, 5, 6, 8, 9, 10, 11, 12, 13, 14, 16, 19, 21, 22, 24, 25, 26, 29, 30, 32, 33, 34, 36, 37, 38, 39, 40, 42, 43, 44, 46, 47, 48].
11. 13 papers used data collected in the same year [3, 6, 8, 9, 11, 12, 14, 19, 22, 24, 37, 38, 47].
12. 17 used data with a one-year delay [4, 5, 10, 16, 21, 25, 26, 29, 30, 32, 33, 34, 40, 42, 43, 44, 46].
13. 15 studies cover clinical domains [1, 8, 9, 13, 14, 22, 31, 39, 43, 48, 49, 50, 51, 52, 53].

14. 8 studies cover research domains [2, 4, 8, 11, 38, 39, 47, 54].
15. 36 studies cover general domains [3, 5, 6, 7, 10, 12, 15, 16, 17, 18, 19, 20, 21, 23, 24, 25, 26, 27, 28, 29, 30, 32, 33, 34, 35, 36, 37, 38, 40, 41, 42, 44, 45, 46, 47, 50].
16. 53 studies of text-based NLP technologies [1, 2, 3, 4, 5, 6, 7, 8, 9, 10, 11, 12, 13, 14, 15, 16, 18, 19, 20, 21, 22, 23, 24, 25, 26, 27, 28, 29, 30, 31, 32, 33, 34, 35, 36, 37, 38, 39, 40, 41, 42, 43, 44, 45, 46, 47, 48, 49, 50, 51, 52, 53, 54].
17. 2 studies of audio-based NLP technologies [17, 43].
18. 2 studies of image-based NLP technologies [14, 18].
19. 45 studies were designed to support the work of researchers and data scientists [1, 2, 4, 5, 6, 7, 8, 10, 11, 13, 14, 15, 16, 18, 19, 20, 21, 22, 23, 24, 25, 26, 27, 28, 29, 31, 32, 33, 34, 35, 36, 37, 38, 39, 40, 41, 42, 43, 44, 45, 46, 47, 48, 49, 54].
20. 38 studies were designed to support the work of public health officials and policymakers [3, 4, 5, 6, 7, 8, 10, 11, 14, 15, 16, 17, 18, 19, 21, 24, 25, 28, 29, 30, 31, 32, 33, 34, 35, 36, 37, 39, 40, 41, 42, 43, 44, 45, 46, 47, 48, 49].
21. 30 studies were designed to support the work of healthcare providers [1, 3, 7, 8, 9, 12, 13, 14, 15, 17, 18, 20, 22, 23, 26, 27, 30, 31, 32, 35, 39, 40, 43, 45, 47, 48, 49, 50, 51, 54].
22. 25 studies were public-facing [1, 3, 4, 7, 8, 12, 14, 15, 16, 17, 26, 27, 28, 30, 32, 33, 38, 40, 42, 44, 50, 51, 52, 53, 54].
23. 45 studies were designed to support the work of specific equity-seeking groups [3, 7, 20, 22, 23, 38, 47, 49].
24. 40 studies that support English [1, 2, 4, 6, 8, 9, 10, 11, 12, 13, 14, 15, 16, 17, 18, 20, 22, 23, 25, 26, 29, 30, 31, 32, 33, 35, 36, 38, 39, 41, 42, 43, 44, 45, 46, 48, 49, 51, 52, 53].
25. 8 studies that support Arabic [3, 5, 19, 21, 25, 30, 34, 37].
26. 4 studies that support French [3, 30, 36, 47].
27. 7 studies that support Kiswahili [3, 6, 8, 12, 20, 22, 23].
28. 4 studies that support Zulu [6, 17, 26, 40].
29. 40 studies targeting South Africa [2, 3, 6, 10, 13, 16, 17, 25, 26, 28, 29, 30, 32, 35, 38, 39, 40, 41, 42, 43, 44, 45, 46, 48, 50].
30. 9 studies targeting South Nigeria [2, 3, 4, 6, 11, 16, 30, 44, 52].
31. 4 reviews of existing NLP technologies [30, 31, 33, 38]
32. 1 study that was fully deployed and operational [52].
33. 44 studies are in the design and prototyping phase [3, 4, 5, 6, 7, 9, 10, 11, 12, 13, 16, 17, 18, 19, 20, 21, 22, 23, 24, 25, 26, 27, 28, 29, 32, 34, 35, 36, 37, 39, 40, 41, 42, 43, 44, 45, 46, 47, 48, 49, 50, 51, 53, 54].
34. 5 studies are in the validation phase [1, 2, 8, 14, 15].
35. 12 studies are open source [4, 5, 6, 10, 11, 14, 19, 20, 39, 45, 46, 49].
36. 17 studies are publicly available [1, 3, 21, 24, 25, 27, 28, 29, 34, 36, 42, 44, 48, 51, 52, 53, 54].
37. 18 studies are limited access [2, 7, 8, 9, 12, 13, 15, 16, 17, 18, 32, 35, 37, 40, 41, 43, 47, 50].
38. 3 studies are closed access [22, 23, 26].
39. 29 studies that provide nlp tools and libraries [6, 10, 11, 13, 16, 18, 19, 20, 21, 22, 23, 24, 25, 27, 28, 29, 32, 36, 37, 39, 41, 42, 44, 45, 46, 48, 49, 26, 54].
40. 5 studies that provide datasets [4, 5, 14, 26, 54].
41. 4 studies that provide web services [17, 3, 7, 50].
42. 11 studies that provide mobile applications [17, 50, 52, 53, 8, 12, 43, 47, 1, 40, 51].
43. 9 studies that provide web-based applications [2, 9, 15, 34, 35, 8, 12, 43, 47].
44. Out of the 50 reviewed NLP technologies, 40 indicate an intent to integrate their solutions into existing public health systems [7, 8, 9, 10, 11, 12, 13, 14, 16, 17, 18, 19, 20, 21, 22, 23, 24, 25, 26, 27, 28, 29, 32, 34, 35, 36, 37, 39, 40, 41, 42, 43, 45, 46, 47, 48, 49, 51, 52, 53].

45. Out of the 50 reviewed NLP technologies, 41 are designed to be interoperable with various health infrastructures [3, 6, 7, 8, 9, 10, 11, 12, 13, 14, 15, 16, 17, 18, 19, 20, 21, 22, 23, 24, 25, 26, 27, 28, 29, 32, 34, 35, 36, 37, 39, 40, 41, 42, 45, 46, 47, 48, 49, 51, 52].
46. 22 studies focus on addressing public health challenges within African countries [1, 7, 8, 9, 12, 14, 15, 16, 17, 18, 20, 22, 26, 27, 28, 30, 40, 47, 51, 52, 53, 54].
47. 8 studies adopt a broader global health perspective and address public health challenges on a global scale [3, 13, 24, 25, 31, 35, 38, 42].
48. 18 studies advance NLP technologies for public health using African data [2, 4, 5, 6, 10, 11, 32, 33, 34, 36, 39, 43, 44, 45, 46, 48, 49, 50].
49. 6 studies contribute to global health discussions, with Africa serving as a case study or example [19, 21, 23, 29, 37, 41].
50. 51 studies reported technical performance using a variety of automatic evaluation metrics [1, 2, 3, 4, 5, 6, 7, 8, 9, 10, 11, 12, 13, 14, 15, 16, 17, 18, 19, 20, 21, 22, 23, 24, 25, 26, 27, 28, 29, 30, 31, 32, 33, 34, 35, 36, 37, 38, 39, 41, 42, 43, 44, 45, 46, 49, 50, 51, 52, 53, 54].
51. 11 studies reported evaluation results based on user experiences [1, 8, 9, 20, 22, 27, 38, 40, 47, 52, 54].
52. 8 studies attempted to evaluate these technologies using health-related measures [4, 10, 18, 30, 35, 40, 48, 52].

8 Companies, NGOs, Events, and Research Communities in Literature Search

This scoping review focuses on NLP technologies developed by various contributors, as reported in online media or conference proceedings, rather than focusing on the contributors themselves. Consequently, these startups and NGOs are not listed in the main paper. Instead, we provide a comprehensive list of startups, NGOs, and conferences identified during our grey literature search in this section. Additionally, we include research communities dedicated to Africa NLP that were identified through our searches in both academic and grey literature.

8.1 Startups and Established Companies

In our grey literature search, we identified eight startups that are contributing to NLP technologies for public health in Africa. They are SophieBot[31], Elsa Health[32], Jacaranda Health[33], Digital Umuganda[34], Intron Health[35], AOS Rwanda[36], Ada Health[37], and Arxia[38]. These companies together contribute 4 commercial products and 1 initiative. Further details can be found in Section 9.3.

8.2 Initiatives and NGOs

Our grey literature search identified four initiatives led by the following organisations: Aurum Institute Ghana[39], AfriMed-QA[40], Reach Digital Health[41], and Ada's Global Health Initiative[42].

[31] `https://www.sophiebot.ai`
[32] `https://www.elsa.health`
[33] `https://jacarandahealth.org`
[34] `https://digitalumuganda.com`
[35] `IntronHealth`
[36] `AOSRwanda`
[37] `https://about.ada.com`
[38] `https://www.arxia.com/home.html`
[39] `https://www.auruminstitute.org/what-we-do/aurum-international/aurum-institute-ghana`
[40] `https://afrimedqa.com`
[41] `https://www.reachdigitalhealth.org`
[42] `https://about.ada.com/global-health-initiative/`

8.3 Events and Conferences

Our search identified four relevant events: 4th International Conference on Public Health in Africa (CPHIA 2024)[43], 5th Workshop on African Natural Language Processing (AfricaNLP 2024)[44], NLP Summit: Healthcare 2025 (April)[45], and The 2023 Conference on Empirical Methods in Natural Language Processing (EMNLP 2023)[46]. Among these, the EMNLP 2023 conference was already included in our structured database search of the ACL Anthology.

8.4 Events and Conferences

Our search identified four relevant events: 4th International Conference on Public Health in Africa (CPHIA 2024)[47], 5th Workshop on African Natural Language Processing (AfricaNLP 2024)[48], NLP Summit: Healthcare 2025 (April)[49], and The 2023 Conference on Empirical Methods in Natural Language Processing (EMNLP 2023)[50]. Among these, the EMNLP 2023 conference was already included in our structured database search of the ACL Anthology.

8.5 African NLP Research Communities

Through our search, we identified several African NLP research communities and groups actively contributing to the development of NLP technologies in Africa. These communities play a crucial role in advancing NLP technologies across the continent and addressing Africa-specific challenges. Their work has directly supported the development of various NLP technologies included in this review. While not exhaustive, the following list includes some of the key communities identified: Ethio NLP[51], Ghana NLP[52], HausaNLP[53], KANLP[54], Lanfrica[55], Masakhane[56], Mbaza NLP[57], and Sisonkebiotik[58].

[43] https://cphia2024.com
[44] https://sites.google.com/view/africanlp2024/home
[45] https://www.nlpsummit.org/healthcare-2025/
[46] https://2023.emnlp.org
[47] https://cphia2024.com
[48] https://sites.google.com/view/africanlp2024/home
[49] https://www.nlpsummit.org/healthcare-2025/
[50] https://2023.emnlp.org
[51] https://ethionlp.github.io
[52] https://ghananlp.org
[53] https://hausanlp.github.io
[54] https://www.kanlp.org
[55] https://lanfrica.com
[56] https://www.masakhane.io
[57] https://github.com/MBAZA-NLP
[58] https://sisonkebiotik.africa

9 Data Extraction Results

In this section, we present the detailed data extraction results, which include findings from 54 academic papers identified in the formal literature search, additional studies from grey literature, as well as an overview of commercial products and initiatives based on our grey literature search.

9.1 Academic Literature

Wellness Buddy: An AI Mental Health Chatbot for Kenyan University Students [1]

1. **Origin of the Work**: Kenya
2. **Affiliations**: Strathmore University
3. **Types of Funding**: Not Mentioned
4. **Continents of Funders**: Not Mentioned
5. **Supported Languages**: English (eng)
6. **Target African Countries or Regions**: Kenya (KEN)
7. **NLP Application**: Conversational Assistant
8. **Evaluation Method**: Technical Performance, User Experience
9. **Technical Evaluation Metrics**: Model Accuracy
10. **System Performance**: Training Accuracy = 0.9432, Validation Accuracy = 0.1863
11. **Domain Coverage**: Clinical Domain
12. **Modality**: Text
13. **Primary EPHF**: EPHF 7
14. **Secondary EPHFs**: EPHF 6, EPHF 10, EPHF 5
15. **Target Users**: General Public, Healthcare Providers, Researchers and Data Scientists
16. **Public Health Challenge**: Mental Health (Anxiety, Depression, Stress)
17. **Public Health Outcomes**: Not Measured
18. **Levels of Impact and Engagement**: Direct Impact on Public Health in Africa
19. **Public Health Infrastructure Integration**: False
20. **Interoperability**: False
21. **SDGs**: SDG 3, SDG 4
22. **SDG 3 Target**: 3.4
23. **Year of Publication**: 2024
24. **Year of Data Collection**: 2022
25. **Deployment Stage**: Validation
26. **Level of Accessibility**: Publicly Available
27. **Available Platform**: Mobile Applications
28. **Ethical Approval**: False
29. **Data Privacy Compliance**: True
30. **Informed Consent**: False
31. **Community Involvement**:False
32. **Stakeholder Collaboration**: False
33. **Other Ethical Considerations**: None Mentioned

Monitoring Disease Outbreak Events on the Web Using Text-mining Approach and Domain Expert Knowledge [2]

1. **Origin of the Work**: France
2. **Affiliations**: French Ministry of Agriculture, Food and Forestry (DGAL), French Agricultural Research Centre for International Development (Cirad), SONGES Project (FEDER and Languedoc-Roussillon)
3. **Types of Funding**: NGO Funding, Government Funding, Research Grant
4. **Continents of Funders**: Europe
5. **Supported Languages**: English
6. **Target African Countries or Regions**: Algeria, Angola, Benin, Botswana, Burkina Faso, Burundi, Cabo Verde, Cameroon, Central African Republic, Chad, Comoros, Congo, Democratic Republic of the Congo, Côte d'Ivoire, Djibouti, Egypt, Equatorial Guinea, Eritrea, Eswatini, Ethiopia, Gabon, Gambia, Ghana, Guinea, Guinea-Bissau, Kenya, Lesotho, Liberia, Libya, Madagascar, Malawi, Mali, Mauritania, Mauritius, Morocco, Mozambique, Namibia, Niger, Nigeria, Rwanda, Sao Tome and Principe, Senegal, Seychelles, Sierra Leone, Somalia, South Africa, South Sudan, Sudan, Tanzania, Togo, Tunisia, Uganda, Zambia, Zimbabwe, Western Sahara
7. **NLP Application**: Outbreak Detection
8. **Evaluation Method**: Technical Performance
9. **Technical Evaluation Metrics**: Precision, Recall, F1-score
10. **System Performance**: Text Mining Results: HRP Group: Precision = 0.6, Recall = 0.99, F1 = 0.69; HRP + CRP Group: Precision = 0.65, Recall = 0.99, F1 = 0.75; HRP + CRP + ARP Group: Precision = 0.83, Recall = 0.99, F1 = 0.87
11. **Domain Coverage**: Biomedical Domain
12. **Modality**: Text
13. **Primary EPHF**: EPHF 1
14. **Secondary EPHF**: EPHF 11
15. **Target Users**: Researchers and Data Scientists
16. **Public Health Challenge**: Timely detection and monitoring of disease outbreak events
17. **Public Health Outcomes**: Not Measured
18. **Levels of Impact and Engagement**: Systems or analyses developed with African data
19. **Public Health Infrastructure Integration**: False
20. **Interoperability**: False
21. **SDGs**: SDG 3, SDG 15
22. **SDG 3 Target**: 3.d
23. **Year of Publication**: 2016
24. **Year of Data Collection**: 2011-2014
25. **Deployment Stage**: Validation
26. **Level of Accessibility**: Limited Access
27. **Available Platform**: Web-Based Applications
28. **Ethical Approval**: False
29. **Data Privacy Compliance**: False
30. **Informed Consent**: False
31. **Community Involvement**: False
32. **Stakeholder Collaboration**: False
33. **Other Ethical Considerations**: None Mentioned

TICO-19: the Translation Initiative for COvid-19 [3]

1. **Origin of the Work**: United States of America
2. **Affiliations**: Translators without Borders, Carnegie Mellon University, Johns Hopkins University, George Mason University, Amazon Web Services, Appen, Facebook, Google, Microsoft
3. **Types of Funding**: Not Mentioned
4. **Continents of Funders**: Not Mentioned
5. **Supported Languages**: Amharic, Arabic, Bengali, Chinese, Czech, Dutch, French, German, Gujarati, Hausa, Hindi, Igbo, Indonesian, Italian, Japanese, Kannada, Korean, Malayalam, Marathi, Nepali, Pushto, Persian, Polish, Portuguese, Panjabi, Russian, Spanish, Swahili, Tagalog, Tamil, Telugu, Thai, Turkish, Urdu
6. **Target African Countries or Regions**: Ethiopia, Democratic Republic of the Congo, South Sudan, Nigeria, Niger, Rwanda, Uganda, Kenya, Somalia, Eritrea, South Africa
7. **NLP Application**: Machine Translation
8. **Evaluation Method**: Technical Performance
9. **Technical Evaluation Metrics**: BLEU Scores, Error Annotation
10. **System Performance**: Not Provided
11. **Domain Coverage**: General Domain
12. **Modality**: Text
13. **Primary EPHF**: EPHF 7
14. **Secondary EPHFs**: EPHF 11, EPHF 5, EPHF 2
15. **Target Users**: Healthcare Providers, Specific Equity-Seeking Groups, General Public, Public Health Officials and Policymakers
16. **Public Health Challenge**: Accurate and Accessible COVID-19 Information
17. **Public Health Outcomes**: Not Measured
18. **Levels of Impact and Engagement**: Global Public Health Promotion with Indirect African Impact
19. **Public Health Infrastructure Integration**: True
20. **Interoperability**: True
21. **SDGs**: SDG 3, SDG 10
22. **SDG 3 Targets**: 3.8, 3.3
23. **Year of Publication**: 2020
24. **Year of Data Collection**: 2020
25. **Deployment Stage**: Design and Prototyping
26. **Level of Accessibility**: Publicly Available
27. **Available Platform**: Web Services
28. **Ethical Approval**: False
29. **Data Privacy Compliance**: False
30. **Informed Consent**: False
31. **Community Involvement**: False
32. **Stakeholder Collaboration**: False
33. **Other Ethical Considerations**: None Mentioned

COVID-19 Vaccine Misinformation in Middle Income Countries [4]

1. **Origin of the Work**: USA, Indonesia
2. **Affiliations**: Boston University, Center for Emerging Infectious Diseases Policy & Research (CEID), NSF grants
3. **Types of Funding**: Research Grant
4. **Continents of Funders**: North America
5. **Supported Languages**: English, Portuguese, Indonesian
6. **Target African Countries or Regions**: Nigeria
7. **NLP Application**: Misinformation Detection
8. **Evaluation Method**: Technical Performance, Health Research Measure
9. **Technical Evaluation Metrics**: F1-Score
10. **System Performance**: Macro F1-Score (XLM-RoBERTa+AUG model): 0.77–0.89 (highest = 0.8987 for Q4c); Tweets Collected: 5,825,281; Vaccine-Relevant Tweets: 1,282,104 (22%); COVID-19 Vaccine-Relevant Tweets: 1,162,016 (90.6%); COVID-19 Vaccine Misinformation Tweets: 302,818 (26.1%); English Tweets: 99.37%
11. **Domain Coverage**: Biomedical Domain
12. **Modality**: Text
13. **Primary EPHF**: EPHF 1
14. **Secondary EPHFs**: EPHF 7, EPHF 5
15. **Target Users**: Public Health Officials and Policymakers, Researchers and Data Scientists, General Public
16. **Public Health Challenge**: Spread of COVID-19 Vaccine Misinformation
17. **Public Health Outcomes**: Not Mentioned
18. **Levels of Impact and Engagement**: Systems or Analyses Developed with African Data
19. **Public Health Infrastructure Integration**: False
20. **Interoperability**: False
21. **SDGs**: SDG 3, SDG 10
22. **SDG 3 Targets**: 3.b, 3.d, 3.3
23. **Year of Publication**: 2023
24. **Year of Data Collection**: 2020-2022
25. **Deployment Stage**: Design and Prototyping
26. **Level of Accessibility**: Open-Sourced
27. **Available Platform**: Datasets
28. **Ethical Approval**: True
29. **Data Privacy Compliance**: True
30. **Informed Consent**: False
31. **Community Involvement**: False
32. **Stakeholder Collaboration**: True
33. **Other Ethical Considerations**: True

AraNPCC: The Arabic Newspaper COVID-19 Corpus [5]

1. **Origin of the Work**: Saudi Arabia
2. **Affiliations**: King Abdulaziz City for Science and Technology (KACST)
3. **Types of Funding**: Government Funding
4. **Continents of Funders**: Asia
5. **Supported Languages**: Arabic
6. **Target African Countries or Regions**: Algeria, Egypt, Sudan, Tunisia, Morocco
7. **NLP Application**: Sentiment Analysis
8. **Evaluation Method**: Technical Performance
9. **Technical Evaluation Metrics**: Pearson Correlation, Kendall's Tau, Spearman's Rho
10. **System Performance**:
    - Total Corpus: Newspapers = 6; Texts = 2,926,693; Tokens = 747,884,209; Pearson = 0.368; Kendall's Tau = 0.499; Spearman's Rho = 0.694
    - Country-Specific Results:
      - Morocco: Pearson = 0.216, Kendall's Tau = 0.495, Spearman's Rho = 0.695; Newspapers = 4, Texts = 268,827, Tokens = 101,124,149
      - Algeria: Pearson = 0.448, Kendall's Tau = 0.597, Spearman's Rho = 0.771; Newspapers = 11, Texts = 439,204, Tokens = 133,040,389
      - Tunisia: Pearson = 0.413, Kendall's Tau = 0.546, Spearman's Rho = 0.734; Newspapers = 10, Texts = 509,427, Tokens = 92,404,722
      - Sudan: Pearson = 0.335, Kendall's Tau = 0.577, Spearman's Rho = 0.734; Newspapers = 11, Texts = 178,461, Tokens = 58,500,490
11. **Domain Coverage**: General Domain
12. **Modality**: Text
13. **Primary EPHF**: EPHF 11
14. **Secondary EPHFs**: EPHF 1, EPHF 2
15. **Target Users**: Researchers and Data Scientists, Public Health Officials, Policymakers
16. **Public Health Challenge**: Need for reliable data to analyze COVID-19 information coverage in Arabic newspapers
17. **Public Health Outcomes**: Not Mentioned
18. **Levels of Impact and Engagement**: Systems or Analyses Developed with African Data
19. **Public Health Infrastructure Integration**: False
20. **Interoperability**: False
21. **SDGs**: SDG 3
22. **SDG 3 Targets**: 3.d, 3.3
23. **Year of Publication**: 2022
24. **Year of Data Collection**: 2019-2021
25. **Deployment Stage**: Design and Prototyping
26. **Level of Accessibility**: Open-Sourced
27. **Available Platform**: Datasets
28. **Ethical Approval**: False
29. **Data Privacy Compliance**: True
30. **Informed Consent**: False
31. **Community Involvement**: False
32. **Stakeholder Collaboration**: False
33. **Other Ethical Considerations**: True

Global Readiness of Language Technology for Healthcare: What would it Take to Combat the Next Pandemic? [6]

1. **Origin of the Work**: India, Kenya
2. **Affiliations**: Microsoft Research Labs, Microsoft Africa Research Institute
3. **Types of Funding**: Industry Funding
4. **Continents of Funders**: North America, Africa
5. **Supported Languages**: English, Chinese, Hindi, Korean, Bengali, Malay, Swahili, Hausa, Marathi, Amharic, Zulu, Assamese, Gujarati, Kikuyu, Somali, Sinhala
6. **Target African Countries or Regions**: Kenya, Nigeria, South Africa, Somalia, Ethiopia
7. **NLP Application**: Conversational Assistant
8. **Evaluation Method**: Technical Performance
9. **Technical Evaluation Metrics**: Accuracy, F1 Score
10. **System Performance**:
    - Swahili: Intent = 28.08%, Entity = 24.6%
    - Hausa: Intent = 40.97%, Quarantine Intent = 76%, Incubation Intent = 100%
    - Amharic: Intent = 43.06%, Quarantine Intent = 56%, Incubation Intent = 100%
    - Zulu: Intent = 30.56%, Quarantine Intent = 20%, Incubation Intent = 0%
    - Kikuyu: Intent = 97.60%
    - Somali: Intent = 40.56%
11. **Domain Coverage**: General Domain
12. **Modality**: Text
13. **Primary EPHF**: EPHF 11
14. **Secondary EPHF**: EPHF 1
15. **Target Users**: Researchers and Data Scientists, Public Health Officials and Policymakers
16. **Public Health Challenge**: Enhancing the global readiness of language technology to effectively combat future pandemics
17. **Public Health Outcomes**: Not Mentioned
18. **Levels of Impact and Engagement**: Systems or Analyses Developed with African Data
19. **Public Health Infrastructure Integration**: False
20. **Interoperability**: False
21. **SDGs**: SDG 3, SDG 9
22. **SDG 3 Target**: 3.d
23. **Year of Publication**: 2022
24. **Year of Data Collection**: 2022
25. **Deployment Stage**: Design and Prototyping
26. **Level of Accessibility**: Open-Sourced
27. **Available Platform**: NLP Tools and Libraries
28. **Ethical Approval**: False
29. **Data Privacy Compliance**: False
30. **Informed Consent**: False
31. **Community Involvement**: False
32. **Stakeholder Collaboration**: True
33. **Other Ethical Considerations**: True

LLMs: A Promising New Tool for Improving Healthcare in Low-Resource Nations [7]

1. **Origin of the Work**: Rwanda, Sierra Leone
2. **Affiliations**: Center for AI Policy and Innovation Ltd., Grand Challenges Initiative, Bill & Melinda Gates Foundation
3. **Types of Funding**: Not Mentioned
4. **Continents of Funders**: Not Mentioned
5. **Supported Languages**: Kinyarwanda
6. **Target African Countries or Regions**: Rwanda
7. **NLP Application**: Conversational Assistant
8. **Evaluation Method**: Technical Performance
9. **Technical Evaluation Metrics**: Accuracy, Quality, User Satisfaction
10. **System Performance**: Not Applicable
11. **Domain Coverage**: General Domain
12. **Modality**: Text
13. **Primary EPHF**: EPHF 7
14. **Secondary EPHF**: EPHF 10
15. **Target Users**: Healthcare Providers, Public Health Officials and Policymakers, Researchers and Data Scientists, General Public, Specific Equity-Seeking Groups
16. **Public Health Challenge**: Leveraging Large Language Models (LLMs) to enhance healthcare delivery and access in low-resource nations
17. **Public Health Outcomes**: Not Mentioned
18. **Levels of Impact and Engagement**: Direct Impact on Public Health in Africa
19. **Public Health Infrastructure Integration**: False
20. **Interoperability**: False
21. **SDGs**: SDG 3, SDG 9, SDG 10
22. **SDG 3 Target**: 3.d
23. **Year of Publication**: 2023
24. **Year of Data Collection**: Not Mentioned
25. **Deployment Stage**: Design and Prototyping
26. **Level of Accessibility**: Limited Access
27. **Available Platform**: Web Services
28. **Ethical Approval**: False
29. **Data Privacy Compliance**: False
30. **Informed Consent**: False
31. **Community Involvement**: False
32. **Stakeholder Collaboration**: True
33. **Other Ethical Considerations**: None Mentioned

Expanding Access to Depression Treatment in Kenya Through Automated Psychological Support: Protocol for a Single-Case Experimental Design Pilot Study [8]

1. **Origin of the Work**: United States of America, Kenya
2. **Affiliations**: Duke Global Health Institute, Jacaranda Health, X2 AI Inc., Moi Teaching and Referral Hospital, Africa Mental Health Research and Training Foundation
3. **Types of Funding**: Research Grant
4. **Continents of Funders**: North America
5. **Supported Languages**: English, Swahili
6. **Target African Countries or Regions**: Kenya
7. **NLP Application**: Conversational Assistant
8. **Evaluation Method**: Technical Performance, User Experience
9. **Technical Evaluation Metrics**: User Engagement, User Satisfaction
10. **System Performance**: Assessed via system logs, engagement rates, and qualitative interviews
11. **Domain Coverage**: Biomedical Domain, Clinical Domain
12. **Modality**: Text
13. **Primary EPHF**: EPHF 7
14. **Secondary EPHFs**: EPHF 6, EPHF 5
15. **Target Users**: Healthcare Providers, Public Health Officials and Policymakers, Researchers and Data Scientists, General Public
16. **Public Health Challenge**: Expanding access to prenatal depression treatment in Kenya by providing automated psychological support
17. **Public Health Outcomes**: Not Mentioned
18. **Levels of Impact and Engagement**: Direct Impact on Public Health in Africa
19. **Public Health Infrastructure Integration**: True
20. **Interoperability**: True
21. **SDGs**: SDG 3, SDG 5
22. **SDG 3 Targets**: 3.1, 3.2
23. **Year of Publication**: 2019
24. **Year of Data Collection**: 2019
25. **Deployment Stage**: Validation
26. **Level of Accessibility**: Limited Access
27. **Available Platform**: Mobile Applications, Web-Based Applications
28. **Ethical Approval**: True
29. **Data Privacy Compliance**: True
30. **Informed Consent**: True
31. **Community Involvement**: True
32. **Stakeholder Collaboration**: True
33. **Other Ethical Considerations**: True

Investigating the Potential for Clinical Decision Support in Sub-Saharan Africa With AFYA (Artificial Intelligence-Based Assessment of Health Symptoms in Tanzania): Protocol for a Prospective, Observational Pilot Study [9]

1. **Origin of the Work**: Germany, Tanzania
2. **Affiliations**: Ada Health GmbH, Muhimbili University of Health and Allied Sciences, Else Kröner Fresenius Center for Digital Health, University Hospital Carl Gustav Carus Dresden
3. **Types of Funding**: Research Grant
4. **Continents of Funders**: Europe
5. **Supported Languages**: English
6. **Target African Countries or Regions**: Tanzania
7. **NLP Application**: Conversational Assistant
8. **Evaluation Method**: Technical Performance, User Experience
9. **Technical Evaluation Metrics**: Accuracy, User Satisfaction
10. **System Performance**: Not Applicable
11. **Domain Coverage**: Clinical Domain
12. **Modality**: Text
13. **Primary EPHF**: EPHF 9
14. **Secondary EPHFs**: EPHF 10, EPHF 7
15. **Target Users**: Healthcare Providers
16. **Public Health Challenge**: Enhancing clinical decision-making in Sub-Saharan Africa
17. **Public Health Outcomes**: Not Mentioned
18. **Levels of Impact and Engagement**: Direct Impact on Public Health in Africa
19. **Public Health Infrastructure Integration**: True
20. **Interoperability**: True
21. **SDGs**: SDG 3, SDG 8
22. **SDG 3 Targets**: 3.8, 3.c
23. **Year of Publication**: 2022
24. **Year of Data Collection**: 2021-2022
25. **Deployment Stage**: Design and Prototyping
26. **Level of Accessibility**: Limited Access
27. **Available Platform**: Web-Based Applications
28. **Ethical Approval**: True
29. **Data Privacy Compliance**: True
30. **Informed Consent**: True
31. **Community Involvement**: False
32. **Stakeholder Collaboration**: True
33. **Other Ethical Considerations**: True

Public Sentiments Toward COVID-19 Vaccines in South African Cities: An Analysis of Twitter Posts [10]

1. **Origin of the Work**: South Africa, Canada, Iran
2. **Affiliations**: Africa-Canada Artificial Intelligence and Data Innovation Consortium (ACADIC); York University; Canada's International Development Research Centre (IDRC); Swedish International Development Cooperation Agency (SIDA)
3. **Types of Funding**: NGO Funding
4. **Continents of Funders**: North America, Europe
5. **Supported Languages**: English
6. **Target African Countries or Regions**: South Africa
7. **NLP Application**: Sentiment Analysis
8. **Evaluation Method**: Technical Performance, Health Research Measure
9. **Technical Evaluation Metrics**: Frequency, Intensity
10. **System Performance**: Naive Bayes: Accuracy = 68%, Precision = 66%, Recall = 62%, F1 = 63%, AUC = 79%; Logistic Regression: Accuracy = 75%, Precision = 74%, Recall = 70%, F1 = 72%, AUC = 88%; Support Vector Machine: Accuracy = 70%, Precision = 73%, Recall = 61%, F1 = 63%, AUC = 86%; Decision Tree: Accuracy = 62%, Precision = 58%, Recall = 56%, F1 = 57%, AUC = 67%; K-Nearest Neighbors: Accuracy = 56%, Precision = 56%, Recall = 40%, F1 = 37%, AUC = 62%
11. **Domain Coverage**: General Domain
12. **Modality**: Text
13. **Primary EPHF**: EPHF 1
14. **Secondary EPHFs**: EPHF 7, EPHF 5
15. **Target Users**: Researchers and Data Scientists, Public Health Officials and Policymakers
16. **Public Health Challenge**: Understanding and addressing public sentiments toward COVID-19 vaccines in South African cities
17. **Public Health Outcomes**: Correlation observed between vaccine-related discussions on Twitter and vaccination rates, suggesting that social media sentiment analysis can support public health strategy.
18. **Levels of Impact and Engagement**: Systems or Analyses Developed with African Data
19. **Public Health Infrastructure Integration**: False
20. **Interoperability**: False
21. **SDGs**: SDG 3
22. **SDG 3 Targets**: 3.b, 3.3
23. **Year of Publication**: 2022
24. **Year of Data Collection**: 2021
25. **Deployment Stage**: Design and Prototyping
26. **Level of Accessibility**: Open-Sourced
27. **Available Platform**: NLP Tools and Libraries
28. **Ethical Approval**: True
29. **Data Privacy Compliance**: True
30. **Informed Consent**: False
31. **Community Involvement**: False
32. **Stakeholder Collaboration**: True
33. **Other Ethical Considerations**: True

Detecting Factors Responsible for Diabetes Prevalence in Nigeria Using Social Media and Machine Learning [11]

1. **Origin of the Work**: Canada
2. **Affiliations**: Dalhousie University
3. **Types of Funding**: Not Mentioned
4. **Continents of Funders**: Not Mentioned
5. **Supported Languages**: English
6. **Target African Countries or Regions**: Nigeria
7. **NLP Applications/Tasks**: Text Classification
8. **Evaluation Method**: Technical Performance
9. **Technical Evaluation Metrics**: Accuracy, Precision, Recall, F1 Score
10. **System Performance**: Collected 371,996 posts from Nairaland's Health forum, filtered to 3,051 diabetes-related posts from 872 topics. Naïve Bayes model performance: Accuracy = 87.08%, Precision = 79.52%, Recall = 82.5%, F1 Score = 80.98%.
11. **Domain Coverage**: Biomedical Domain
12. **Modality**: Text
13. **Primary EPHF**: EPHF 11
14. **Secondary EPHFs**: EPHF 7, EPHF 5
15. **Target Users**: Public Health Officials and Policymakers, Researchers and Data Scientists
16. **Public Health Challenge**: Identifying factors contributing to the high prevalence of diabetes in Nigeria using social media data
17. **Public Health Outcomes**: Not Mentioned
18. **Levels of Impact and Engagement**: Systems or Analyses Developed with African Data
19. **Public Health Infrastructure Integration**: False
20. **Interoperability**: False
21. **SDGs**: SDG 3
22. **SDG 3 Target**: 3.4
23. **Year of Publication**: 2019
24. **Year of Data Collection**: 2019
25. **Deployment Stage**: Design and Prototyping
26. **Level of Accessibility**: Open-Sourced
27. **Available Platform**: NLP Tools and Libraries
28. **Ethical Approval**: False
29. **Data Privacy Compliance**: False
30. **Informed Consent**: False
31. **Community Involvement**: False
32. **Stakeholder Collaboration**: False
33. **Other Ethical Considerations**: True

Intent Recognition on Low-Resource Language Messages in a Health Marketplace Chatbot [12]

1. **Origin of the Work**: United States of America, Nigeria
2. **Affiliations**: University of Nigeria, Nivi Inc
3. **Types of Funding**: Not Mentioned
4. **Continents of Funders**: Not Mentioned
5. **Supported Languages**: English, Swahili
6. **Target African Countries or Regions**: Kenya
7. **NLP Application**: Information Extraction
8. **Evaluation Method**: Technical Performance
9. **Technical Evaluation Metrics**: F1 Score, Precision, Recall
10. **System Performance**:
    - mBERT: Precision = 0.70, Recall = 0.75, F1 = 0.72
    - Logistic Regression (baseline): Precision = 0.61, Recall = 0.63, F1 = 0.62
11. **Domain Coverage**: General Domain
12. **Modality**: Text
13. **Primary EPHF**: EPHF 10
14. **Secondary EPHF**: EPHF 9
15. **Target Users**: Healthcare Providers, General Public
16. **Public Health Challenge**: Improving the accuracy of intent recognition in health-related inquiries within a marketplace chatbot
17. **Public Health Outcomes**: Not Mentioned
18. **Levels of Impact and Engagement**: Direct Impact on Public Health in Africa
19. **Public Health Infrastructure Integration**: False
20. **Interoperability**: False
21. **SDGs**: SDG 3, SDG 10, SDG 8
22. **SDG 3 Targets**: 3.8, 3.c
23. **Year of Publication**: 2023
24. **Year of Data Collection**: 2023
25. **Deployment Stage**: Design and Prototyping
26. **Level of Accessibility**: Limited Access
27. **Available Platform**: Mobile Applications, Web-Based Applications
28. **Ethical Approval**: False
29. **Data Privacy Compliance**: True
30. **Informed Consent**: False
31. **Community Involvement**: False
32. **Stakeholder Collaboration**: False
33. **Other Ethical Considerations**: True

Disease Prediction Based on Individual's Medical History Using CNN [13]

1. **Origin of the Work**: Germany
2. **Affiliations**: South Westphalia University of Applied Sciences
3. **Types of Funding**: Not Mentioned
4. **Continents of Funders**: Not Mentioned
5. **Supported Languages**: English
6. **Target African Countries or Regions**: South Africa
7. **NLP Application**: Event Prediction
8. **Evaluation Method**: Technical Performance
9. **Technical Evaluation Metrics**: Accuracy, Precision, Recall, F1 Score
10. **System Performance**:
    - Accuracy = 80.73%
    - Precision = 0.81, Recall = 0.81, F1 Score = 0.80

    The study achieved high performance in predicting future disease risks based on historical data.
11. **Domain Coverage**: Clinical Domain
12. **Modality**: Text
13. **Primary EPHF**: EPHF 11
14. **Secondary EPHFs**: EPHF 9, EPHF 10
15. **Target Users**: Healthcare Providers, Researchers and Data Scientists
16. **Public Health Challenge**: Improving disease prediction accuracy
17. **Public Health Outcomes**: The model achieved 80.73% accuracy in predicting future disease risks, suggesting potential utility in early disease detection and intervention, which may improve health outcomes.
18. **Levels of Impact and Engagement**: Global Public Health Promotion with Indirect African Impact
19. **Public Health Infrastructure Integration**: False
20. **Interoperability**: False
21. **SDGs**: SDG 3, SDG 9, SDG 10
22. **SDG 3 Target**: 3.b
23. **Year of Publication**: 2021
24. **Year of Data Collection**: 2018-2019
25. **Deployment Stage**: Design and Prototyping
26. **Level of Accessibility**: Limited Access
27. **Available Platform**: NLP Tools and Libraries
28. **Ethical Approval**: False
29. **Data Privacy Compliance**: True
30. **Informed Consent**: False
31. **Community Involvement**: False
32. **Stakeholder Collaboration**: False
33. **Other Ethical Considerations**: True

Application of Machine Learning to Prediction of Surgical Site Infection [14]

1. **Origin of the Work**: United States of America, Rwanda
2. **Affiliations**: MIT, Harvard Medical School, Partners In Health (Rwanda)
3. **Types of Funding**: Government Funding
4. **Continents of Funders**: North America
5. **Supported Languages**: English
6. **Target African Countries or Regions**: Rwanda
7. **NLP Application**: Event Prediction
8. **Evaluation Method**: Technical Performance
9. **Technical Evaluation Metrics**: Accuracy, Sensitivity, Specificity, ROC AUC
10. **System Performance**:
    - **Logistic Regression (L1 Regularization)**: Accuracy = 85.29%, Sensitivity = 0.7134, Specificity = 0.9846, ROC AUC = 0.8529
    - **Logistic Regression (L2 Regularization)**: Accuracy = 86.66%, Sensitivity = 0.75, Specificity = 0.9921, ROC AUC = 0.8666
    - **SVM**: Accuracy = 84.90%, Sensitivity = 0.7059, Specificity = 1.0, ROC AUC = 0.8490
11. **Domain Coverage**: Clinical Domain
12. **Modality**: Text, Image
13. **Primary EPHF**: EPHF 11
14. **Secondary EPHF**: EPHF 6
15. **Target Users**: Healthcare Providers, Researchers and Data Scientists, Public Health Officials and Policymakers, General Public
16. **Public Health Challenge**: Reducing the incidence of surgical site infections through enhanced preventive measures and postoperative care
17. **Public Health Outcomes**: The study demonstrated high accuracy in predicting surgical site infections using questionnaire and image data, suggesting potential improvements in post-surgical care in low-resource settings.
18. **Levels of Impact and Engagement**: Direct Impact on Public Health in Africa
19. **Public Health Infrastructure Integration**: False
20. **Interoperability**: False
21. **SDGs**: SDG 3, SDG 9
22. **SDG 3 Target**: 3.1
23. **Year of Publication**: 2017
24. **Year of Data Collection**: 2017
25. **Deployment Stage**: Validation
26. **Level of Accessibility**: Open-Sourced
27. **Available Platform**: Datasets
28. **Ethical Approval**: False
29. **Data Privacy Compliance**: False
30. **Informed Consent**: False
31. **Community Involvement**: True
32. **Stakeholder Collaboration**: True
33. **Other Ethical Considerations**: True

A Deep Learning-Based Chatbot to Enhance Maternal Health Education [15]

1. **Origin of the Work**: Lesotho, Eswatini
2. **Affiliations**: Botho University, University of Eswatini
3. **Types of Funding**: Not Mentioned
4. **Continents of Funders**: Not Mentioned
5. **Supported Languages**: English, Sotho, Shona, North Ndebele
6. **Target African Countries or Regions**: Lesotho, Zimbabwe
7. **NLP Application**: Conversational Assistant
8. **Evaluation Method**: Technical Performance
9. **Technical Evaluation Metrics**: Accuracy, Loss
10. **System Performance**:
    - Training Parameters: 1000 epochs, batch size = 8, learning rate = 0.001, hidden size = 8
    - Results: Training and Evaluation Losses approached zero; Training and Evaluation Accuracies reached 100%
11. **Domain Coverage**: General Domain
12. **Modality**: Text
13. **Primary EPHF**: EPHF 7
14. **Secondary EPHF**: EPHF 10
15. **Target Users**: Healthcare Providers, General Public, Researchers and Data Scientists, Public Health Officials and Policymakers
16. **Public Health Challenge**: Improving maternal health education
17. **Public Health Outcomes**: The chatbot achieved 100% accuracy in training and evaluation, indicating potential effectiveness for maternal health education. However, the full impact on health outcomes is yet to be evaluated in real-world settings.
18. **Levels of Impact and Engagement**: Direct Impact on Public Health in Africa
19. **Public Health Infrastructure Integration**: False
20. **Interoperability**: True
21. **SDGs**: SDG 3
22. **SDG 3 Targets**: 3.1, 3.2
23. **Year of Publication**: 2024
24. **Year of Data Collection**: Not Mentioned
25. **Deployment Stage**: Validation
26. **Level of Accessibility**: Limited Access
27. **Available Platform**: Web-Based Applications
28. **Ethical Approval**: False
29. **Data Privacy Compliance**: Not Mentioned
30. **Informed Consent**: False
31. **Community Involvement**: False
32. **Stakeholder Collaboration**: True
33. **Other Ethical Considerations**: True

Vaccine Hesitancy Hotspots in Africa: An Insight From Geotagged Twitter Posts [16]

1. **Origin of the Work**: Canada, Iran, South Africa
2. **Affiliations**: Canada's International Development Research Center (IDRC), Swedish International Development Cooperation Agency (SIDA), NSERC, New Frontier in Research Fund-Exploratory
3. **Types of Funding**: NGO Funding
4. **Continents of Funders**: North America, Europe
5. **Supported Languages**: English
6. **Target African Countries or Regions**: Nigeria, South Africa, Zimbabwe, Botswana, Namibia, Rwanda, Mozambique, Cameroon, Eswatini
7. **NLP Application**: Sentiment Analysis
8. **Evaluation Method**: Technical Performance
9. **Technical Evaluation Metrics**: Accuracy, Average AUC
10. **System Performance**:
    - Dataset: 70,000 geotagged vaccine-related tweets from nine African countries
    - Annotation Tool: VADER sentiment analysis, validated with machine learning classifiers
    - Logistic Regression (LR): Highest performance with 71% accuracy and 85% AUC
    - Model Accuracy: LR 71%, NB 66%, SVMs 65%, DT 61%, KNN 56%
    - Average AUC: LR 85%, SVMs 83%, NB 78%, DT 67%, KNN 63%
11. **Domain Coverage**: General Domain
12. **Modality**: Text
13. **Primary EPHF**: EPHF 1
14. **Secondary EPHF**: EPHF 7
15. **Target Users**: Public Health Officials and Policymakers, Researchers and Data Scientists, General Public
16. **Public Health Challenge**: Identifying and understanding vaccine hesitancy hotspots in Africa
17. **Public Health Outcomes**: Not Mentioned
18. **Levels of Impact and Engagement**: Direct Impact on Public Health in Africa
19. **Public Health Infrastructure Integration**: False
20. **Interoperability**: False
21. **SDGs**: SDG 3, SDG 9
22. **SDG 3 Target**: 3.b
23. **Year of Publication**: 2023
24. **Year of Data Collection**: 2020-2022
25. **Deployment Stage**: Design and Prototyping
26. **Level of Accessibility**: Limited Access
27. **Available Platform**: NLP Tools and Libraries
28. **Ethical Approval**: False
29. **Data Privacy Compliance**: True
30. **Informed Consent**: False
31. **Community Involvement**: False
32. **Stakeholder Collaboration**: True
33. **Other Ethical Considerations**: True

AwezaMed: A Multilingual, Multimodal Speech-To-Speech Translation Application for Maternal Health Care [17]

1. **Origin of the Work**: South Africa
2. **Affiliations**: CSIR
3. **Types of Funding**: Government Funding
4. **Continents of Funders**: Africa
5. **Supported Languages**: English, Afrikaans, Xhosa, Zulu
6. **Target African Countries or Regions**: South Africa
7. **NLP Application**: Machine Translation
8. **Evaluation Method**: Technical Performance
9. **Technical Evaluation Metrics**: Error Rate, User Feedback
10. **System Performance**:
    - Performance Metrics (across three environments):
      - Site A: PER = 7.5%-10%, WER = 5%-10%, PS = 80%-90%
      - Site B: PER = 20%-55%, WER = 5%-35%, PS = 100%
      - Site C: PER = 0%-30%, WER = 5%-20%, PS = 75%-95%
    - Lower PERs observed in controlled environments
    - Positive user feedback indicating effective communication and improved respect for patients
11. **Domain Coverage**: General Domain
12. **Modality**: Audio
13. **Primary EPHF**: EPHF 10
14. **Secondary EPHF**: EPHF 7
15. **Target Users**: Healthcare Providers, General Public, Public Health Officials, Policymakers
16. **Public Health Challenge**: Overcoming language barriers in maternal healthcare
17. **Public Health Outcomes**: Pilot feedback suggests AwezaMed enhances communication, leading to better patient care and satisfaction, especially in multilingual, low-resource settings.
18. **Levels of Impact and Engagement**: Direct Impact on Public Health in Africa
19. **Public Health Infrastructure Integration**: False
20. **Interoperability**: True
21. **SDGs**: SDG 3, SDG 10
22. **SDG 3 Targets**: 3.1, 3.2
23. **Year of Publication**: 2020
24. **Year of Data Collection**: Not Mentioned
25. **Deployment Stage**: Design and Prototyping
26. **Level of Accessibility**: Limited Access
27. **Available Platform**: Mobile Applications, Web Services
28. **Ethical Approval**: False
29. **Data Privacy Compliance**: False
30. **Informed Consent**: False
31. **Community Involvement**: False
32. **Stakeholder Collaboration**: True
33. **Other Ethical Considerations**: True

Handwritten Text and Digit Classification on Rwandan Perioperative Flowsheets via YOLOv5 [18]

1. **Origin of the Work**: Rwanda, United States of America
2. **Affiliations**: Center for Global Inquiry & Innovation at the University of Virginia, Research Computing at UVA
3. **Types of Funding**: Research Grant
4. **Continents of Funders**: North America
5. **Supported Languages**: English
6. **Target African Countries or Regions**: Rwanda
7. **NLP Application**: Optical Character Recognition
8. **Evaluation Method**: Technical Performance, Health Research Measure
9. **Technical Evaluation Metrics**: Mean Average Precision (mAP)
10. **System Performance**:
    - Single Class mAP[0.5]: Physiological Indicators = 63.56%, Drugs Section = 98.43%
    - Multi-Class mAP[0.5]: Physiological Indicators = 12.64%, Drugs Section = 35.95%
    - High mAP observed for single-class models, indicating effective object detection; lower performance in multi-class models due to class imbalance and variability in handwritten data.
11. **Domain Coverage**: General Domain
12. **Modality**: Text, Image
13. **Primary EPHF**: EPHF 10
14. **Secondary EPHF**: EPHF 11
15. **Target Users**: Healthcare Providers, Researchers and Data Scientists, Public Health Officials and Policymakers
16. **Public Health Challenge**: Enhancing the accuracy and efficiency of data extraction from handwritten perioperative flowsheets using YOLOv5 for text and digit classification to improve patient record management and surgical care
17. **Public Health Outcomes**: The study suggests that YOLOv5 can significantly improve the accuracy and timeliness of perioperative data recording, enhancing patient outcomes and healthcare quality.
18. **Levels of Impact and Engagement**: Direct Impact on Public Health in Africa
19. **Public Health Infrastructure Integration**: False
20. **Interoperability**: True
21. **SDGs**: SDG 3, SDG 9
22. **SDG 3 Target**: 3.d
23. **Year of Publication**: 2022
24. **Year of Data Collection**: Not Mentioned
25. **Deployment Stage**: Design and Prototyping
26. **Level of Accessibility**: Limited Access
27. **Available Platform**: NLP Tools and Libraries
28. **Ethical Approval**: False
29. **Data Privacy Compliance**: False
30. **Informed Consent**: False
31. **Community Involvement**: False
32. **Stakeholder Collaboration**: True
33. **Other Ethical Considerations**: True

AraDepSu: Detecting Depression and Suicidal Ideation in Arabic Tweets Using Transformers [19]

1. **Origin of the Work**: Egypt
2. **Affiliations**: Faculty of Engineering, Alexandria University, Egypt
3. **Types of Funding**: Not Mentioned
4. **Continents of Funders**: Not Mentioned
5. **Supported Languages**: Arabic
6. **Target African Countries or Regions**: Egypt
7. **NLP Application**: Sentiment Analysis
8. **Evaluation Method**: Technical Performance
9. **Technical Evaluation Metrics**: Accuracy, Precision, Recall, F1 Score
10. **System Performance**: MARBERT model achieved the highest performance:
    - Accuracy = 91.20%
    - Macro-Average Precision = 88.74%
    - Macro-Average Recall = 88.50%
    - Macro-Average F1 Score = 88.75%
11. **Domain Coverage**: General Domain
12. **Modality**: Text
13. **Primary EPHF**: EPHF 11
14. **Secondary EPHFs**: EPHF 1, EPHF 5
15. **Target Users**: Public Health Officials and Policymakers, Researchers and Data Scientists
16. **Public Health Challenge**: Detecting depression and suicidal ideation among Arabic-speaking populations
17. **Public Health Outcomes**: The study shows that transformer models like MARBERT can effectively detect depression and suicidal ideation in Arabic tweets, potentially enabling earlier interventions and improved mental health outcomes.
18. **Levels of Impact and Engagement**: Global Health Discussions with African Examples
19. **Public Health Infrastructure Integration**: False
20. **Interoperability**: False
21. **SDGs**: SDG 3
22. **SDG 3 Target**: 3.4
23. **Year of Publication**: 2022
24. **Year of Data Collection**: 2016-2022
25. **Deployment Stage**: Design and Prototyping
26. **Level of Accessibility**: Open-Sourced
27. **Available Platform**: NLP Tools and Libraries
28. **Ethical Approval**: False
29. **Data Privacy Compliance**: False
30. **Informed Consent**: False
31. **Community Involvement**: False
32. **Stakeholder Collaboration**: True
33. **Other Ethical Considerations**: None Mentioned

Language Patterns and Behaviour of the Peer Supporters in Multilingual Healthcare Conversational Forums [20]

1. **Origin of the Work**: India, Kenya, United States of America
2. **Affiliations**: Microsoft Research India, University of Washington, Microsoft Africa Research Institute, Kenya
3. **Types of Funding**: Not Mentioned
4. **Continents of Funders**: Not Mentioned
5. **Supported Languages**: English, Swahili
6. **Target African Countries or Regions**: Kenya
7. **NLP Application**: Sentiment Analysis
8. **Evaluation Method**: Technical Performance, User Experience
9. **Technical Evaluation Metrics**: F1 Score, Precision, Recall
10. **System Performance**:
    - Few-shot XLM-R: Precision = 0.17, Recall = 0.53, F1 = 0.26; Few-shot mBERT: Precision = 0.16, Recall = 0.48, F1 = 0.24
    - Dataset: WhatsApp chat logs from two peer-support groups for Kenyan youth living with HIV, totaling 1,655 messages in Group-1 (28 members) and 4,901 messages in Group-2 (27 members)
    - Language Usage: Predominantly English with frequent Kiswahili and Sheng, significant code-mixing observed
11. **Domain Coverage**: General Domain
12. **Modality**: Text
13. **Primary EPHF**: EPHF 7
14. **Secondary EPHFs**: EPHF 11, EPHF 5, EPHF 2
15. **Target Users**: Specific Equity-Seeking Groups, Healthcare Providers, Researchers and Data Scientists
16. **Public Health Challenge**: Understanding language patterns and behaviors of peer supporters for mental health in multilingual healthcare conversational forums
17. **Public Health Outcomes**: Not Mentioned
18. **Levels of Impact and Engagement**: Direct Impact on Public Health in Africa
19. **Public Health Infrastructure Integration**: False
20. **Interoperability**: False
21. **SDGs**: SDG 3, SDG 10
22. **SDG 3 Target**: 3.4
23. **Year of Publication**: 2022
24. **Year of Data Collection**: Not Mentioned
25. **Deployment Stage**: Design and Prototyping
26. **Level of Accessibility**: Open-Sourced
27. **Available Platform**: NLP Tools and Libraries
28. **Ethical Approval**: False
29. **Data Privacy Compliance**: True
30. **Informed Consent**: False
31. **Community Involvement**: True
32. **Stakeholder Collaboration**: False
33. **Other Ethical Considerations**: True

ArCovidVac: Analyzing Arabic Tweets About COVID-19 Vaccination [21]

1. **Origin of the Work**: Qatar, United States of America
2. **Affiliations**: Qatar Computing Research Institute (QCRI), Hamad Bin Khalifa University, University of Pittsburgh
3. **Types of Funding**: Not Mentioned
4. **Continents of Funders**: Not Mentioned
5. **Supported Languages**: Arabic
6. **Target African Countries or Regions**: Egypt
7. **NLP Application**: Sentiment Analysis
8. **Evaluation Method**: Technical Performance
9. **Technical Evaluation Metrics**: Annotation Agreement, Accuracy, F1 Score
10. **System Performance**:
    - Egypt: Total Tweets Analyzed = 1,200; Sentiment Distribution: Positive = 650, Negative = 350, Neutral = 200; Performance Metrics: Cohen's Kappa = 0.80, Accuracy = 83%, F1 Score = 0.81; Findings: High positivity linked to vaccine availability and government campaigns; negativity tied to side effects and misinformation.
    - Morocco: Total Tweets Analyzed = 1,050; Sentiment Distribution: Positive = 600, Negative = 300, Neutral = 150; Performance Metrics: Cohen's Kappa = 0.78, Accuracy = 81%, F1 Score = 0.79; Findings: Positive sentiment on vaccination success and celebrity endorsements; negativity due to efficacy concerns and distribution issues.
11. **Domain Coverage**: General Domain
12. **Modality**: Text
13. **Primary EPHF**: EPHF 1
14. **Secondary EPHFs**: EPHF 7, EPHF 2
15. **Target Users**: Public Health Officials and Policymakers, Researchers and Data Scientists
16. **Public Health Challenge**: Understanding public perceptions and attitudes towards COVID-19 vaccination among Arabic-speaking populations
17. **Public Health Outcomes**: Not Mentioned
18. **Levels of Impact and Engagement**: Global Health Discussions with African Examples
19. **Public Health Infrastructure Integration**: False
20. **Interoperability**: True
21. **SDGs**: SDG 3
22. **SDG 3 Targets**: 3.b, 3.3
23. **Year of Publication**: 2022
24. **Year of Data Collection**: 2021
25. **Deployment Stage**: Design and Prototyping
26. **Level of Accessibility**: Publicly Available
27. **Available Platform**: NLP Tools and Libraries
28. **Ethical Approval**: False
29. **Data Privacy Compliance**: True
30. **Informed Consent**: False
31. **Community Involvement**: False
32. **Stakeholder Collaboration**: True
33. **Other Ethical Considerations**: None Mentioned

Detecting Urgency in Multilingual Medical SMS in Kenya [22]

1. **Origin of the Work**: United States of America, Kenya
2. **Affiliations**: University of Washington, Jomo Kenyatta University of Agriculture & Technology
3. **Types of Funding**: Not Mentioned
4. **Continents of Funders**: Not Mentioned
5. **Supported Languages**: English, Swahili, Luo
6. **Target African Countries or Regions**: Kenya
7. **NLP Application**: Sentiment Analysis
8. **Evaluation Method**: Technical Performance, User Experience
9. **Technical Evaluation Metrics**: Precision, Recall, F1 Score
10. **System Performance**:
    - mBERT with nurse context: Precision = 52, Recall = 38, F1 = 44
11. **Domain Coverage**: Clinical Domain
12. **Modality**: Text
13. **Primary EPHF**: EPHF 7
14. **Secondary EPHFs**: EPHF 9, EPHF 10
15. **Target Users**: Healthcare Providers, Researchers and Data Scientists, Specific Equity-Seeking Groups
16. **Public Health Challenge**: Improving the response to urgent medical needs by detecting the urgency of multilingual medical SMS messages
17. **Public Health Outcomes**: Not Mentioned
18. **Levels of Impact and Engagement**: Direct Impact on Public Health in Africa
19. **Public Health Infrastructure Integration**: True
20. **Interoperability**: True
21. **SDGs**: SDG 3, SDG 8
22. **SDG 3 Targets**: 3.c, 3.1, 3.2
23. **Year of Publication**: 2022
24. **Year of Data Collection**: 2017-2022
25. **Deployment Stage**: Design and Prototyping
26. **Level of Accessibility**: Closed-Access
27. **Available Platform**: NLP Tools and Libraries
28. **Ethical Approval**: True
29. **Data Privacy Compliance**: True
30. **Informed Consent**: True
31. **Community Involvement**: True
32. **Stakeholder Collaboration**: True
33. **Other Ethical Considerations**: True

A Linguistic Annotation Framework to Study Interactions in Multilingual Healthcare Conversational Forums [23]

1. **Origin of the Work**: India, Kenya, United States of America
2. **Affiliations**: Microsoft Research Labs, Microsoft Africa Research Institute (MARI), Paul G. Allen School of Computer Science and Engineering, University of Washington
3. **Types of Funding**: Not Mentioned
4. **Continents of Funders**: Not Mentioned
5. **Supported Languages**: English, Swahili
6. **Target African Countries or Regions**: Kenya
7. **NLP Application**: Sentiment Analysis
8. **Evaluation Method**: Technical Performance
9. **Technical Evaluation Metrics**: Annotation Agreement, Sentiment Tagging, Ethnographic Analysis
10. **System Performance**:
    - Inter-Annotator Agreement (Cohen's Kappa): English = 0.98, Swahili = 0.98, Sheng = 0.87, Code-Mixed Phrases = 0.85
    - Sentiment Labeling Agreement: Kappa = 0.89
    - Code-Mixing Prevalence: 23.8% in Group-1, 24.4% in Group-2
    - Sentiment Distribution: Majority Neutral, followed by Negative and Positive
11. **Domain Coverage**: General Domain
12. **Modality**: Text
13. **Primary EPHF**: EPHF 11
14. **Secondary EPHFs**: EPHF 7, EPHF 10, EPHF 2
15. **Target Users**: Researchers and Data Scientists, Healthcare Providers, Specific Equity-Seeking Groups
16. **Public Health Challenge**: Enhancing the understanding of patient-provider and peer-to-peer interactions for mental health in multilingual healthcare conversational forums
17. **Public Health Outcomes**: Not Mentioned
18. **Levels of Impact and Engagement**: Global Health Discussions with African Examples
19. **Public Health Infrastructure Integration**: False
20. **Interoperability**: True
21. **SDGs**: SDG 3, SDG 10
22. **SDG 3 Target**: 3.3
23. **Year of Publication**: 2021
24. **Year of Data Collection**: Not Mentioned
25. **Deployment Stage**: Design and Prototyping
26. **Level of Accessibility**: Closed-Access
27. **Available Platform**: NLP Tools and Libraries
28. **Ethical Approval**: False
29. **Data Privacy Compliance**: True
30. **Informed Consent**: False
31. **Community Involvement**: True
32. **Stakeholder Collaboration**: False
33. **Other Ethical Considerations**: True

Interactive Refinement of Cross-Lingual Word Embeddings [24]

1. **Origin of the Work**: United States of America
2. **Affiliations**: Office of the Director of National Intelligence (ODNI), Intelligence Advanced Research Projects Activity (IARPA), via the BETTER Program
3. **Types of Funding**: Government Funding
4. **Continents of Funders**: North America
5. **Supported Languages**: Iloko, Sinhala, Tigrinya, Uighur
6. **Target African Countries or Regions**: Ethiopia, Eritrea
7. **NLP Application**: Word Embedding
8. **Evaluation Method**: Technical Performance
9. **Technical Evaluation Metrics**: Accuracy
10. **System Performance**:
    - Base Model Accuracy: 55.9%
    - CLIME Refined Accuracy: 77.1%
    - CCA Embeddings: Accuracy improved from 55.9% to 77.1% using CLIME refinement
11. **Domain Coverage**: General Domain
12. **Modality**: Text
13. **Primary EPHF**: EPHF 11
14. **Secondary EPHF**: EPHF 1
15. **Target Users**: Public Health Officials and Policymakers, Researchers and Data Scientists
16. **Public Health Challenge**: General Health Information Communication
17. **Public Health Outcomes**: Not Mentioned
18. **Levels of Impact and Engagement**: Global Public Health Promotion with Indirect African Impact
19. **Public Health Infrastructure Integration**: False
20. **Interoperability**: True
21. **SDGs**: SDG 3, SDG 9
22. **SDG 3 Target**: 3.8
23. **Year of Publication**: 2020
24. **Year of Data Collection**: 2020
25. **Deployment Stage**: Design and Prototyping
26. **Level of Accessibility**: Publicly Available
27. **Available Platform**: NLP Tools and Libraries
28. **Ethical Approval**: False
29. **Data Privacy Compliance**: True
30. **Informed Consent**: False
31. **Community Involvement**: True
32. **Stakeholder Collaboration**: True
33. **Other Ethical Considerations**: True

A Language-independent Network to Analyze the Impact of COVID-19 on the World via Sentiment Analysis [25]

1. **Origin of the Work**: India
2. **Affiliations**: Delhi Technological University
3. **Types of Funding**: Not Mentioned
4. **Continents of Funders**: Not Mentioned
5. **Supported Languages**: Hindi, Japanese, Arabic, Spanish, Urdu, English
6. **Target African Countries or Regions**: South Africa
7. **NLP Application**: Sentiment Analysis
8. **Evaluation Method**: Technical Performance
9. **Technical Evaluation Metrics**: Accuracy, Precision, Recall, F1 Score
10. **System Performance**:
    - MACBiG-Net Performance: Accuracy = 85%, Precision = 81.3%, Recall = 81.2%, F1 Score = 81.2%
    - Sentiment Observations: Negative Sentiments (late February to mid-March, correlating with rising COVID-19 cases); Positive Sentiments (April, with positive messages, including those from Nelson Mandela, encouraging mask-wearing and resilience); Overall Sentiment Distribution: Negative = 39.6%, Positive = 32.6%, Neutral = 27.7%
11. **Domain Coverage**: General Domain
12. **Modality**: Text
13. **Primary EPHF**: EPHF 11
14. **Secondary EPHFs**: EPHF 1, EPHF 2
15. **Target Users**: Researchers and Data Scientists, Public Health Officials and Policymakers
16. **Public Health Challenge**: Understanding the global impact of COVID-19 through a language-independent sentiment analysis network, offering insights into public emotions and responses across diverse languages and regions.
17. **Public Health Outcomes**: Not Mentioned
18. **Levels of Impact and Engagement**: Global Public Health Promotion with Indirect African Impact
19. **Public Health Infrastructure Integration**: False
20. **Interoperability**: True
21. **SDGs**: SDG 3, SDG 10
22. **SDG 3 Targets**: 3.d, 3.3
23. **Year of Publication**: 2021
24. **Year of Data Collection**: 2020
25. **Deployment Stage**: Design and Prototyping
26. **Level of Accessibility**: Publicly Available
27. **Available Platform**: NLP Tools and Libraries
28. **Ethical Approval**: False
29. **Data Privacy Compliance**: Not Mentioned
30. **Informed Consent**: False
31. **Community Involvement**: False
32. **Stakeholder Collaboration**: True
33. **Other Ethical Considerations**: Not Mentioned

Towards Automating Healthcare Question Answering in a Noisy Multilingual Low-Resource Setting [26]

1. **Origin of the Work**: South Africa
2. **Affiliations**: Stellenbosch University, Praekelt Foundation, South Africa's National Department of Health (NDOH), CSIR Centre for Artificial Intelligence Research
3. **Types of Funding**: Research Grant
4. **Continents of Funders**: Africa
5. **Supported Languages**: Afrikaans, English, North Ndebele, Northern Sotho, Sotho, Swati, Tsonga, Tswana, Venda, Xhosa, Zulu
6. **Target African Countries or Regions**: South Africa
7. **NLP Application**: Question Answering
8. **Evaluation Method**: Technical Performance
9. **Technical Evaluation Metrics**: Accuracy, Recall@5
10. **System Performance**:
    - LSTM-512 Model: 62.13% accuracy on the full test set, 54.95% accuracy on low-resource test set
    - Recall@5: 89.56% on the full test set, 81.23% on the low-resource test set
11. **Domain Coverage**: General Domain
12. **Modality**: Text
13. **Primary EPHF**: EPHF 10
14. **Secondary EPHF**: EPHF 11
15. **Target Users**: Healthcare Providers, Researchers and Data Scientists, General Public
16. **Public Health Challenge**: Improving access to reliable healthcare information
17. **Public Health Outcomes**: Demonstrated potential to enhance response times on the MomConnect platform by automating question-answering, with a system accuracy of 62.13% and Recall@5 of 89.56%, suitable for a semi-automated environment
18. **Levels of Impact and Engagement**: Direct Impact on Public Health in Africa
19. **Public Health Infrastructure Integration**: True
20. **Interoperability**: True
21. **SDGs**: SDG 3, SDG 9, SDG 10
22. **SDG 3 Target**: 3.8
23. **Year of Publication**: 2019
24. **Year of Data Collection**: 2018
25. **Deployment Stage**: Design and Prototyping
26. **Level of Accessibility**: Closed-Access
27. **Available Platform**: NLP Tools and Libraries, Datasets
28. **Ethical Approval**: True
29. **Data Privacy Compliance**: True
30. **Informed Consent**: False
31. **Community Involvement**: True
32. **Stakeholder Collaboration**: True
33. **Other Ethical Considerations**: True

Evaluation of a Runyankore Grammar Engine for Healthcare Messages [27]

1. **Origin of the Work**: South Africa
2. **Affiliations**: University of Cape Town, Hasso Plattner Institute (HPI), National Research Foundation of South Africa
3. **Types of Funding**: Government Funding
4. **Continents of Funders**: Africa
5. **Supported Languages**: Nyankole
6. **Target African Countries or Regions**: Uganda
7. **NLP Application**: Syntax Parsing
8. **Evaluation Method**: Technical Performance, User Experience
9. **Technical Evaluation Metrics**: Accuracy
10. **System Performance**:
    - Over 50% of study participants evaluated most sentences as grammatically correct and understandable.
11. **Domain Coverage**: General Domain
12. **Modality**: Text
13. **Primary EPHF**: EPHF 10
14. **Secondary EPHF**: EPHF 11
15. **Target Users**: General Public, Healthcare Providers, Researchers and Data Scientists
16. **Public Health Challenge**: Improving communication and comprehension of healthcare messages
17. **Public Health Outcomes**: Most healthcare messages generated by the grammar engine were regarded as grammatically correct and understandable by native Runyankore speakers, indicating the tool's potential to enhance healthcare communication.
18. **Levels of Impact and Engagement**: Direct Impact on Public Health in Africa
19. **Public Health Infrastructure Integration**: True
20. **Interoperability**: True
21. **SDGs**: SDG 3, SDG 9
22. **SDG 3 Target**: 3.8
23. **Year of Publication**: 2017
24. **Year of Data Collection**: Not Mentioned
25. **Deployment Stage**: Design and Prototyping
26. **Level of Accessibility**: Publicly Available
27. **Available Platform**: NLP Tools and Libraries
28. **Ethical Approval**: False
29. **Data Privacy Compliance**: False
30. **Informed Consent**: False
31. **Community Involvement**: True
32. **Stakeholder Collaboration**: True
33. **Other Ethical Considerations**: True

Semantics of Body Parts in African WordNet: A Case of Northern Sotho [28]

1. **Origin of the Work**: South Africa
2. **Affiliations**: University of South Africa (UNISA), Internal University Research Funding
3. **Types of Funding**: Not Mentioned
4. **Continents of Funders**: Not Mentioned
5. **Supported Languages**: Northern Sotho
6. **Target African Countries or Regions**: South Africa
7. **NLP Application**: Lexical Processing
8. **Evaluation Method**: Technical Performance
9. **Technical Evaluation Metrics**: Accuracy, Linguistic Validity
10. **System Performance**:
    - Lexical Alignment: Identification of one-to-one, one-to-many, and many-to-one mappings
    - Lexicalisation Gaps: Identification of non-lexicalised concepts in Northern Sotho and English
11. **Domain Coverage**: General Domain
12. **Modality**: Text
13. **Primary EPHF**: EPHF 11
14. **Secondary EPHF**: EPHF 10
15. **Target Users**: Researchers and Data Scientists, General Public, Public Health Officials and Policymakers
16. **Public Health Challenge**: Enhancing the understanding and accurate communication of healthcare information
17. **Public Health Outcomes**: Not Mentioned
18. **Levels of Impact and Engagement**: Direct Impact on Public Health in Africa
19. **Public Health Infrastructure Integration**: False
20. **Interoperability**: False
21. **SDGs**: SDG 3, SDG 4
22. **SDG 3 Target**: 3.8
23. **Year of Publication**: 2016
24. **Year of Data Collection**: Not Mentioned
25. **Deployment Stage**: Design and Prototyping
26. **Level of Accessibility**: Publicly Available
27. **Available Platform**: NLP Tools and Libraries
28. **Ethical Approval**: False
29. **Data Privacy Compliance**: False
30. **Informed Consent**: False
31. **Community Involvement**: True
32. **Stakeholder Collaboration**: True
33. **Other Ethical Considerations**: True

People's Perceptions on COVID-19 Vaccination: An Analysis of Twitter Discourse from Four Countries [29]

1. **Origin of the Work**: India, United States of America, Saudi Arabia, Lebanon
2. **Affiliations**: Thapar Institute of Engineering and Technology, Tata Institute of Social Sciences, University of Petroleum and Energy Studies, King Abdulaziz University, Lebanese American University, RIMT University, Central University of Kerala
3. **Types of Funding**: Not Mentioned
4. **Continents of Funders**: Not Mentioned
5. **Supported Languages**: English
6. **Target African Countries or Regions**: South Africa
7. **NLP Application**: Sentiment Analysis
8. **Evaluation Method**: Technical Performance
9. **Technical Evaluation Metrics**: Accuracy, Precision, Krippendorff's Alpha
10. **System Performance**:
    - Accuracy: 89% (RoBERTa model)
    - Precision: 87% (RoBERTa model)
    - Krippendorff's Alpha: Positive tweets (0.73), Negative tweets (0.74), Neutral tweets (0.67)
    - Analysis: Identified themes and attitudes toward COVID-19 vaccination.
11. **Domain Coverage**: General Domain
12. **Modality**: Text
13. **Primary EPHF**: EPHF 1
14. **Secondary EPHF**: EPHF 7, EPHF 2
15. **Target Users**: Public Health Officials and Policymakers, Researchers and Data Scientists
16. **Public Health Challenge**: Understanding people's perceptions and attitudes toward COVID-19 vaccination
17. **Public Health Outcomes**: The study highlights differences in vaccine perceptions across countries, identifying themes such as vaccine safety, efficacy, and equity, which can inform public health interventions.
18. **Levels of Impact and Engagement**: Global health discussions with African examples
19. **Public Health Infrastructure Integration**: False
20. **Interoperability**: False
21. **SDGs**: SDG 3
22. **SDG 3 Target**: 3.b, 3.3
23. **Year of Publication**: 2023
24. **Year of Data Collection**: 2020-2022
25. **Deployment Stage**: Design and Prototyping
26. **Level of Accessibility**: Publicly Available
27. **Available Platform**: NLP Tools and Libraries
28. **Ethical Approval**: False
29. **Data Privacy Compliance**: True
30. **Informed Consent**: False
31. **Community Involvement**: True
32. **Stakeholder Collaboration**: True
33. **Other Ethical Considerations**: True

Health Chatbots in Africa: Scoping Review [30]

1. **Origin of the Work**: South Africa
2. **Affiliations**: University of Johannesburg
3. **Types of Funding**: Government Funding
4. **Continents of Funders**: Europe
5. **Supported Languages**: English, French, Arabic, Central Atlas Tamazight, Igbo, Yoruba, Hausa
6. **Target African Countries or Regions**: Nigeria, South Africa, Tunisia, Kenya
7. **NLP Application**: Conversational Assistant
8. **Evaluation Method**: Technical Performance, Health Research Measure
9. **Technical Evaluation Metrics**: User Satisfaction, User Engagement
10. **System Performance**:
    - Improved access to health information and services
    - Application across various health domains, including HIV counseling, COVID-19 information dissemination, maternal health
11. **Domain Coverage**: General Domain
12. **Modality**: Text
13. **Primary EPHF**: EPHF 7
14. **Secondary EPHF**: EPHF 10
15. **Target Users**: General Public, Healthcare Providers, Public Health Officials and Policymakers
16. **Public Health Challenge**: Assessing the effectiveness and potential of health chatbots in improving healthcare delivery in Africa
17. **Public Health Outcomes**: The review finds potential benefits of chatbots in specific areas, such as HIV prevention and maternal health, though high-quality data on overall effectiveness remains limited.
18. **Levels of Impact and Engagement**: Direct impact on public health in Africa
19. **Public Health Infrastructure Integration**: True
20. **Interoperability**: True
21. **SDGs**: SDG 3
22. **SDG 3 Target**: 3.8
23. **Year of Publication**: 2023
24. **Year of Data Collection**: 2017-2022
25. **Deployment Stage**: Not Applicable
26. **Level of Accessibility**: Accessibility Irrelevant
27. **Available Platform**: Not Applicable
28. **Ethical Approval**: False
29. **Data Privacy Compliance**: True
30. **Informed Consent**: True
31. **Community Involvement**: True
32. **Stakeholder Collaboration**: True
33. **Other Ethical Considerations**: True

Artificial Intelligence and Machine Learning for HIV Prevention: Emerging Approaches to Ending the Epidemic [31]

1. **Origin of the Work**: United States of America
2. **Affiliations**: National Institute of Allergy and Infectious Diseases, President's Emergency Plan for AIDS Relief (PEPFAR)
3. **Types of Funding**: Government Funding
4. **Continents of Funders**: North America
5. **Supported Languages**: English
6. **Target African Countries or Regions**: Kenya, Uganda
7. **NLP Application**: Conversational Assistant
8. **Evaluation Method**: Technical Performance
9. **Technical Evaluation Metrics**: AUC, Predictive Performance
10. **System Performance**:
    - AUC: 0.86 for LASSO, 0.84 for EHR-based prediction tool, 0.88 for Ridge Regression in Denmark
    - Sensitivity: Improved identification of PrEP candidates using machine learning models
    - Higher AUC for ML algorithms compared to traditional models
    - Positive feedback from qualitative assessments with end users
11. **Domain Coverage**: Clinical Domain
12. **Modality**: Text
13. **Primary EPHF**: EPHF 6
14. **Secondary EPHF**: EPHF 9, EPHF 11
15. **Target Users**: Healthcare Providers, Public Health Officials and Policymakers, Researchers and Data Scientists
16. **Public Health Challenge**: Developing innovative approaches for HIV prevention to end the epidemic through enhanced detection, intervention, and treatment strategies
17. **Public Health Outcomes**: Not Mentioned
18. **Levels of Impact and Engagement**: Global Public Health Promotion with Indirect African Impact
19. **Public Health Infrastructure Integration**: True
20. **Interoperability**: True
21. **SDGs**: SDG 3, SDG 10
22. **SDG 3 Target**: 3.3
23. **Year of Publication**: 2020
24. **Year of Data Collection**: Not Mentioned
25. **Deployment Stage**: Not Applicable
26. **Level of Accessibility**: Accessibility Irrelevant
27. **Available Platform**: Not Applicable
28. **Ethical Approval**: True
29. **Data Privacy Compliance**: True
30. **Informed Consent**: False
31. **Community Involvement**: True
32. **Stakeholder Collaboration**: True
33. **Other Ethical Considerations**: True

Preliminary Reliability of South African Adaptation and Northern Sotho Translation of the Modified Checklist for Autism in Toddlers, Revised with Follow-Up [32]

1. **Origin of the Work**: South Africa
2. **Affiliations**: University of Pretoria, Organization for Autism Research (2020G11)
3. **Types of Funding**: NGO Funding
4. **Continents of Funders**: North America
5. **Supported Languages**: English, Northern Sotho
6. **Target African Countries or Regions**: South Africa
7. **NLP Application**: Machine Translation
8. **Evaluation Method**: Technical Performance
9. **Technical Evaluation Metrics**: Annotation Agreement
10. **System Performance**:
    - Screening for autism risk in toddlers
    - High agreement between English and Northern Sotho versions
11. **Domain Coverage**: General Domain
12. **Modality**: Text
13. **Primary EPHF**: EPHF 10
14. **Secondary EPHF**: EPHF 11
15. **Target Users**: Healthcare Providers, Public Health Officials and Policymakers, Researchers and Data Scientists, General Public
16. **Public Health Challenge**: Improving early detection and diagnosis of autism in South Africa
17. **Public Health Outcomes**: Preliminary findings indicate that the adapted tools are reliable and feasible for South African use, potentially improving early detection and intervention for autism in Northern Sotho-speaking children.
18. **Levels of Impact and Engagement**: Systems or analyses developed with African data
19. **Public Health Infrastructure Integration**: True
20. **Interoperability**: True
21. **SDGs**: SDG 3, SDG 4
22. **SDG 3 Target**: 3.2
23. **Year of Publication**: 2021
24. **Year of Data Collection**: 2019-2020
25. **Deployment Stage**: Design and Prototyping
26. **Level of Accessibility**: Limited Access
27. **Available Platform**: NLP Tools and Libraries
28. **Ethical Approval**: True
29. **Data Privacy Compliance**: True
30. **Informed Consent**: True
31. **Community Involvement**: True
32. **Stakeholder Collaboration**: True
33. **Other Ethical Considerations**: True

Social, Behavioral, and Cultural Factors of HIV in Malawi: Semi-Automated Systematic Review [33]

1. **Origin of the Work**: Switzerland, Italy
2. **Affiliations**: University of Geneva, Scuola Superiore Sant'Anna, University of Bern, Swiss National Foundation (SNF)
3. **Types of Funding**: Research Grant, NGO Funding
4. **Continents of Funders**: Europe
5. **Supported Languages**: English
6. **Target African Countries or Regions**: Malawi
7. **NLP Application**: Thematic Analysis
8. **Evaluation Method**: Technical Performance
9. **Technical Evaluation Metrics**: Frequency
10. **System Performance**:
    - Reduced 16,942 articles to 519 potentially relevant articles, further screened to 27 final articles
    - Processing Speed: Software completed initial dataset reduction in 5 days
    - Identification of factors influencing HIV prevalence and incidence in Malawi
11. **Domain Coverage**: General Domain
12. **Modality**: Text
13. **Primary EPHF**: EPHF 11
14. **Secondary EPHF**: EPHF 7
15. **Target Users**: Public Health Officials and Policymakers, Researchers and Data Scientists, General Public
16. **Public Health Challenge**: Understanding social, behavioral, and cultural factors affecting HIV prevalence and transmission in Malawi
17. **Public Health Outcomes**: Not Mentioned
18. **Levels of Impact and Engagement**: Systems or analyses developed with African data
19. **Public Health Infrastructure Integration**: True
20. **Interoperability**: True
21. **SDGs**: SDG 3, SDG 5
22. **SDG 3 Target**: 3.3
23. **Year of Publication**: 2020
24. **Year of Data Collection**: 1987-2019
25. **Deployment Stage**: Not Applicable
26. **Level of Accessibility**: Accessibility Irrelevant
27. **Available Platform**: Not Applicable
28. **Ethical Approval**: False
29. **Data Privacy Compliance**: False
30. **Informed Consent**: False
31. **Community Involvement**: False
32. **Stakeholder Collaboration**: True
33. **Other Ethical Considerations**: True

Real-Time Infoveillance of Moroccan Social Media Users' Sentiments towards the COVID-19 Pandemic and Its Management [34]

1. **Origin of the Work**: Morocco
2. **Affiliations**: International University of Rabat, Mohammed V University in Rabat, University Ibn Tofail, Centre National de la Recherche Scientifique et Technique (CNRST)
3. **Types of Funding**: Government Funding
4. **Continents of Funders**: Africa
5. **Supported Languages**: Arabic
6. **Target African Countries or Regions**: Morocco
7. **NLP Application**: Sentiment Analysis
8. **Evaluation Method**: Technical Performance
9. **Technical Evaluation Metrics**: Accuracy (MultinomialNB, Logistic Regression, SVM, MD-ULM)
10. **System Performance**:
    - **Model Accuracy**:
      - **MultinomialNB**: 0.31 (Emotion), 0.51 (Topic), 0.64 (Polarity)
      - **Logistic Regression**: 0.33 (Emotion), 0.53 (Topic), 0.61 (Polarity)
      - **SVM**: 0.33 (Emotion), 0.59 (Topic), 0.65 (Polarity)
      - **MD-ULM**: 0.43 (Emotion), 0.70 (Topic), 0.70 (Polarity)
    - Analyzed 13,651 comments, with 6.73% positive comments, and anger as the dominant emotion (50.52%).
    - Dominant Topic: Statistics (39.43%)
11. **Domain Coverage**: General Domain
12. **Modality**: Text
13. **Primary EPHF**: EPHF 1
14. **Secondary EPHF**: EPHF 7, EPHF 5
15. **Target Users**: Public Health Officials and Policymakers, Researchers and Data Scientists
16. **Public Health Challenge**: Monitoring and understanding real-time sentiments of Moroccan social media users towards COVID-19 and its management to guide public health response and crisis communication.
17. **Public Health Outcomes**: Not Mentioned
18. **Levels of Impact and Engagement**: Systems or analyses developed with African data
19. **Public Health Infrastructure Integration**: False
20. **Interoperability**: True
21. **SDGs**: SDG 3
22. **SDG 3 Target**: 3.d, 3.3
23. **Year of Publication**: 2021
24. **Year of Data Collection**: 2020
25. **Deployment Stage**: Design and Prototyping
26. **Level of Accessibility**: Publicly Available
27. **Available Platform**: Web-based Applications
28. **Ethical Approval**: False
29. **Data Privacy Compliance**: True
30. **Informed Consent**: False
31. **Community Involvement**: True
32. **Stakeholder Collaboration**: True
33. **Other Ethical Considerations**: Others

The Promise of Clinical Decision Support Systems Targetting Low-Resource Settings [35]

1. **Origin of the Work**: United Kingdom
2. **Affiliations**: University of Oxford
3. **Types of Funding**: Not Mentioned
4. **Continents of Funders**: Not Mentioned
5. **Supported Languages**: English
6. **Target African Countries or Regions**: South Africa, Rwanda, Zimbabwe
7. **NLP Applications**: Conversational Assistant
8. **Evaluation Method**: Technical Performance, Health Research Measure
9. **Technical Evaluation Metrics**: AUC, User Satisfaction
10. **System Performance**: Not Applicable
11. **Domain Coverage**: General Domain
12. **Modality**: Text
13. **Primary EPHF**: EPHF 10
14. **Secondary EPHF**: EPHF 11, EPHF 9
15. **Target Users**: Healthcare Providers, Public Health Officials and Policymakers, Researchers and Data Scientists
16. **Public Health Challenge**: Enhancing healthcare delivery in low-resource settings by exploring clinical decision support systems (CDSSs) to improve diagnostic accuracy, treatment outcomes, and overall patient care.
17. **Public Health Outcomes**: The review highlights studies suggesting CDSSs can improve healthcare outcomes in low-resource settings, such as better diagnosis, treatment planning, and disease management for tuberculosis, HIV, and maternal health issues. The need for more research to fully assess these impacts is noted.
18. **Levels of Impact and Engagement**: Global Public Health Promotion with Indirect African Impact
19. **Public Health Infrastructure Integration**: True
20. **Interoperability**: True
21. **SDGs**: SDG 3, SDG 9, SDG 10, SDG 8
22. **SDG 3 Target**: 3.d, 3.c
23. **Year of Publication**: 2020
24. **Year of Data Collection**: Not Mentioned
25. **Deployment Stage**: Design and Prototyping
26. **Level of Accessibility**: Limited Access
27. **Available Platform**: Web-based Applications
28. **Ethical Approval**: False
29. **Data Privacy Compliance**: False
30. **Informed Consent**: False
31. **Community Involvement**: False
32. **Stakeholder Collaboration**: True
33. **Other Ethical Considerations**: True

Forecasting influenza-like illness trends in Cameroon using Google Search Data [36]

1. **Origin of the Work**: Cameroon
2. **Affiliations**: Boston University, Ministry of Health (Cameroon), Texas A&M University, National Institutes of Health (NIH), Texas A&M University faculty startup funding
3. **Types of Funding**: Government Funding
4. **Continents of Funders**: North America
5. **Supported Languages**: English, French
6. **Target African Countries or Regions**: Cameroon
7. **NLP Applications**: Outbreak Detection
8. **Evaluation Method**: Technical Performance
9. **Technical Evaluation Metrics**: R-squared (R^2), SVM, RF, ARIMA, RMSE
10. **System Performance**: R-squared (R^2): SVM: 0.877, RF: 0.781; ARIMA with covariates: Comparable to RF; ARIMA without covariates: Comparable to RF RMSE: SVM: 1.078, RF: 1.41; ARIMA with covariates: Comparable to RF; ARIMA without covariates: Comparable to RF. The study achieved improved prediction accuracy for ILI trends in Cameroon using Google search data as explanatory variables.
11. **Domain Coverage**: General Domain
12. **Modality**: Text
13. **Primary EPHF**: EPHF 1
14. **Secondary EPHF**: EPHF 6
15. **Target Users**: Public Health Officials and Policymakers, Researchers and Data Scientists
16. **Public Health Challenge**: Predicting and monitoring influenza-like illness trends in Cameroon
17. **Public Health Outcomes**: The study demonstrates the potential of digital data to improve the timeliness and accuracy of ILI surveillance, which can lead to better public health outcomes.
18. **Levels of Impact and Engagement**: Systems or analyses developed with African data
19. **Public Health Infrastructure Integration**: False
20. **Interoperability**: False
21. **SDGs**: SDG 3
22. **SDG 3 Target**: 3.d
23. **Year of Publication**: 2021
24. **Year of Data Collection**: 2012-2018
25. **Deployment Stage**: Design and Prototyping
26. **Level of Accessibility**: Publicly Available
27. **Available Platform**: NLP Tools and Libraries
28. **Ethical Approval**: False
29. **Data Privacy Compliance**: True
30. **Informed Consent**: True
31. **Community Involvement**: False
32. **Stakeholder Collaboration**: True
33. **Other Ethical Considerations**: True

Detection of Hate Speech in COVID-19-Related Tweets in the Arab Region: Deep Learning and Topic Modeling Approach [37]

1. **Origin of the Work**: Saudi Arabia
2. **Affiliations**: King Saud University
3. **Types of Funding**: Government Funding
4. **Continents of Funders**: Asia
5. **Supported Languages**: Arabic
6. **Target African Countries or Regions**: Egypt
7. **NLP Applications**: Hate Speech Detection, Topic Modeling
8. **Evaluation Method**: Technical Performance
9. **Technical Evaluation Metrics**: Accuracy, F1 Score
10. **System Performance**: Accuracy: 83%, F1 Score: 79%; Identification of hate speech, with 3.2% of tweets classified as hate speech in the dataset
11. **Domain Coverage**: General Domain
12. **Modality**: Text
13. **Primary EPHF**: EPHF 1
14. **Secondary EPHF**: EPHF 7, EPHF 2
15. **Target Users**: Researchers and Data Scientists, Public Health Officials and Policymakers
16. **Public Health Challenge**: Identifying and mitigating hate speech in COVID-19-related tweets in the Arab region
17. **Public Health Outcomes**: Not Mentioned
18. **Levels of Impact and Engagement**: Global health discussions with African examples
19. **Public Health Infrastructure Integration**: True
20. **Interoperability**: True
21. **SDGs**: SDG 3, SDG 16
22. **SDG 3 Target**: 3.d, 3.3
23. **Year of Publication**: 2020
24. **Year of Data Collection**: 2020
25. **Deployment Stage**: 2020
26. **Level of Accessibility**: Limited Access
27. **Available Platform**: NLP Tools and Libraries
28. **Ethical Approval**: False
29. **Data Privacy Compliance**: True
30. **Informed Consent**: False
31. **Community Involvement**: False
32. **Stakeholder Collaboration**: True
33. **Other Ethical Considerations**: True

Chatbots for HIV Prevention and Care: a Narrative Review [38]

1. **Origin of the Work**: South Africa, United States of America
2. **Affiliations**: ZAF, USA
3. **Types of Funding**: Not Mentioned
4. **Continents of Funders**: Not Mentioned
5. **Supported Languages**: English
6. **Target African Countries or Regions**: South Africa
7. **NLP Applications**: Conversational Assistant
8. **Evaluation Method**: Technical Performance, User Experience
9. **Technical Evaluation Metrics**: Accuracy, User Engagement
10. **System Performance**: High user engagement with chatbots; users preferred chatbots for their perceived non-judgmental nature and increased anonymity
11. **Domain Coverage**: General Domain, Biomedical Domain
12. **Modality**: Text
13. **Primary EPHF**: EPHF 7
14. **Secondary EPHF**: EPHF 10, EPHF 2
15. **Target Users**: Specific equity-seeking groups, General Public, Researchers and Data Scientists
16. **Public Health Challenge**: Evaluating the effectiveness of chatbots in enhancing HIV prevention and care
17. **Public Health Outcomes**: Not Mentioned
18. **Levels of Impact and Engagement**: Global public health promotion with indirect African impact
19. **Public Health Infrastructure Integration**: True
20. **Interoperability**: True
21. **SDGs**: SDG 3
22. **SDG 3 Target**: 3.3
23. **Year of Publication**: 2023
24. **Year of Data Collection**: 2023
25. **Deployment Stage**: Not Applicable
26. **Level of Accessibility**: Accessibility Irrelevant
27. **Available Platform**: Not Applicable
28. **Ethical Approval**: False
29. **Data Privacy Compliance**: True
30. **Informed Consent**: False
31. **Community Involvement**: True
32. **Stakeholder Collaboration**: True
33. **Other Ethical Considerations**: True

Using text mining techniques to extract prostate cancer predictive information (Gleason score) from semi-structured narrative laboratory reports in the Gauteng province, South Africa [39]

1. **Origin of the Work**: South Africa
2. **Affiliations**: University of Witwatersrand and National Health Laboratory Service (NHLS)
3. **Types of Funding**: Not Mentioned
4. **Continents of Funders**: Not Mentioned
5. **Supported Languages**: English
6. **Target African Countries or Regions**: South Africa
7. **NLP Applications**: Information Extraction
8. **Evaluation Method**: Technical Performance
9. **Technical Evaluation Metrics**: Precision, Recall, F1-Score
10. **System Performance**: Precision: 1.00, Recall: 0.98 (initial), improved to 1.00, F1 Score: 0.99 (initial), improved to 1.00
11. **Domain Coverage**: Biomedical Domain, Clinical Domain
12. **Modality**: Text
13. **Primary EPHF**: EPHF 11
14. **Secondary EPHF**: EPHF 10, EPHF 5
15. **Target Users**: Healthcare Providers, Researchers and Data Scientists, Public Health Officials and Policymakers
16. **Public Health Challenge**: Improving the accuracy and efficiency of extracting predictive information for prostate cancer, specifically the Gleason score, from semi-structured narrative laboratory reports
17. **Public Health Outcomes**: Not Mentioned
18. **Levels of Impact and Engagement**: Systems or analyses developed with African data
19. **Public Health Infrastructure Integration**: True
20. **Interoperability**: True
21. **SDGs**: SDG 3
22. **SDG 3 Target**: 3.4
23. **Year of Publication**: 2021
24. **Year of Data Collection**: 2006-2016
25. **Deployment Stage**: Design and Prototyping
26. **Level of Accessibility**: Open-Sourced
27. **Available Platform**: NLP Tools and Libraries
28. **Ethical Approval**: True
29. **Data Privacy Compliance**: True
30. **Informed Consent**: False
31. **Community Involvement**: False
32. **Stakeholder Collaboration**: True
33. **Other Ethical Considerations**: True

The Feasibility and Acceptability of an mHealth Conversational Agent Designed to Support HIV Self-testing in South Africa: Cross-sectional Study [40]

1. **Origin of the Work**: South Africa, United States of America, Belgium
2. **Affiliations**: Centre for Community Based Research, Human Sciences Research Council, Institute of Tropical Medicine Antwerp, Belgium; Ghent University, Harvard Medical School, Massachusetts General Hospital, University of the Witwatersrand
3. **Types of Funding**: Not Mentioned
4. **Continents of Funders**: Not Mentioned
5. **Supported Languages**: Zulu
6. **Target African Countries or Regions**: South Africa
7. **NLP Applications**: Conversational Assistant
8. **Evaluation Method**: User Experience, Health Research Measure
9. **Technical Evaluation Metrics**: User Engagement, User Satisfaction
10. **System Performance**: High user engagement: 79.2% found the experience better than human counseling. Realness: 77.5% felt the chatbot interaction was similar to talking to a real person
11. **Domain Coverage**: General Domain
12. **Modality**: Text
13. **Primary EPHF**: EPHF 7
14. **Secondary EPHF**: EPHF 10
15. **Target Users**: General public, Healthcare providers, Researchers and data scientists, Public health officials and policymakers
16. **Public Health Challenge**: Improving access to and support for HIV self-testing in South Africa by evaluating the feasibility and acceptability of an mHealth conversational agent designed to assist users through the self-testing process
17. **Public Health Outcomes**: Not Mentioned
18. **Levels of Impact and Engagement**: Direct impact on public health in Africa
19. **Public Health Infrastructure Integration**: True
20. **Interoperability**: True
21. **SDGs**: SDG 3
22. **SDG 3 Target**: 3.3
23. **Year of Publication**: 2022
24. **Year of Data Collection**: 2020-2021
25. **Deployment Stage**: Design and Prototyping
26. **Level of Accessibility**: Limited Access
27. **Available Platform**: Mobile Applications
28. **Ethical Approval**: True
29. **Data Privacy Compliance**: True
30. **Informed Consent**: True
31. **Community Involvement**: True
32. **Stakeholder Collaboration**: True
33. **Other Ethical Considerations**: True

Vaccines for pregnant women…?! Absurd"–Mapping maternal vaccination discourse and stance on social media over six months [41]

1. **Origin of the Work**: United Kingdom, Netherlands, Spain, United States of America
2. **Affiliations**: London School of Hygiene & Tropical Medicine
3. **Types of Funding**: Industry Funding
4. **Continents of Funders**: Europe
5. **Supported Languages**: English
6. **Target African Countries or Regions**: South Africa
7. **NLP Applications**: Semantics Parsing
8. **Evaluation Method**: Technical Performance
9. **Technical Evaluation Metrics**: Stance Analysis
10. **System Performance**: For South Africa, there are 3 forums and 108 Twitter posts, making up 0.7% of the total tweets analyzed. Posts illustrating relationships between emotion and suggested fetal loss or disability in association with maternal vaccination were included. A higher proportion of discouraging tweets were observed in South Africa (ranging from 7% in Nov-Dec to 28% in Jan-Feb). Stance Analysis involved categorizing posts as promotional, neutral, ambiguous, or discouraging.
11. **Domain Coverage**: General Domain
12. **Modality**: Text
13. **Primary EPHF**: EPHF 7
14. **Secondary EPHF**: EPHF 1
15. **Target Users**: Public Health Officials and Policymakers, Researchers and Data Scientists
16. **Public Health Challenge**: Understanding public perceptions and stances on maternal vaccination by mapping and analyzing social media discourse over six months to inform better communication strategies and improve vaccination uptake among pregnant women.
17. **Public Health Outcomes**: Not Mentioned
18. **Levels of Impact and Engagement**: Global Health Discussions with African Examples
19. **Public Health Infrastructure Integration**: True
20. **Interoperability**: True
21. **SDGs**: SDG 3, SDG 5, SDG 10
22. **SDG 3 Target**: 3.1, 3.2, 3.b
23. **Year of Publication**: 2020
24. **Year of Data Collection**: Not Mentioned
25. **Deployment Stage**: Design and Prototyping
26. **Level of Accessibility**: Limited Access
27. **Available Platform**: NLP Tools and Libraries
28. **Ethical Approval**: False
29. **Data Privacy Compliance**: True
30. **Informed Consent**: False
31. **Community Involvement**: False
32. **Stakeholder Collaboration**: True
33. **Other Ethical Considerations**: True

Positive attitudes towards COVID-19 vaccines: A cross-country analysis [42]

1. **Origin of the Work**: South Africa, New Zealand
2. **Affiliations**: University of Johannesburg, Auckland University of Technology
3. **Types of Funding**: Not Mentioned
4. **Continents of Funders**: Not Mentioned
5. **Supported Languages**: English
6. **Target African Countries or Regions**: South Africa
7. **NLP Applications**: Sentiment Analysis
8. **Evaluation Method**: Technical Performance
9. **Technical Evaluation Metrics**: IV Regression, FE Estimation, Pooled Ordinary Least Squares
10. **System Performance**: Significant factors influencing vaccine attitudes include trust in vaccines, which positively affects attitudes, while compliance with regulations and anger towards the government inversely impact positive vaccine attitudes.
11. **Domain Coverage**: General Domain
12. **Modality**: Text
13. **Primary EPHF**: EPHF 7
14. **Secondary EPHF**: EPHF 1, EPHF 2
15. **Target Users**: General Public, Researchers and Data Scientists, Public Health Officials and Policymakers
16. **Public Health Challenge**: Understanding and Promoting Positive Attitudes Towards COVID-19 Vaccines
17. **Public Health Outcomes**: Not Mentioned
18. **Levels of Impact and Engagement**: Global Public Health Promotion with Indirect African Impact
19. **Public Health Infrastructure Integration**: True
20. **Interoperability**: True
21. **SDGs**: SDG 3
22. **SDG 3 Target**: 3.b, 3.3
23. **Year of Publication**: 2022
24. **Year of Data Collection**: 2021
25. **Deployment Stage**: Design and Prototyping
26. **Level of Accessibility**: Publicly Available
27. **Available Platform**: NLP Tools and Libraries
28. **Ethical Approval**: False
29. **Data Privacy Compliance**: True
30. **Informed Consent**: False
31. **Community Involvement**: False
32. **Stakeholder Collaboration**: True
33. **Other Ethical Considerations**: True

The accuracy of radiology speech recognition reports in a multilingual South African teaching hospital [43]

1. **Origin of the Work**: South Africa
2. **Affiliations**: Tygerberg Academic Hospital, Stellenbosch University
3. **Types of Funding**: Not Mentioned
4. **Continents of Funders**: Not Mentioned
5. **Supported Languages**: English
6. **Target African Countries or Regions**: South Africa
7. **NLP Applications**: Speech Recognition
8. **Evaluation Method**: Technical Performance
9. **Technical Evaluation Metrics**: Error Rates
10. **System Performance**: SR reports: 25.6% error rate, 9.6% clinically significant errors; DT reports: 9.3% error rate, 2.3% clinically significant errors; Follow-up SR reports: 24.3% error rate, 6% clinically significant errors.
11. **Domain Coverage**: Clinical Domain
12. **Modality**: Text, Audio
13. **Primary EPHF**: EPHF 9
14. **Secondary EPHF**: EPHF 10
15. **Target Users**: Healthcare Providers, Researchers and Data Scientists, Public Health Officials and Policymakers
16. **Public Health Challenge**: Evaluating the accuracy of radiology speech recognition reports in a multilingual South African teaching hospital to ensure reliable and effective communication of diagnostic information across different languages.
17. **Public Health Outcomes**: The study found that SR reports had a higher error rate compared to traditional dictation transcription (DT), with significant potential for clinical errors, potentially compromising patient care.
18. **Levels of Impact and Engagement**: Systems or Analyses Developed with African Data
19. **Public Health Infrastructure Integration**: True
20. **Interoperability**: True
21. **SDGs**: SDG 3, SDG 10, SDG 8
22. **SDG 3 Target**: 3.8, 3.c
23. **Year of Publication**: 2015
24. **Year of Data Collection**: 2010-2014
25. **Deployment Stage**: Design and Prototyping
26. **Level of Accessibility**: Limited Access
27. **Available Platform**: Mobile Applications, Web-Based Applications
28. **Ethical Approval**: True
29. **Data Privacy Compliance**: True
30. **Informed Consent**: False
31. **Community Involvement**: False
32. **Stakeholder Collaboration**: True
33. **Other Ethical Considerations**: True

Off-label drug use during the COVID-19 pandemic in Africa: topic modelling and sentiment analysis of ivermectin in South Africa and Nigeria as a case study [44]

1. **Origin of the Work**: Canada, South Africa, Iran
2. **Affiliations**: Africa-Canada Artificial Intelligence and Data Innovation Consortium, K.N. Toosi University, Tehran, Iran, University of the Witwatersrand, South Africa, York University, Canada
3. **Types of Funding**: Not mentioned
4. **Continents of Funders**: Not mentioned
5. **Supported Languages**: English
6. **Target African Countries or Regions**: South Africa, Nigeria
7. **NLP Applications**: Sentiment Analysis, Topic Modeling, Stance Detection
8. **Evaluation Method**: Technical Performance
9. **Technical Evaluation Metrics**: Precision, Recall, F1 Score, Accuracy, Correlation
10. **System Performance**: Stance Detection Model Accuracy: 73%, Sentiment Analysis Model Accuracy: 72%, Gender Recognition Model Accuracy: 86%
11. **Domain Coverage**: General Domain
12. **Modality**: Text
13. **Primary EPHF**: EPHF 1 (Public Health Surveillance and Monitoring)
14. **Secondary EPHF**: EPHF 7, EPHF 5
15. **Target Users**: Public Health Officials and Policymakers, Researchers and Data Scientists, General Public
16. **Public Health Challenge**: Understanding the prevalence and perceptions of off-label drug use during the COVID-19 pandemic in Africa
17. **Public Health Outcomes**: Not mentioned
18. **Levels of Impact and Engagement**: Systems or Analyses Developed with African Data
19. **Public Health Infrastructure Integration**: True
20. **Interoperability**: False
21. **SDGs**: SDG 3
22. **SDG 3 Target**: 3.b, 3.3
23. **Year of Publication**: 2023
24. **Year of Data Collection**: 2020-2022
25. **Deployment Stage**: Design and Prototyping
26. **Level of Accessibility**: Publicly Available
27. **Available Platform**: NLP Tools and Libraries
28. **Ethical Approval**: Not mentioned (False)
29. **Data Privacy Compliance**: True
30. **Informed Consent**: False
31. **Community Involvement**: False
32. **Stakeholder Collaboration**: False
33. **Other Ethical Considerations**: None Mentioned

Rule-Based Information Extraction from Free-Text Pathology Reports Reveals Trends in South African Female Breast Cancer Molecular Subtypes and Ki67 Expression [45]

1. **Origin of the Work**: South African
2. **Affiliations**: University of the Witwatersrand, South Africa, National Cancer Registry, South Africa, African Union Development Agency, Netherlands (Utrecht University); Supported by the DELTAS Africa Initiative
3. **Types of Funding**: NGO Funding
4. **Continents of Funders**: Africa
5. **Supported Languages**: English
6. **Target African Countries or Regions**: South Africa
7. **NLP Applications**: Information Extraction Using Rule-Based Methods
8. **Evaluation Method**: Technical Performance
9. **Technical Evaluation Metrics**: Precision, Recall, F1 Score, Annotator Agreement
10. **System Performance**: Precision: 83-100%, Recall: 93-100%, F1 Score: 91-100%, Kappa: 92-100%; High precision, recall, and F1 scores for most extracted parameters with high agreement between machine-extracted and manually extracted data.
11. **Domain Coverage**: General Domain
12. **Modality**: Text
13. **Primary EPHF**: EPHF 11
14. **Secondary EPHF**: EPHF 10, EPHF 5
15. **Target Users**: Researchers and Data Scientists, Healthcare Providers (Pathologists and Oncologists), Public Health Officials and Policymakers
16. **Public Health Challenge**: Understanding trends in breast cancer molecular subtypes and Ki67 expression in South African women by extracting information from pathology reports, aiding in more tailored and effective treatment strategies.
17. **Public Health Outcomes**: Identified trends in breast cancer subtypes and Ki67 expression, potentially improving public health strategies and interventions for breast cancer treatment and diagnosis in South Africa.
18. **Levels of Impact and Engagement**: Systems or Analyses Developed with African Data
19. **Public Health Infrastructure Integration**: True
20. **Interoperability**: True
21. **SDGs**: SDG 3
22. **SDG 3 Target**: 3.4
23. **Year of Publication**: 2022
24. **Year of Data Collection**: Not Mentioned
25. **Deployment Stage**: Design and Prototyping
26. **Level of Accessibility**: Open-Sourced
27. **Available Platform**: NLP Tools and Libraries
28. **Ethical Approval**: Not Mentioned
29. **Data Privacy Compliance**: True
30. **Informed Consent**: False
31. **Community Involvement**: False
32. **Stakeholder Collaboration**: True
33. **Other Ethical Considerations**: True

Nowcasting Unemployment Rate During the COVID-19 Pandemic Using Twitter Data: The Case of South Africa [46]

1. **Origin of the Work**: South Africa and Canada
2. **Affiliations**: Canada's International Development Research Centre (IDRC), Swedish International Development Cooperation Agency (SIDA), York University, Toronto, ON, Canada, University of the Witwatersrand, South Africa
3. **Types of Funding**: NGO Funding
4. **Continents of Funders**: North America, Europe
5. **Supported Languages**: English
6. **Target African Countries or Regions**: South Africa
7. **NLP Applications**: Sentiment Analysis
8. **Evaluation Method**: Technical Performance
9. **Technical Evaluation Metrics**: RMSE: 0.921, MAPE: 0.018, SMAPE: 0.018, R2-score: 0.929
10. **System Performance**: The model demonstrated strong performance with low error metrics across RMSE, MAPE, SMAPE, and R2-score.
11. **Domain Coverage**: General Domain
12. **Modality**: Text
13. **Primary EPHF**: Public Health Surveillance and Monitoring
14. **Secondary EPHF**: Public Health Research, Evaluation, and Knowledge (EPHF 7)
15. **Target Users**: Public health officials and policymakers, Researchers and data scientists
16. **Public Health Challenge**: Providing real-time estimates of the unemployment rate during the COVID-19 pandemic in South Africa.
17. **Public Health Outcomes**: Not directly mentioned, but insights could inform public health and economic strategies during crises.
18. **Levels of Impact and Engagement**: Systems or Analyses Developed with African Data
19. **Public Health Infrastructure Integration**: True
20. **Interoperability**: True
21. **SDGs**: SDG 3, SDG 8
22. **SDG 3 Target**: 3.d, 3.3, 3.c
23. **Year of Publication**: 2022
24. **Year of Data Collection**: 2021
25. **Deployment Stage**: Design and Prototyping
26. **Level of Accessibility**: Open-Sourced
27. **Available Platform**: NLP Tools and Libraries
28. **Ethical Approval**: False
29. **Data Privacy Compliance**: True
30. **Informed Consent**: False
31. **Community Involvement**: False
32. **Stakeholder Collaboration**: True
33. **Other Ethical Considerations**: True

Design, Development, and Usability of an Educational AI Chatbot for People with Haemophilia in Senegal [47]

1. **Origin of the Work**: Senegal
2. **Affiliations**: National Blood Transfusion Center, University of Geneva, funded by Novo Nordisk Haemophilia Foundation (NNHF)
3. **Types of Funding**: NGO Funding
4. **Continents of Funders**: Europe
5. **Supported Languages**: French
6. **Target African Countries or Regions**: Senegal
7. **NLP Applications**: Conversational Assistant
8. **Evaluation Method**: User Experience
9. **Technical Evaluation Metrics**: User Engagement, User Satisfaction Score
10. **System Performance**: High user engagement and satisfaction with the chatbot; significant improvement in health literacy among users.
11. **Domain Coverage**: General Domain, Biomedical Domain
12. **Modality**: Text
13. **Primary EPHF**: EPHF 7: Health Promotion
14. **Secondary EPHF**: EPHF 10: Health Service Quality and Equity
15. **Target Users**: Specific equity-seeking groups (people with haemophilia), Researchers and data scientists, Public health officials and policymakers, Healthcare providers
16. **Public Health Challenge**: Enhancing health education and support for people with haemophilia in Senegal to improve disease management and patient outcomes.
17. **Public Health Outcomes**: Significant improvement in health literacy, self-management, and disease understanding among people with haemophilia in Senegal.
18. **Levels of Impact and Engagement**: Direct Impact on Public Health in Africa
19. **Public Health Infrastructure Integration**: True
20. **Interoperability**: True
21. **SDGs**: SDG 3: Good Health and Well-being, SDG 4: Quality Education
22. **SDG 3 Target**: 3.8: Achieve Universal Health Coverage
23. **Year of Publication**: 2022
24. **Year of Data Collection**: 2022
25. **Deployment Stage**: Design and Prototyping
26. **Level of Accessibility**: Limited Access
27. **Available Platform**: Mobile Applications, Web-Based Applications
28. **Ethical Approval**: True
29. **Data Privacy Compliance**: True
30. **Informed Consent**: True
31. **Community Involvement**: True
32. **Stakeholder Collaboration**: True
33. **Other Ethical Considerations**: True

Using Big Data Techniques to Improve Prostate Cancer Reporting in the Gauteng Province, South Africa [48]

1. **Origin of the Work**: South Africa
2. **Affiliations**: University of the Witwatersrand, National Health Laboratory Service
3. **Types of Funding**: Not Mentioned
4. **Continents of Funders**: Not Mentioned
5. **Supported Languages**: English
6. **Target African Countries or Regions**: South Africa
7. **NLP Applications**: Medical Report Generation (Text Mining, Regular Expressions, Bag of Words, N-grams)
8. **Evaluation Method**: Health Research Measure
9. **Technical Evaluation Metrics**: Gleason Score
10. **System Performance**: 286 out of 1000 biopsies reported cell differentiation characteristics. Poorly differentiated adenocarcinoma was noted in 64 biopsies, with 29 reporting moderate differentiation. Gleason scores (GS) were extracted for all 1000 biopsies.
11. **Domain Coverage**: Clinical Domain
12. **Modality**: Text
13. **Primary EPHF**: EPHF 11 (Public Health Research, Evaluation, and Knowledge Management)
14. **Secondary EPHF**: EPHF 10 (Health Service Quality and Equity)
15. **Target Users**: Researchers and Data Scientists, Public Health Officials and Policymakers, Healthcare Providers
16. **Public Health Challenge**: Enhancing the accuracy and comprehensiveness of prostate cancer reporting in Gauteng Province, South Africa.
17. **Public Health Outcomes**: Not Mentioned
18. **Levels of Impact and Engagement**: Systems or Analyses Developed with African Data
19. **Public Health Infrastructure Integration**: Integration with Public Health Systems
20. **Interoperability**: True
21. **SDGs**: SDG 3 (Good Health and Well-being)
22. **SDG 3 Target**: 3.4 (Reduce Premature Mortality from Non-Communicable Diseases)
23. **Year of Publication**: 2019
24. **Year of Data Collection**: 2006-2016
25. **Deployment Stage**: Design and Prototyping
26. **Level of Accessibility**: Publicly Available
27. **Available Platform**: NLP Tools and Libraries
28. **Ethical Approval**: True
29. **Data Privacy Compliance**: True
30. **Informed Consent**: False
31. **Community Involvement**: False
32. **Stakeholder Collaboration**: True
33. **Other Ethical Considerations**: True

Vaginal microbiome topic modeling of laboring Ugandan women with and without fever [49]

1. **Origin of the Work**: Uganda, United States of America, United Kingdom
2. **Affiliations**: Mbale Regional Referral Hospital, Penn State University, Dana Farber Cancer Institute, Boston, MA, USA, CURE Children's Hospital of Uganda, Mbarara University of Science and Technology, University of Liverpool, Liverpool Women's Hospital, Mbale Clinical Research Institute/Busitema University, Massachusetts General Hospital, Boston, MA, USA, Wayne State University, Genentech, Inc.
3. **Types of Funding**: Government Funding
4. **Continents of Funders**: Africa
5. **Supported Languages**: English
6. **Target African Countries or Regions**: Uganda
7. **NLP Applications**: Thematic Analysis
8. **Evaluation Method**: Technical Performance
9. **Technical Evaluation Metrics**: AUC
10. **System Performance**: AUC for predicting maternal febrile status: 0.76
11. **Domain Coverage**: Clinical Domain
12. **Modality**: Text
13. **Primary EPHF**: EPHF 11 (Public Health Research, Evaluation, and Knowledge)
14. **Secondary EPHF**: EPHF 10 (Health Service Quality and Equity)
15. **Target Users**: Specific Equity-Seeking Groups, Researchers and Data Scientists, Public Health Officials and Policymakers, Healthcare Providers
16. **Public Health Challenge**: Understanding the differences in the vaginal microbiome of laboring Ugandan women with and without fever, which can inform better management and treatment of infections during labor.
17. **Public Health Outcomes**: Not Mentioned
18. **Levels of Impact and Engagement**: Systems or Analyses Developed with African Data
19. **Public Health Infrastructure Integration**: True
20. **Interoperability**: True
21. **SDGs**: SDG 3 (Good Health and Well-being)
22. **SDG 3 Target**: 3.1 (Reduce Maternal Mortality)
23. **Year of Publication**: 2021
24. **Year of Data Collection**: Not Mentioned
25. **Deployment Stage**: Design and Prototyping
26. **Level of Accessibility**: Open-Sourced
27. **Available Platform**: NLP Tools and Libraries
28. **Ethical Approval**: True
29. **Data Privacy Compliance**: True
30. **Informed Consent**: True
31. **Community Involvement**: True
32. **Stakeholder Collaboration**: True
33. **Other Ethical Considerations**: True

Chatbot in isiXhosa for Remote Pre/post-Natal Care [50]

1. **Origin of the Work**: South Africa
2. **Affiliations**: Zelda Learning Technologies, University of Cape Town
3. **Types of Funding**: Not Mentioned
4. **Continents of Funders**: Not Mentioned
5. **Supported Languages**: Xhosa
6. **Target African Countries or Regions**: South Africa
7. **NLP Applications**: Conversational Assistant
8. **Evaluation Method**: Technical Performance
9. **Technical Evaluation Metrics**: Accuracy, Phrase Variance
10. **System Performance**: The performance evaluation focuses on accurately understanding specific queries but struggles with high phrase variance, indicating challenges in NLP model robustness.
11. **Domain Coverage**: General Domain, Clinical Domain
12. **Modality**: Text
13. **Primary EPHF**: EPHF 1
14. **Secondary EPHF**: EPHF 7
15. **Target Users**: Healthcare Providers, General Public
16. **Public Health Challenge**: Access to Maternal Health Information in Remote Areas
17. **Public Health Outcomes**: Not Mentioned
18. **Levels of Impact and Engagement**: Systems Developed with African Data
19. **Public Health Infrastructure Integration**: True
20. **Interoperability**: False
21. **SDGs**: SDG 3
22. **SDG 3 Targets**: 3.1, 3.2
23. **Year of Publication**: 2018
24. **Year of Data Collection**: Not Mentioned
25. **Development Stage**: Design and Prototyping
26. **Level of Accessibility**: Limited Access
27. **Available Platform**: Web Services, Mobile Applications
28. **Ethical Approval**: False
29. **Data Privacy Compliance**: False
30. **Informed Consent**: False
31. **Community Involvement**: False
32. **Stakeholder Collaboration**: False
33. **Other Ethical Considerations**: None Mentioned

Expanding Access to Perinatal Depression Treatment in Kenya Through Automated Psychological Support: Development and Usability Study [51]

1. **Origin of the Work**: Kenya, United States of America
2. **Affiliations**: Duke Global Health Institute, United States
   Jacaranda Health, Kenya and United States
   X2AI, United States
   Moi Teaching and Referral Hospital, Kenya
   Africa Mental Health Research and Training Foundation, Kenya
   Duke University, United States
3. **Types of Funding**: Research Grant
4. **Continents of Funders**: North America
5. **Supported Languages**: English
6. **Target African Countries or Regions**: Kenya
7. **NLP Applications**: Automated text messaging, Dialogue system
8. **Evaluation Method**: Technical performance
9. **Technical Evaluation Metrics**: Intervention's effectiveness, User engagement
10. **System Performance**: 7% improvement in mood among participants; positive attitudes towards privacy and service
11. **Domain Coverage**: Clinical domain
12. **Modality**: Text
13. **Primary EPHF**: EPHF 1
14. **Secondary EPHF**: EPHF 7, EPHF 4, EPHF 5
15. **Target Users**: Healthcare providers, Pregnant women, New mothers
16. **Public Health Challenge**: Perinatal depression and lack of access to mental health treatment
17. **Public Health Outcomes**: Effective in improving mood; potential for scaling to benefit a broader population
18. **Levels of Impact and Engagement**: Direct impact on public health in Africa
19. **Public Health Infrastructure Integration**: True
20. **Interoperability**: True
21. **SDGs**: SDG 3, SDG 10
22. **SDG 3 Targets**: 3.1, 3.2
23. **Year of Publication**: 2020
24. **Year of Data Collection**: Not Provided
25. **Development Stage**: Design and prototyping
26. **Level of Accessibility**: Publicly available
27. **Available Platform**: Mobile applications
28. **Ethical Approval**: True
29. **Data Privacy Compliance**: True
30. **Informed Consent**: True
31. **Community Involvement**: True
32. **Stakeholder Collaboration**: True
33. **Other Ethical Considerations**: None Mentioned

Testing interventions to address vaccine hesitancy on Facebook in East and West Africa [52]

1. **Origin of the Work**: United States of America
2. **Affiliations**:
    - University of Chicago Busara Center for Behavioral Economics
3. **Types of Funding**: NGO Funding
4. **Continents of Funders**: North America
5. **Supported Languages**: English
6. **Target African Countries or Regions**: Kenya, Nigeria
7. **NLP Applications**: Conversational assistant (chatbot)
8. **Evaluation Method**: Technical performance, Health research measure, User experience
9. **Technical Evaluation Metrics**: User engagement
10. **System Performance**: The chatbot significantly increased COVID-19 vaccine intentions and willingness by approximately 4-5% compared to the control condition and 3-4% compared to the PSA intervention, indicating a notable positive effect on users' attitudes toward vaccination.
11. **Domain Coverage**: Clinical domain
12. **Modality**: Text
13. **Primary EPHF**: EPHF 1
14. **Secondary EPHF**: EPHF 7 , EPHF 2
15. **Target Users**: General public
16. **Public Health Challenge**: Vaccine hesitancy amid the COVID-19 pandemic
17. **Public Health Outcomes**: Improved vaccine intentions and willingness by addressing individual concerns via a chatbot, with an observed increase in positive vaccine attitudes.
18. **Levels of Impact and Engagement**: Direct impact on public health in Africa
19. **Public Health Infrastructure Integration**: True
20. **Interoperability**: True
21. **SDGs**: SDG 3
22. **SDG 3 Target**: 3.b
23. **Year of Publication**: 2023
24. **Year of Data Collection**: 2022
25. **Deployment Stage**: Deployed and operational
26. **Level of Accessibility**: Publicly available
27. **Available Platform**: Mobile application (Facebook Messenger)
28. **Ethical Approval**: False
29. **Data Privacy Compliance**: True
30. **Informed Consent**: True
31. **Community Involvement**: True
32. **Stakeholder Collaboration**: True
33. **Other Ethical Considerations**: True

Smart-bot Technology: Conversational Agents Role in Maternal Healthcare Support [53]

1. **Origin of the Work**: Kenya
2. **Affiliations**: Maseno University
3. **Types of Funding**: Not Mentioned
4. **Continents of Funders**: Not Mentioned
5. **Supported Languages**: English
6. **Target African Countries or Regions**: Kenya
7. **NLP Applications**: Conversational assistant
8. **Evaluation Method**: Technical performance
9. **Technical Evaluation Metrics**: Accuracy, Precision, Availability, Reinforcement learning, Long-term learning and memory
10. **System Performance**: Not Applicable
11. **Domain Coverage**: Clinical domain
12. **Modality**: Text
13. **Primary EPHF**: EPHF 7
14. **Secondary EPHF**: EPHF 1
15. **Target Users**: Pregnant women in Kenya
16. **Public Health Challenge**: Improving maternal healthcare through better information access and management
17. **Public Health Outcomes**: Improved access to health information which could lead to better health management during pregnancy.
18. **Levels of Impact and Engagement**: Direct impact on public health in Africa
19. **Public Health Infrastructure Integration**: False
20. **Interoperability**: False
21. **SDGs**: SDG 3, SDG 4
22. **SDG 3 Target**: 3.1, 3.2
23. **Year of Publication**: 2019
24. **Year of Data Collection**: Not Mentioned
25. **Deployment Stage**: Design and prototyping
26. **Level of Accessibility**: Publicly available
27. **Available Platform**: Mobile application
28. **Ethical Approval**: False
29. **Data Privacy Compliance**: False
30. **Informed Consent**: False
31. **Community Involvement**: True
32. **Stakeholder Collaboration**: False
33. **Other Ethical Considerations**: None Mentioned

Large Language Models for Sexual, Reproductive and Maternal Health Rights [54]

1. **Origin of the Work**: Ethiopia, Canada, Germany, Hungary
2. **Affiliations**: Debre Markos University, Bahir Dar University, University of Gondar, Ethiopia & Queen's University, Mekdela Amba University, Saint Paul's Hospital Millennium Medical College, Addis Ababa Science and Technology University, HPC & Big Data Analytics CoE, University of Hamburg, University of Miskolc
3. **Types of Funding**: Not Mentioned
4. **Continents of Funders**: Not Mentioned
5. **Supported Languages**: Amharic
6. **Target African Countries or Regions**: Ethiopia
7. **NLP Applications**: Conversational assistant
8. **Evaluation Methods**: Technical performance, User experience
9. **Technical Evaluation Metrics**: BLEU score
10. **System Performance**: BLEU score results: Zero-shot: 0.43, Five-shot: 0.16, Ten-shot: 0.29
11. **Domain Coverage**: Biomedical domain
12. **Modality**: Text
13. **Primary EPHF**: EPHF 7
14. **Secondary EPHF**: EPHF 1
15. **Target Users**: Healthcare providers, General public, Researchers and data scientists
16. **Levels of Impact and Engagement**: Direct impact on public health in Africa
17. **Public Health Challenge**: Sexual and reproductive health challenges, including STIs, unsafe abortions, and maternal health in Ethiopia.
18. **Public Health Outcomes**: Not Mentioned
19. **Public Health Infrastructure Integration**: Community Involvement, Stakeholder Collaboration
20. **Interoperability**: False
21. **SDGs**: SDG 3, SDG 5
22. **SDG 3 Target**: 3.7
23. **Year of Publication**: 2024
24. **Year of Data Collection**: Not Mentioned
25. **Deployment Stage**: Design and prototyping
26. **Level of Accessibility**: Publicly available
27. **Available Platform**: Datasets, NLP tools, and libraries
28. **Ethical Approval**: False
29. **Data Privacy Compliance**: False
30. **Informed Consent**: False
31. **Community Involvement**: True
32. **Stakeholder Collaboration**: True
33. **Other Ethical Considerations**: None Mentioned

9.2 Grey Academic Literature

Auditing Natural Language Processing for Gender Equality in Sub-Saharan African Healthcare Systems: Framework Development and Evaluation [55]

1. **Origin of the Work**: USA, Nigeria
2. **Affiliations**: University of Lagos, Nivi, USAID, Digital Frontiers, Others
3. **Types of Funding**: Government Funding
4. **Continents of Funders**: North America
5. **Supported Languages**: English, Hausa, Swahili
6. **Target African Countries or Regions**: Nigeria, Kenya
7. **NLP Application**: Information Extraction
8. **Evaluation Methods**: Technical Performance, User Experience
9. **Technical Evaluation Metrics**: Precision, Recall
10. **System Performance**:
    - Baseline precision (P) and recall (R): P = 0.725, R = 0.676 for over-represented class; P = 0.669, R = 0.651 for under-represented class.
    - Improved metrics after three iterations: P = 0.721, R = 0.760 for under-represented class.
11. **Domain Coverage**: General Domain
12. **Modality**: Text
13. **Primary EPHF**: EPHF 9
14. **Secondary EPHF**: EPHF 10
15. **Target Users**: Specific Equity-Seeking Groups, Researchers and Data Scientists, Healthcare Providers
16. **Public Health Challenge**: Ensuring gender equality in healthcare systems in Sub-Saharan Africa
17. **Public Health Outcomes**: Not Measured
18. **Levels of Impact and Engagement**: Direct Impact on Public Health in Africa
19. **Public Health Infrastructure Integration**: False
20. **Interoperability**: False
21. **SDGs**: SDG 3, SDG 5
22. **SDG 3 Target**: Sexual and Reproductive Health Services
23. **Year of Publication**: 2023
24. **Year of Data Collection**: Not Mentioned
25. **Deployment Stage**: Design and Prototyping
26. **Level of Accessibility**: Publicly Available
27. **Available Platform**: Mobile Applications, Web-Based Applications
28. **Ethical Approval**: True
29. **Data Privacy Compliance**: True
30. **Informed Consent**: True
31. **Community Involvement**: False
32. **Stakeholder Collaboration**: True
33. **Other Ethical Considerations**: True

Likita: A Medical Chatbot To Improve HealthCare Delivery In Africa [56]

1. **Origin of the Work**: Nigeria, Canada
2. **Affiliations**: Likita Healthcare Ltd, Sterling Bank, Dalhousie University
3. **Types of Funding**: Not Mentioned
4. **Continents of Funders**: Not Mentioned
5. **Supported Languages**: English
6. **Target African Countries or Regions**: Nigeria
7. **NLP Application**: Conversational Assistant
8. **Evaluation Methods**: Technical Performance, User Experience
9. **Technical Evaluation Metrics**: Accuracy, User Satisfaction, Health Outcomes
10. **System Performance**: Iterative improvements, user satisfaction, accessibility, and adoption rates
11. **Domain Coverage**: General Domain
12. **Modality**: Text
13. **Primary EPHF**: EPHF 7
14. **Secondary EPHF**: EPHF 10
15. **Target Users**: Healthcare Providers, General Public
16. **Public Health Challenge**: Improve healthcare delivery in Africa through accessible and reliable medical information and support
17. **Public Health Outcomes**: Not Measured
18. **Levels of Impact and Engagement**: Direct Impact on Public Health in Africa
19. **Public Health Infrastructure Integration**: True
20. **Interoperability**: True
21. **SDGs**: SDG 3, SDG 9
22. **SDG 3 Target**: Universal Health Coverage
23. **Year of Publication**: 2018
24. **Year of Data Collection**: Not Mentioned
25. **Deployment Stage**: Conceptualisation
26. **Level of Accessibility**: Publicly Available
27. **Available Platform**: Mobile Applications, Web-Based Applications
28. **Ethical Approval**: False
29. **Data Privacy Compliance**: False
30. **Informed Consent**: False
31. **Community Involvement**: False
32. **Stakeholder Collaboration**: True
33. **Other Ethical Considerations**: True

Natural Language Generation Requirements for Social Robots in Sub-Saharan Africa [57]

1. **Origin of the Work**: South Africa
2. **Affiliations**: University of Cape Town
3. **Types of Funding**: Research Grant
4. **Continents of Funders**: Africa
5. **Supported Languages**: Zulu, Xhosa, Swati, Ndebele, Northern Sotho, Sesotho, Shona, Venda
6. **Target African Countries or Regions**: South Africa
7. **NLP Applications**: Natural Language Generation, Text-to-Speech, Controlled Natural Languages
8. **Evaluation Methods**: Not Applicable
9. **Technical Evaluation Metrics**: Not Applicable
10. **System Performance**: Not Applicable
11. **Domain Coverage**: General Domain
12. **Modality**: Text
13. **Primary EPHF**: EPHF 10
14. **Secondary EPHF**: EPHF 7
15. **Target Users**: Healthcare Providers, Researchers and Data Scientists, General Public
16. **Public Health Challenge**: Healthcare accessibility and educational challenges in regions with high patient-to-doctor and low teacher-to-student ratios
17. **Public Health Outcomes**: Not Measured
18. **Levels of Impact and Engagement**: Systems or Analyses Developed with African Data
19. **Public Health Infrastructure Integration**: Not Applicable
20. **Interoperability**: Not Applicable
21. **SDGs**: SDG 3, SDG 4
22. **SDG 3 Target**: Universal Health Coverage
23. **Year of Publication**: 2021
24. **Year of Data Collection**: Not Mentioned
25. **Deployment Stage**: Design and Prototyping
26. **Level of Accessibility**: Limited Access
27. **Available Platform**: Not Applicable
28. **Ethical Approval**: True
29. **Data Privacy Compliance**: True
30. **Informed Consent**: False
31. **Community Involvement**: True
32. **Stakeholder Collaboration**: True
33. **Other Ethical Considerations**: True

Applications of Natural Language Processing for Low-Resource Languages in the Healthcare Domain [58]

1. **Origin of the Work**: South Africa
2. **Affiliations**: Stellenbosch University
3. **Types of Funding**: Research Grant, NGO Funding
4. **Continents of Funders**: Africa
5. **Supported Languages**: Afrikaans, English, isiNdebele, isiXhosa, isiZulu, Sesotho, Setswana, siSwati, Tshivenda, Xitsonga
6. **Target African Countries or Regions**: South Africa
7. **NLP Applications**: Question Answering, Information Retrieval, Machine Learning Models, Cross-lingual Embeddings
8. **Evaluation Methods**: Technical Performance
9. **Technical Evaluation Metrics**: Accuracy, Top-1 Test Accuracy, Top-5 Test Accuracy
10. **System Performance**: Transformer models achieved up to 91.16% Top-5 accuracy, demonstrating improved handling of low-resource languages.
11. **Domain Coverage**: Clinical Domain
12. **Modality**: Text
13. **Primary EPHF**: EPHF 10
14. **Secondary EPHF**: EPHF 7
15. **Target Users**: Researchers and Data Scientists, Public Health Officials and Policymakers, Healthcare Providers, General Public
16. **Public Health Challenge**: Enhance healthcare communication and reduce response times for healthcare inquiries
17. **Public Health Outcomes**: Improved response times and accuracy of health information dissemination
18. **Levels of Impact and Engagement**: Integration with Public Health Systems, Community Involvement
19. **Public Health Infrastructure Integration**: True
20. **Interoperability**: Integrated with MomConnect platform (South African National Department of Health), connecting pregnant women to healthcare via SMS and WhatsApp
21. **SDGs**: SDG 3
22. **SDG 3 Target**: 3.1 (Maternal Mortality)
23. **Year of Publication**: 2020
24. **Year of Data Collection**: Not Mentioned
25. **Deployment Stage**: Design and Prototyping
26. **Level of Accessibility**: Limited Access
27. **Available Platform**: Mobile Applications
28. **Ethical Approval**: True
29. **Data Privacy Compliance**: True
30. **Informed Consent**: False
31. **Community Involvement**: True
32. **Stakeholder Collaboration**: True
33. **Other Ethical Considerations**: True

Natural Language Processing to Evaluate Texting Conversations Between Patients and Healthcare Providers During COVID-19 Home-Based Care in Rwanda at Scale [59]

1. **Origin of the Work**: Rwanda, Canada, USA
2. **Affiliations**: University of British Columbia, Rwanda Ministry of Health, Rwanda Biomedical Centre, Luddy School of Informatics, Computing, and Engineering, Indiana University, African Center of Excellence in Data Science, University of Rwanda
3. **Types of Funding**: Research Grant, Government Funding
4. **Continents of Funders**: Africa, North America
5. **Supported Languages**: Kinyarwanda, English
6. **Target African Countries or Regions**: Rwanda
7. **NLP Applications**: Text Classification
8. **Evaluation Methods**: Technical Performance
9. **Technical Evaluation Metrics**: F1 Score
10. **System Performance**:
11. **System Performance**: Random Forest (Diagnostic Methods): F1 = 0.92, Ridge (Prevention): F1 = 0.81, Logistic Regression (Social, Healthcare Logistics, Service Quality, Lifestyle/Behavioral, Technical/IT): F1 = 0.70 - 0.56, BERT (Diagnostic Methods): F1 = 0.96, Longformer (Prevention, Social, Service Quality, Lifestyle/Behavioral): F1 = 0.95 - 0.80, BERT (Technical/IT): F1 = 0.65
12. **Domain Coverage**: Clinical Domain
13. **Modality**: Text
14. **Primary EPHF**: EPHF 1
15. **Target Users**: Public Health Officials and Policymakers, Healthcare Providers
16. **Public Health Challenge**: Management of communicable diseases through remote monitoring
17. **Public Health Outcomes**: Not Measured
18. **Levels of Impact and Engagement**: Direct Impact on Public Health in Africa
19. **Public Health Infrastructure Integration**: Integration with Public Health Systems
20. **SDGs**: SDG 3
21. **SDG 3 Target**: 3.d (Strengthen capacity to handle health risks)
22. **Year of Publication**: 2024
23. **Year of Data Collection**: 2020-2022
24. **Year of Data Analysis**: 2022
25. **Deployment Stage**: Validation
26. **Level of Accessibility**: Publicly Available
27. **Available Platform**: Mobile Applications
28. **Ethical Approval**: True
29. **Data Privacy Compliance**: True
30. **Informed Consent**: False
31. **Community Involvement**: False
32. **Stakeholder Collaboration**: False
33. **Other Ethical Considerations**: None Mentioned

Multi-step Transfer Learning in Natural Language Processing for the Health Domain [60]

1. **Origin of the Work**: South Africa
2. **Affiliations**: Thokozile Manaka, School of Computer Science and Applied Mathematics, University of the Witwatersrand, Johannesburg, Gauteng, South Africa; Terence Van Zyl, Institute for Intelligent Systems, University of Johannesburg, Johannesburg, Gauteng, South Africa; Deepak Kar, School of Physics, University of the Witwatersrand, Johannesburg, Gauteng, South Africa; Alisha Wade, MRC/Wits Rural Public Health and Health Transitions Research Unit, School of Public Health, University of the Witwatersrand, Johannesburg, Gauteng, South Africa
3. **Types of Funding**: Research Grant, NGO Funding
4. **Continents of Funders**: Europe
5. **Supported Languages**: English
6. **Target African Countries or Regions**: South Africa
7. **NLP Applications**: Transfer Learning, Text Classification, Feature Extraction, Fine-tuning
8. **Evaluation Methods**: Technical Performance
9. **Technical Evaluation Metrics**: F1 Score, Accuracy, Precision, Recall
10. **System Performance**:
    - F1 Score = 0.92, Accuracy = 89%, Precision = 91%, Recall = 90%
    - The transfer learning approach significantly outperformed traditional methods, enhancing accuracy in verbal autopsy text classification
11. **Domain Coverage**: Biomedical Domain
12. **Modality**: Text
13. **Primary EPHF**: EPHF 1
14. **Secondary EPHF**: Not Provided
15. **Target Users**: Public Health Officials and Policymakers, Healthcare Providers
16. **Public Health Challenge**: Efficient classification of causes of death in regions with inadequate health documentation systems
17. **Public Health Outcomes**: Direct impact on public health in Africa
18. **Public Health Infrastructure Integration**: Potential for integration with existing health monitoring systems
19. **Interoperability**: False
20. **SDGs**: SDG 3
21. **SDG 3 Target**: 3.d (Strengthen capacity to handle health risks)
22. **Year of Publication**: 2024
23. **Year of Data Collection**: Not Provided
24. **Year of Data Analysis**: Not Provided
25. **Deployment Stage**: Validation
26. **Level of Accessibility**: Closed-Access
27. **Available Platform**: NLP Tools and Libraries
28. **Ethical Approval**: True
29. **Data Privacy Compliance**: True
30. **Informed Consent**: False
31. **Community Involvement**: False
32. **Stakeholder Collaboration**: True
33. **Other Ethical Considerations**: None Mentioned

Systematic Delineation of Media Polarity on COVID-19 Vaccines in Africa: Computational Linguistic Modeling Study [61]

1. **Origin of the Work**: South Africa
2. **Affiliations**:
   - Faculty of Science, University of Johannesburg, Johannesburg
   - Institute for Intelligent Systems, University of Johannesburg, Johannesburg
3. **Types of Funding**: Research Grant
4. **Continents of Funders**: Africa
5. **Supported Languages**: English
6. **Target African Countries or Regions**: Not Specified
7. **NLP Applications**: Sentiment Analysis
8. **Evaluation Method**: Sentiment Polarity Analysis
9. **Technical Evaluation Metrics**: Accuracy
10. **System Performance**:
    - TextBlob: 70% accuracy
    - VADER: 75% accuracy
    - Word2Vec-BiLSTM: 80% accuracy
11. **Domain Coverage**: General Domain
12. **Modality**: Text
13. **Primary EPHF**: EPHF 1
14. **Secondary EPHF**: EPHF 3
15. **Target Users**: Public Health Officials and Policymakers
16. **Public Health Challenge**: Misinformation and varying sentiments regarding COVID-19 vaccines
17. **Public Health Outcomes**: Better informed public health messaging and media strategies
18. **Levels of Impact and Engagement**: Systems or Analyses Developed with African Data
19. **Public Health Infrastructure Integration**: False
20. **Interoperability**: False
21. **SDGs**: SDG 3
22. **SDG 3 Target**: 3.b (Research development and access for vaccines and medicines)
23. **Year of Publication**: 2021
24. **Year of Data Collection**: 2020
25. **Deployment Stage**: Design and Prototyping
26. **Level of Accessibility**: Publicly Available
27. **Available Platform**: NLP Tools and Libraries
28. **Ethical Approval**: True
29. **Data Privacy Compliance**: False
30. **Informed Consent**: False
31. **Community Involvement**: False
32. **Stakeholder Collaboration**: True
33. **Other Ethical Considerations**: None Mentioned

Opinion Mining Analytics for Spotting Omicron Fear-Stimuli Using REPTree Classifier and Natural Language Processing [62]

1. **Origin of the Work**: Nigeria
2. **Affiliations**: Federal College of Education, Abeokuta, Nigeria; Federal University of Agriculture, Abeokuta, Nigeria
3. **Types of Funding**: Not Mentioned
4. **Continents of Funders**: Not Mentioned
5. **Supported Languages**: English
6. **Target African Countries or Regions**: Nigeria
7. **NLP Applications**: Opinion Mining, Sentiment Analysis, REPTree Classifier
8. **Evaluation Method**: Sentiment Analysis
9. **Technical Evaluation Metrics**: Recall = 94.68%, Precision = 94.68%
10. **System Performance**: Identified significant fear stimuli related to hand washing practices among academic staff with 94.68% recall and precision
11. **Domain Coverage**: General Domain
12. **Modality**: Text
13. **Primary EPHF**: EPHF 3
14. **Secondary EPHF**: EPHF 4
15. **Target Users**: Public Health Officials and Policymakers
16. **Public Health Challenge**: Managing public health perceptions and behaviors in response to COVID-19, specifically the Omicron variant
17. **Public Health Outcomes**: Insights to enhance behavioral responses and adherence to health guidelines among academic communities
18. **Levels of Impact and Engagement**: Direct Impact on Public Health in Africa
19. **Public Health Infrastructure Integration**: False
20. **Interoperability**: False
21. **SDGs**: SDG 3
22. **SDG 3 Target**: 3.d (Strengthen Capacity to Handle Health Risks)
23. **Year of Publication**: 2022
24. **Year of Data Collection**: Not Mentioned
25. **Deployment Stage**: Validation
26. **Level of Accessibility**: Publicly Available
27. **Available Platform**: NLP Tools and Libraries
28. **Ethical Approval**: False
29. **Data Privacy Compliance**: False
30. **Informed Consent**: False
31. **Community Involvement**: False
32. **Stakeholder Collaboration**: False
33. **Other Ethical Considerations**: None Mentioned

Natural Language Processing of South African Online Question-Answering Health Forum HIV and AIDS Dataset Using Topic Modeling [63]

1. **Origin of the Work**: South Africa
2. **Affiliations**:
   - Department of Computer System Engineering, Faculty of Information and Communication Technology, Tshwane University of Technology, Soshanguve, South Africa
   - Department of Information Technology, Faculty of Accounting and Informatics, Durban University of Technology, Durban, South Africa
3. **Types of Funding**: Not Mentioned
4. **Continents of Funders**: Not Applicable
5. **Supported Languages**: English
6. **Target African Countries or Regions**: South Africa
7. **NLP Applications**: Topic Modeling, Latent Dirichlet Allocation (LDA)
8. **Evaluation Method**: Topic Modeling
9. **Technical Evaluation Metrics**: Latent Dirichlet Allocation
10. **System Performance**: Identified and categorized topics related to HIV/AIDS from online forums, aiding in understanding public queries and concerns
11. **Domain Coverage**: General Domain
12. **Modality**: Text
13. **Primary EPHF**: EPHF 3
14. **Secondary EPHF**: EPHF 7
15. **Target Users**: Public Health Officials and Policymakers, General Public
16. **Public Health Challenge**: Understanding and responding to public concerns about HIV/AIDS with improved informational support
17. **Public Health Outcomes**: Enhanced public knowledge and response to HIV/AIDS based on insights from community discussions
18. **Levels of Impact and Engagement**: Direct Impact on Public Health in Africa
19. **Public Health Infrastructure Integration**: False
20. **Interoperability**: False
21. **SDGs**: SDG 3
22. **SDG 3 Target**: 3.3 (Communicable diseases, neglected tropical diseases, and water-borne diseases)
23. **Year of Publication**: 2023
24. **Year of Data Collection**: 2018
25. **Deployment Stage**: Design and Prototyping
26. **Level of Accessibility**: Closed-Access
27. **Available Platform**: NLP Tools and Libraries
28. **Ethical Approval**: False
29. **Data Privacy Compliance**: False
30. **Informed Consent**: False
31. **Community Involvement**: False
32. **Stakeholder Collaboration**: False
33. **Other Ethical Considerations**: None Mentioned

A Mixed Cross-Sectional Study with Natural Language Processing Analysis on Computer Literacy and Access Among Healthcare Workers in Guinea [64]

1. **Origin of the Work**: Guinea, USA
2. **Affiliations**:
   - Department of Public Health, Faculty of Health Sciences and Techniques, Gamal Abdel Nasser University, Conakry, Guinea
   - Kofi Annan University of Guinea, Conakry, Guinea
   - National Center for Training and Research in Rural Health of Mafèrinyah, Forécariah, Guinea
   - John Snow, Inc, Boston, Massachusetts, United States
   - University of Central Nicaragua, Managua, Nicaragua
   - Northwestern University, Chicago, United States
3. **Types of Funding**: Not Mentioned
4. **Continents of Funders**: Not Applicable
5. **Supported Languages**: English
6. **Target African Countries or Regions**: Guinea
7. **NLP Applications**: Sentiment Analysis, Word Cloud Analysis
8. **Evaluation Method**: Sentiment Analysis
9. **Technical Evaluation Metrics**: VADER Compound Score
10. **System Performance**: Identified major themes and sentiments related to challenges in computer literacy and access
11. **Domain Coverage**: General Domain
12. **Modality**: Text
13. **Primary EPHF**: EPHF 10
14. **Secondary EPHF**: EPHF 4
15. **Target Users**: Public Health Officials and Policymakers, Healthcare Providers
16. **Public Health Challenge**: Addressing barriers to computer literacy and access to enhance healthcare delivery
17. **Public Health Outcomes**: Findings aim to improve healthcare delivery through better integration of ICT
18. **Levels of Impact and Engagement**: Direct Impact on Public Health in Africa
19. **Public Health Infrastructure Integration**: False
20. **Interoperability**: False
21. **SDGs**: SDG 3
22. **SDG 3 Target**: 3.d (Strengthen Capacity to Handle Health Risks)
23. **Year of Publication**: 2023
24. **Year of Data Collection**: Not Mentioned
25. **Deployment Stage**: Design and Prototyping
26. **Level of Accessibility**: Limited Access
27. **Available Platform**: NLP Tools and Libraries
28. **Ethical Approval**: True
29. **Data Privacy Compliance**: False
30. **Informed Consent**: True
31. **Community Involvement**: False
32. **Stakeholder Collaboration**: True
33. **Other Ethical Considerations**: None Mentioned

AfriSpeech-200: Pan-African Accented Speech Dataset for Clinical and General Domain ASR [65]

1. **Origin of the Work**: Nigeria, Mexico, Canada, South Africa, USA, Germany
2. **Affiliations**: Masakhane NLP, Intron Health, CISPA Helmholtz Center for Information Security, AI Saturdays Lagos, Karya Inc, Mila Quebec AI Institute, Lanfrica, Ford Motor Company, Lelapa AI, McGill University, University of Deusto, Instituto Politecnico Nacional, University of Colorado Colorado Springs, University of Minnesota
3. **Types of Funding**: Not Mentioned
4. **Continents of Funders**: Not Applicable
5. **Supported Languages**: English
6. **Target African Countries or Regions**: Nigeria
7. **NLP Applications**: Speech Recognition, Accented Speech, Clinical ASR
8. **Evaluation Method**: Automatic Speech Recognition
9. **Technical Evaluation Metrics**: Accuracy, Word Error Rate
10. **System Performance**: Improved recognition of diverse African accents in clinical settings, enhancing medical accuracy
11. **Domain Coverage**: Clinical Domain
12. **Modality**: Audio
13. **Primary EPHF**: EPHF 3
14. **Secondary EPHF**: EPHF 10
15. **Target Users**: Healthcare Providers, Researchers, Data Scientists
16. **Public Health Challenge**: Addressing limitations of existing ASR systems in capturing medical discussions with African accents
17. **Public Health Outcomes**: Expected improvements in medical record accuracy and healthcare delivery efficiency
18. **Levels of Impact and Engagement**: Direct Impact on Public Health in Africa
19. **Public Health Infrastructure Integration**: True
20. **Interoperability**: False
21. **SDGs**: SDG 3
22. **SDG 3 Target**: 3.d (Strengthen Capacity to Handle Health Risks)
23. **Year of Publication**: 2023
24. **Year of Data Collection**: Not Mentioned
25. **Deployment Stage**: Design and Prototyping
26. **Level of Accessibility**: Limited Access
27. **Available Platform**: Datasets
28. **Ethical Approval**: True
29. **Data Privacy Compliance**: True
30. **Informed Consent**: False
31. **Community Involvement**: True
32. **Stakeholder Collaboration**: True
33. **Other Ethical Considerations**: True

9.3 Commercial Products and Initiatives

Since commercial products and NLP technologies developed by initiatives typically do not disclose full details regarding their design and implementation, we have indicated "not provided" where this information was unavailable.

World's First AI Health Guidance App in Swahili [66]

1. **Origin of the Work**: Ada's Global Health Initiative (GHI)
2. **Affiliations**: Fondation Botnar,Bill Melinda Gates
3. **Types of Funding**: Not Provided
4. **Continents of Funders**: Not Provided
5. **Supported Languages**: Swahili
6. **Target African Countries or Regions**: Not Provided
7. **NLP Applications**: Not Provided
8. **Evaluation Method**: Not Provided
9. **Technical Evaluation Metrics**: Not Provided
10. **System Performance**: Not Provided
11. **Domain Coverage**: Not Provided
12. **Modality**: Text
13. **Primary EPHF**: Not Provided
14. **Secondary EPHF**: Not Provided
15. **Target Users**: Not Provided
16. **Public Health Challenge**: Not Provided
17. **Public Health Outcomes**: Not Provided
18. **Levels of Impact and Engagement**: Not Provided
19. **Public Health Infrastructure Integration**: Not Provided
20. **Interoperability**: Not Provided
21. **SDGs**: Not Provided
22. **SDG 3 Target**: Not Provided
23. **Year of Publication**: Not Provided
24. **Year of Data Collection**: Not Provided
25. **Deployment Stage**: Not Provided
26. **Level of Accessibility**: Not Provided
27. **Available Platform**: Not Provided
28. **Ethical Approval**: Not Provided
29. **Data Privacy Compliance**: Not Provided
30. **Informed Consent**: Not Provided
31. **Community Involvement**: Not Provided
32. **Stakeholder Collaboration**: Not Provided
33. **Other Ethical Considerations**: Not Provided

Ada Supporting Mothers in South Africa [67]

1. **Origin of the Work**: South Africa
2. **Affiliations**: Ada Health
3. **Types of Funding**: Not Provided
4. **Continents of Funders**: Not Provided
5. **Supported Languages**: Not Provided
6. **Target African Countries or Regions**: Not Provided
7. **NLP Applications**: Not Provided
8. **Evaluation Method**: Not Provided
9. **Technical Evaluation Metrics**: Not Provided
10. **System Performance**: Not Provided
11. **Domain Coverage**: Not Provided
12. **Modality**: Not Provided
13. **Primary EPHF**: Not Provided
14. **Secondary EPHF**: Not Provided
15. **Target Users**: Not Provided
16. **Public Health Challenge**: Not Provided
17. **Public Health Outcomes**: Not Provided
18. **Levels of Impact and Engagement**: Direct Impact on Public Health in Africa
19. **Public Health Infrastructure Integration**: Not Provided
20. **Interoperability**: Not Provided
21. **SDGs**: Not Provided
22. **SDG 3 Target**: Not Provided
23. **Year of Publication**: Not Provided
24. **Year of Data Collection**: Not Provided
25. **Deployment Stage**: Not Provided
26. **Level of Accessibility**: Not Provided
27. **Available Platform**: Not Provided
28. **Ethical Approval**: Not Provided
29. **Data Privacy Compliance**: Not Provided
30. **Informed Consent**: Not Provided
31. **Community Involvement**: Not Provided
32. **Stakeholder Collaboration**: Not Provided
33. **Other Ethical Considerations**: Not Provided

AfriMedQA: The Largest Study on LLMs in African Healthcare [68]

1. **Origin of the Work**: Not Provided
2. **Affiliations**: Google Research, Intron Health, FAMSA Liaison, Georgia Institute of Technology, BioRAMP Clinical, University of Cape Coast, GhanaNLP, Sisonkebiotik
3. **Types of Funding**: NGO Funding, Industry Funding
4. **Continents of Funders**: Not Provided
5. **Supported Languages**: English
6. **Target African Countries or Regions**: Africa
7. **NLP Applications**: Not Provided
8. **Evaluation Method**: Not Provided
9. **Technical Evaluation Metrics**: Not Provided
10. **System Performance**: Not Provided
11. **Domain Coverage**: Not Provided
12. **Modality**: Not Provided
13. **Primary EPHF**: Not Provided
14. **Secondary EPHF**: Not Provided
15. **Target Users**: Not Provided
16. **Public Health Challenge**: Not Provided
17. **Public Health Outcomes**: Not Provided
18. **Levels of Impact and Engagement**: Not Provided
19. **Public Health Infrastructure Integration**: Not Provided
20. **Interoperability**: Not Provided
21. **SDGs**: Not Provided
22. **SDG 3 Target**: Not Provided
23. **Year of Publication**: Not Provided
24. **Year of Data Collection**: Not Provided
25. **Deployment Stage**: Not Provided
26. **Level of Accessibility**: Not Provided
27. **Available Platform**: Not Provided
28. **Ethical Approval**: Not Provided
29. **Data Privacy Compliance**: Not Provided
30. **Informed Consent**: Not Provided
31. **Community Involvement**: Not Provided
32. **Stakeholder Collaboration**: Not Provided
33. **Other Ethical Considerations**: Not Provided

Aurum Institute Ghana Receives Grand Challenges Grant for Catalyzing Equitable Artificial Intelligence (AI) Use [69]

1. **Origin of the Work**: Ghana
2. **Affiliations**: Aurum Institute
3. **Types of Funding**: Bill Melinda Gates Foundation
4. **Continents of Funders**: Not Provided
5. **Supported Languages**: English
6. **Target African Countries or Regions**: Not Provided
7. **NLP Applications**: Not Provided
8. **Evaluation Method**: Not Provided
9. **Technical Evaluation Metrics**: Not Provided
10. **System Performance**: Not Provided
11. **Domain Coverage**: Not Provided
12. **Modality**: Not Provided
13. **Primary EPHF**: Not Provided
14. **Secondary EPHF**: Not Provided
15. **Target Users**: Not Provided
16. **Public Health Challenge**: Not Provided
17. **Public Health Outcomes**: Not Provided
18. **Levels of Impact and Engagement**: Direct Impact on Public Health in Africa
19. **Public Health Infrastructure Integration**: Not Provided
20. **Interoperability**: Not Provided
21. **SDGs**: Not Provided
22. **SDG 3 Target**: Not Provided
23. **Year of Publication**: Not Provided
24. **Year of Data Collection**: Not Provided
25. **Deployment Stage**: Not Provided
26. **Level of Accessibility**: Not Provided
27. **Available Platform**: Not Provided
28. **Ethical Approval**: Not Provided
29. **Data Privacy Compliance**: Not Provided
30. **Informed Consent**: Not Provided
31. **Community Involvement**: Not Provided
32. **Stakeholder Collaboration**: Not Provided
33. **Other Ethical Considerations**: Not Provided

Jacaranda Health [70]

1. **Origin of the Work**: Not Provided
2. **Affiliations**: Jacaranda Health
3. **Types of Funding**: Not Provided
4. **Continents of Funders**: Not Provided
5. **Supported Languages**: Not Provided
6. **Target African Countries or Regions**: Not Provided
7. **NLP Applications**: Conversational Assistant
8. **Evaluation Method**: Not Provided
9. **Technical Evaluation Metrics**: Not Provided
10. **System Performance**: Not Provided
11. **Domain Coverage**: Not Provided
12. **Modality**: Text
13. **Primary EPHF**: Not Provided
14. **Secondary EPHF**: Not Provided
15. **Target Users**: Not Provided
16. **Public Health Challenge**: Not Provided
17. **Public Health Outcomes**: Not Provided
18. **Levels of Impact and Engagement**: Direct Impact on Public Health in Africa
19. **Public Health Infrastructure Integration**: Not Provided
20. **Interoperability**: Not Provided
21. **SDGs**: Not Provided
22. **SDG 3 Target**: Not Provided
23. **Year of Publication**: Not Provided
24. **Year of Data Collection**: Not Provided
25. **Deployment Stage**: Validation
26. **Level of Accessibility**: Not Provided
27. **Available Platform**: Mobile Application
28. **Ethical Approval**: Not Provided
29. **Data Privacy Compliance**: Not Provided
30. **Informed Consent**: Not Provided
31. **Community Involvement**: Not Provided
32. **Stakeholder Collaboration**: Not Provided
33. **Other Ethical Considerations**: Not Provided

Mbaza AI Chatbot [71]

1. **Origin of the Work**: Rwanda
2. **Affiliations**: National Health Authority, the Rwanda Biomedical Center (RBC) and the Rwanda Information Society Authority (RISA), AOS Rwanda, Arxia, Seeing Hands Rwanda, Mozilla Foundation
3. **Types of Funding**: Not Provided
4. **Continents of Funders**: Not Provided
5. **Supported Languages**: Kinyarwanda
6. **Target African Countries or Regions**: Not Provided
7. **NLP Applications**: Conversational Assistant
8. **Evaluation Method**: Not Provided
9. **Technical Evaluation Metrics**: Not Provided
10. **System Performance**: Not Provided
11. **Domain Coverage**: Not Provided
12. **Modality**: Text
13. **Primary EPHF**: Not Provided
14. **Secondary EPHF**: Not Provided
15. **Target Users**: Not Provided
16. **Public Health Challenge**: Not Provided
17. **Public Health Outcomes**: Not Provided
18. **Levels of Impact and Engagement**: Direct Impact on Public Health in Africa
19. **Public Health Infrastructure Integration**: Not Provided
20. **Interoperability**: Not Provided
21. **SDGs**: Not Provided
22. **SDG 3 Target**: Not Provided
23. **Year of Publication**: Not Provided
24. **Year of Data Collection**: Not Provided
25. **Deployment Stage**: Validation
26. **Level of Accessibility**: Not Provided
27. **Available Platform**: Mobile Application
28. **Ethical Approval**: Not Provided
29. **Data Privacy Compliance**: Not Provided
30. **Informed Consent**: Not Provided
31. **Community Involvement**: Not Provided
32. **Stakeholder Collaboration**: Not Provided
33. **Other Ethical Considerations**: Not Provided

Elsa Heath [72]

1. **Origin of the Work**: Tanzania
2. **Affiliations**: Not Provided
3. **Types of Funding**: Not Provided
4. **Continents of Funders**: Not Provided
5. **Supported Languages**: Not Provided
6. **Target African Countries or Regions**: Tanzania
7. **NLP Applications**: Conversational Assistant
8. **Evaluation Method**: Not Provided
9. **Technical Evaluation Metrics**: Not Provided
10. **System Performance**: Not Provided
11. **Domain Coverage**: Not Provided
12. **Modality**: Text
13. **Primary EPHF**: Not Provided
14. **Secondary EPHF**: Not Provided
15. **Target Users**: Not Provided
16. **Public Health Challenge**: Not Provided
17. **Public Health Outcomes**: Not Provided
18. **Levels of Impact and Engagement**: Direct Impact on Public Health in Africa
19. **Public Health Infrastructure Integration**: Not Provided
20. **Interoperability**: Not Provided
21. **SDGs**: Not Provided
22. **SDG 3 Target**: Not Provided
23. **Year of Publication**: Not Provided
24. **Year of Data Collection**: Not Provided
25. **Deployment Stage**: Validation
26. **Level of Accessibility**: Not Provided
27. **Available Platform**: Mobile Application
28. **Ethical Approval**: Not Provided
29. **Data Privacy Compliance**: Not Provided
30. **Informed Consent**: Not Provided
31. **Community Involvement**: Not Provided
32. **Stakeholder Collaboration**: Not Provided
33. **Other Ethical Considerations**: Not Provided

SohphieBot [73]

1. **Origin of the Work**: Kenya
2. **Affiliations**: Not Provided
3. **Types of Funding**: Not Provided
4. **Continents of Funders**: Not Provided
5. **Supported Languages**: English, Swahili
6. **Target African Countries or Regions**: Tanzania
7. **NLP Applications**: Conversational Assistant
8. **Evaluation Method**: Not Provided
9. **Technical Evaluation Metrics**: Not Provided
10. **System Performance**: Not Provided
11. **Domain Coverage**: Not Provided
12. **Modality**: Text
13. **Primary EPHF**: Not Provided
14. **Secondary EPHF**: Not Provided
15. **Target Users**: Not Provided
16. **Public Health Challenge**: Not Provided
17. **Public Health Outcomes**: Not Provided
18. **Levels of Impact and Engagement**: Direct Impact on Public Health in Africa
19. **Public Health Infrastructure Integration**: Not Provided
20. **Interoperability**: Not Provided
21. **SDGs**: SDG3
22. **SDG 3 Target**: Not Provided
23. **Year of Publication**: Not Provided
24. **Year of Data Collection**: Not Provided
25. **Deployment Stage**: Validation
26. **Level of Accessibility**: Not Provided
27. **Available Platform**: Mobile Application
28. **Ethical Approval**: Not Provided
29. **Data Privacy Compliance**: Not Provided
30. **Informed Consent**: Not Provided
31. **Community Involvement**: Not Provided
32. **Stakeholder Collaboration**: Not Provided
33. **Other Ethical Considerations**: Not Provided

References

[1] Maria Ogamba, Joseph Gitonga, Betsy Muriithi, John Olukuru, and Joseph Sevilla. Wellness buddy: An ai mental health chatbot for kenyan university students. In *2023 First International Conference on the Advancements of Artificial Intelligence in African Context (AAIAC)*, pages 1–6. IEEE, 2023.

[2] Elena Arsevska, Mathieu Roche, Sylvain Falala, Renaud Lancelot, David Chavernac, Pascal Hendrikx, and Barbara Dufour. Monitoring disease outbreak events on the web using text-mining approach and domain expert knowledge. In Nicoletta Calzolari, Khalid Choukri, Thierry Declerck, Sara Goggi, Marko Grobelnik, Bente Maegaard, Joseph Mariani, Helene

Mazo, Asuncion Moreno, Jan Odijk, and Stelios Piperidis, editors, *Proceedings of the Tenth International Conference on Language Resources and Evaluation (LREC'16)*, pages 3407–3411, Portorož, Slovenia, May 2016. European Language Resources Association (ELRA).

[3] Antonios Anastasopoulos, Alessandro Cattelan, Zi-Yi Dou, Marcello Federico, Christian Federmann, Dmitriy Genzel, Franscisco Guzmán, Junjie Hu, Macduff Hughes, Philipp Koehn, Rosie Lazar, Will Lewis, Graham Neubig, Mengmeng Niu, Alp Öktem, Eric Paquin, Grace Tang, and Sylwia Tur. TICO-19: the translation initiative for COvid-19. In Karin Verspoor, Kevin Bretonnel Cohen, Michael Conway, Berry de Bruijn, Mark Dredze, Rada Mihalcea, and Byron Wallace, editors, *Proceedings of the 1st Workshop on NLP for COVID-19 (Part 2) at EMNLP 2020*, Online, December 2020. Association for Computational Linguistics.

[4] Jongin Kim, Byeo Rhee Bak, Aditya Agrawal, Jiaxi Wu, Veronika Wirtz, Traci Hong, and Derry Wijaya. COVID-19 vaccine misinformation in middle income countries. In Houda Bouamor, Juan Pino, and Kalika Bali, editors, *Proceedings of the 2023 Conference on Empirical Methods in Natural Language Processing*, pages 3903–3915, Singapore, December 2023. Association for Computational Linguistics.

[5] Abdulmohsen Al-Thubaity, Sakhar Alkhereyf, and Alia O. Bahanshal. AraNPCC: The Arabic newspaper COVID-19 corpus. In Hend Al-Khalifa, Tamer Elsayed, Hamdy Mubarak, Abdulmohsen Al-Thubaity, Walid Magdy, and Kareem Darwish, editors, *Proceedinsg of the 5th Workshop on Open-Source Arabic Corpora and Processing Tools with Shared Tasks on Qur'an QA and Fine-Grained Hate Speech Detection*, pages 32–40, Marseille, France, June 2022. European Language Resources Association.

[6] Ishani Mondal, Kabir Ahuja, Mohit Jain, Jacki O'Neill, Kalika Bali, and Monojit Choudhury. Global readiness of language technology for healthcare: What would it take to combat the next pandemic? In Nicoletta Calzolari, Chu-Ren Huang, Hansaem Kim, James Pustejovsky, Leo Wanner, Key-Sun Choi, Pum-Mo Ryu, Hsin-Hsi Chen, Lucia Donatelli, Heng Ji, Sadao Kurohashi, Patrizia Paggio, Nianwen Xue, Seokhwan Kim, Younggyun Hahm, Zhong He, Tony Kyungil Lee, Enrico Santus, Francis Bond, and Seung-Hoon Na, editors, *Proceedings of the 29th International Conference on Computational Linguistics*, pages 4320–4335, Gyeongju, Republic of Korea, October 2022. International Committee on Computational Linguistics.

[7] Agasthya Gangavarapu. Llms: A promising new tool for improving healthcare in low-resource nations. In *2023 IEEE Global Humanitarian Technology Conference (GHTC)*, pages 252–255, 2023.

[8] Eric P Green, Nicholas Pearson, Sathyanath Rajasekharan, Michiel Rauws, Angela Joerin, Edith Kwobah, Christine Musyimi, Chaya Bhat, Rachel M Jones, and Yihuan Lai. Expanding access to depression treatment in kenya through automated psychological support: Protocol for a single-case experimental design pilot study. *JMIR Res Protoc*, 8(4):e11800, Apr 2019.

[9] Marcel Schmude, Nahya Salim, Hila Azadzoy, Mustafa Bane, Elizabeth Millen, Lisa O'Donnell, Philipp Bode, Ewelina Türk, Ria Vaidya, and Stephen Gilbert. Investigating the potential for clinical decision support in sub-saharan africa with afya (artificial intelligence-based assessment of health symptoms in tanzania): Protocol for a prospective, observational pilot study. *JMIR Res Protoc*, 11(6):e34298, Jun 2022.

[10] Blessing Ogbuokiri, Ali Ahmadi, Nicola Luigi Bragazzi, Zahra Movahedi Nia, Bruce Mellado, Jianhong Wu, James Orbinski, Ali Asgary, and Jude Kong. Public sentiments toward covid-19 vaccines in south african cities: An analysis of twitter posts. *Frontiers in public health*, 10:987376, 2022.

[11] Oladapo Oyebode and Rita Orji. Detecting factors responsible for diabetes prevalence in nigeria using social media and machine learning. In *2019 15th International Conference on Network and Service Management (CNSM)*, pages 1–4, 2019.

[12] David Tresner-Kirsch, Amanda Azari Mikkelson, Chika Yinka-Banjo, Mary Akinyemi, and Siddhartha Goyal. Intent Recognition on Low-Resource Language Messages in a Health Marketplace Chatbot . In *2023 IEEE 11th International Conference on Healthcare Informatics (ICHI)*, pages 457–459, Los Alamitos, CA, USA, June 2023. IEEE Computer Society.

[13] Monica Krishnamoorthy, Mohammed Sharafath Abdul Hameed, Thomas Kopinski, and Andreas Schwung. Disease prediction based on individual's medical history using cnn. In *2021 20th IEEE International Conference on Machine Learning and Applications (ICMLA)*, pages 89–94, 2021.

[14] Richard Ribon Fletcher, Olasubomi Olubeko, Harsh Sonthalia, Fredrick Kateera, Theoneste Nkurunziza, Joanna L. Ashby, Robert Riviello, and Bethany Hedt-Gauthier. Application of machine learning to prediction of surgical site infection. In *2019 41st Annual International Conference of the IEEE Engineering in Medicine and Biology Society (EMBC)*, pages 2234–2237, 2019.

[15] John Batani, Elliot Mbunge, and Lipuo Leokana. A deep learning-based chatbot to enhance maternal health education. In *2024 Conference on Information Communications Technology and Society (ICTAS)*, pages 7–11, 2024.

[16] Blessing Ogbuokiri, Ali Ahmadi, Zahra Movahedi Nia, Bruce Mellado, Jiahong Wu, James Orbinski, Ali Asgary, and Jude Kong. Vaccine hesitancy hotspots in africa: An insight from geotagged twitter posts. *IEEE Transactions on Computational Social Systems*, 11(1):1325–1338, 2024.

[17] Laurette Marais, Johannes A. Louw, Jaco Badenhorst, Karen Calteaux, Ilana Wilken, Nina van Niekerk, and Glenn Stein. Awezamed: A multilingual, multimodal speech-to-speech translation application for maternal health care. In *2020 IEEE 23rd International Conference on Information Fusion (FUSION)*, pages 1–8, 2020.

[18] Navya Annapareddy, Kara Fallin, Ryan Folks, William Jarrard, Marcel Durieux, Nazanin Moradinasab, Bhiken Naik, Saurav Sengupta, Christian Ndaribitse, and Donald Brown. Handwritten text and digit classification on rwandan perioperative flowsheets via yolov5. In *2022 Systems and Information Engineering Design Symposium (SIEDS)*, pages 270–275, 2022.

[19] Mariam Hassib, Nancy Hossam, Jolie Sameh, and Marwan Torki. AraDepSu: Detecting depression and suicidal ideation in Arabic tweets using transformers. In Houda Bouamor, Hend Al-Khalifa, Kareem Darwish, Owen Rambow, Fethi Bougares, Ahmed Abdelali, Nadi Tomeh, Salam Khalifa, and Wajdi Zaghouani, editors, *Proceedings of the Seventh Arabic Natural Language Processing Workshop (WANLP)*, pages 302–311, Abu Dhabi, United Arab Emirates (Hybrid), December 2022. Association for Computational Linguistics.

[20] Ishani Mondal, Kalika Bali, Mohit Jain, Monojit Choudhury, Jacki O'Neill, Millicent Ochieng, Kagnoya Awori, and Keshet Ronen. Language patterns and behaviour of the peer supporters in multilingual healthcare conversational forums. In Nicoletta Calzolari, Frédéric Béchet, Philippe Blache, Khalid Choukri, Christopher Cieri, Thierry Declerck, Sara Goggi, Hitoshi Isahara, Bente Maegaard, Joseph Mariani, Hélène Mazo, Jan Odijk, and Stelios Piperidis, editors, *Proceedings of the Thirteenth Language Resources and Evaluation Conference*, pages 963–975, Marseille, France, June 2022. European Language Resources Association.

[21] Hamdy Mubarak, Sabit Hassan, Shammur Absar Chowdhury, and Firoj Alam. ArCovidVac: Analyzing Arabic tweets about COVID-19 vaccination. In Nicoletta Calzolari, Frédéric Béchet, Philippe Blache, Khalid Choukri, Christopher Cieri, Thierry Declerck, Sara Goggi, Hitoshi Isahara, Bente Maegaard, Joseph Mariani, Hélène Mazo, Jan Odijk, and Stelios Piperidis, editors, *Proceedings of the Thirteenth Language Resources and Evaluation Conference*, pages 3220–3230, Marseille, France, June 2022. European Language Resources Association.

[22] Narshion Ngao, Zeyu Wang, Lawrence Nderu, Tobias Mwalili, Tal August, and Keshet Ronen. Detecting urgency in multilingual medical SMS in Kenya. In Yan Hanqi, Yang Zonghan, Sebastian Ruder, and Wan Xiaojun, editors, *Proceedings of the 2nd Conference of the Asia-Pacific Chapter of the Association for Computational Linguistics and the 12th International Joint Conference on Natural Language Processing: Student Research Workshop*, pages 68–75, Online, November 2022. Association for Computational Linguistics.

[23] Ishani Mondal, Kalika Bali, Mohit Jain, Monojit Choudhury, Ashish Sharma, Evans Gitau, Jacki O'Neill, Kagonya Awori, and Sarah Gitau. A linguistic annotation framework to study interactions in multilingual healthcare conversational forums. In Claire Bonial and Nianwen

Xue, editors, *Proceedings of the Joint 15th Linguistic Annotation Workshop (LAW) and 3rd Designing Meaning Representations (DMR) Workshop*, pages 66–77, Punta Cana, Dominican Republic, November 2021. Association for Computational Linguistics.

[24] Michelle Yuan, Mozhi Zhang, Benjamin Van Durme, Leah Findlater, and Jordan Boyd-Graber. Interactive refinement of cross-lingual word embeddings. In Bonnie Webber, Trevor Cohn, Yulan He, and Yang Liu, editors, *Proceedings of the 2020 Conference on Empirical Methods in Natural Language Processing (EMNLP)*, pages 5984–5996, Online, November 2020. Association for Computational Linguistics.

[25] Ashima Yadav and Dinesh Kumar Vishwakarma. A language-independent network to analyze the impact of covid-19 on the world via sentiment analysis. *ACM Trans. Internet Technol.*, 22(1), September 2021.

[26] Jeanne E. Daniel, Willie Brink, Ryan Eloff, and Charles Copley. Towards automating healthcare question answering in a noisy multilingual low-resource setting. In Anna Korhonen, David Traum, and Lluís Màrquez, editors, *Proceedings of the 57th Annual Meeting of the Association for Computational Linguistics*, pages 948–953, Florence, Italy, July 2019. Association for Computational Linguistics.

[27] Joan Byamugisha, C. Maria Keet, and Brian DeRenzi. Evaluation of a Runyankore grammar engine for healthcare messages. In Jose M. Alonso, Alberto Bugarín, and Ehud Reiter, editors, *Proceedings of the 10th International Conference on Natural Language Generation*, pages 105–113, Santiago de Compostela, Spain, September 2017. Association for Computational Linguistics.

[28] Mampaka Lydia Mojapelo. Semantics of body parts in African WordNet: a case of Northern Sotho. In Christiane Fellbaum, Piek Vossen, Verginica Barbu Mititelu, and Corina Forascu, editors, *Proceedings of the 8th Global WordNet Conference (GWC)*, pages 234–242, Bucharest, Romania, 27–30 January 2016. Global Wordnet Association.

[29] Manah Verma, Nikhil Moudgil, Gaurav Goel, Peehu Pardeshi, Jacquleen Joseph, Neeraj Kumar, Kulbir Singh, Hari Singh, and Prakash Babu Kodali. People's perceptions on covid-19 vaccination: an analysis of twitter discourse from four countries. *Scientific Reports*, 13(1):14281, 2023.

[30] Millie Phiri and Allen Munoriyarwa. Health chatbots in africa: scoping review. *Journal of Medical Internet Research*, 25:e35573, 2023.

[31] Julia L Marcus, Whitney C Sewell, Laura B Balzer, and Douglas S Krakower. Artificial intelligence and machine learning for hiv prevention: emerging approaches to ending the epidemic. *Current HIV/AIDS Reports*, 17:171–179, 2020.

[32] Carlien Vorster, Alta Kritzinger, Lovina E Coetser, and Jeannie Van der Linde. Preliminary reliability of south african adaptation and northern sotho translation of the modified checklist for autism in toddlers, revised with follow-up. *South African Journal of Communication Disorders*, 68(1):1–7, 2021.

[33] Amaury Thiabaud, Isotta Triulzi, Erol Orel, Kali Tal, and Olivia Keiser. Social, behavioral, and cultural factors of hiv in malawi: semi-automated systematic review. *Journal of medical internet research*, 22(8):e18747, 2020.

[34] Abdelghani Ghanem, Chaimae Asaad, Hakim Hafidi, Youness Moukafih, Bassma Guermah, Nada Sbihi, Mehdi Zakroum, Mounir Ghogho, Meriem Dairi, Mariam Cherqaoui, et al. Real-time infoveillance of moroccan social media users' sentiments towards the covid-19 pandemic and its management. *International journal of environmental research and public health*, 18(22):12172, 2021.

[35] Dani Kiyasseh, Tingting Zhu, and David Clifton. The promise of clinical decision support systems targetting low-resource settings. *IEEE Reviews in Biomedical Engineering*, 15:354–371, 2020.

[36] Elaine O Nsoesie, Olubusola Oladeji, Aristide S Abah Abah, and Martial L Ndeffo-Mbah. Forecasting influenza-like illness trends in cameroon using google search data. *Scientific Reports*, 11(1):6713, 2021.

[37] Raghad Alshalan, Hend Al-Khalifa, Duaa Alsaeed, Heyam Al-Baity, and Shahad Alshalan. Detection of hate speech in covid-19–related tweets in the arab region: Deep learning and topic modeling approach. *Journal of Medical Internet Research*, 22(12):e22609, 2020.

[38] Alastair van Heerden, Shannon Bosman, Dallas Swendeman, and Warren Scott Comulada. Chatbots for hiv prevention and care: a narrative review. *Current HIV/AIDS Reports*, 20(6):481–486, 2023.

[39] Naseem Cassim, Michael Mapundu, Victor Olago, Turgay Celik, Jaya Anna George, and Deborah Kim Glencross. Using text mining techniques to extract prostate cancer predictive information (gleason score) from semi-structured narrative laboratory reports in the gauteng province, south africa. *BMC Medical Informatics and Decision Making*, 21:1–11, 2021.

[40] Xolani Ntinga, Franco Musiello, Alfred Kipyegon Keter, Ruanne Barnabas, and Alastair van Heerden. The feasibility and acceptability of an mhealth conversational agent designed to support hiv self-testing in south africa: cross-sectional study. *Journal of Medical Internet Research*, 24(12):e39816, 2022.

[41] Sam Martin, Eliz Kilich, Sara Dada, Per Egil Kummervold, Chermain Denny, Pauline Paterson, and Heidi J Larson. "vaccines for pregnant women...?! absurd"–mapping maternal vaccination discourse and stance on social media over six months. *Vaccine*, 38(42):6627–6637, 2020.

[42] Talita Greyling and Stephanié Rossouw. Positive attitudes towards covid-19 vaccines: A cross-country analysis. *PLoS One*, 17(3):e0264994, 2022.

[43] Jacqueline Du Toit, Retha Hattingh, and Richard Pitcher. The accuracy of radiology speech recognition reports in a multilingual south african teaching hospital. *BMC medical imaging*, 15:1–5, 2015.

[44] Z Movahedi Nia, NL Bragazzi, A Ahamadi, A Asgary, B Mellado, J Orbinski, L Seyyed-Kalantari, WA Woldegerima, J Wu, and JD Kong. Off-label drug use during the covid-19 pandemic in africa: topic modelling and sentiment analysis of ivermectin in south africa and nigeria as a case study. *Journal of the Royal Society Interface*, 20(206):20230200, 2023.

[45] Okechinyere J Achilonu, Elvira Singh, Gideon Nimako, René MJC Eijkemans, and Eustasius Musenge. Rule-based information extraction from free-text pathology reports reveals trends in south african female breast cancer molecular subtypes and ki67 expression. *BioMed Research International*, 2022(1):6157861, 2022.

[46] Zahra Movahedi Nia, Ali Asgary, Nicola Bragazzi, Bruce Mellado, James Orbinski, Jianhong Wu, and Jude Kong. Nowcasting unemployment rate during the covid-19 pandemic using twitter data: the case of south africa. *Frontiers in Public Health*, 10:952363, 2022.

[47] Awa Babington-Ashaye, Philippe de Moerloose, Saliou Diop, and Antoine Geissbuhler. Design, development and usability of an educational ai chatbot for people with haemophilia in senegal. *Haemophilia*, 29(4):1063–1073, 2023.

[48] Naseem Cassim, Michael Mapundu, Victor Olago, Jaya Anna George, and Deborah Kim Glencross. Using big data techniques to improve prostate cancer reporting in the gauteng province, south africa. In *MEDINFO 2019: Health and Wellbeing e-Networks for All*, pages 1437–1438. IOS Press, 2019.

[49] Mercedeh Movassagh, Lisa M Bebell, Kathy Burgoine, Christine Hehnly, Lijun Zhang, Kim Moran, Kathryn Sheldon, Shamim A Sinnar, Edith Mbabazi-Kabachelor, Elias Kumbakumba, et al. Vaginal microbiome topic modeling of laboring ugandan women with and without fever. *npj Biofilms and Microbiomes*, 7(1):75, 2021.

[50] Amit Mishra. Chatbot in isixhosa for remote pre/post-natal care. 2019.

[51] Eric P Green, Yihuan Lai, Nicholas Pearson, Sathyanath Rajasekharan, Michiel Rauws, Angela Joerin, Edith Kwobah, Christine Musyimi, Rachel M Jones, Chaya Bhat, et al. Expanding access to perinatal depression treatment in kenya through automated psychological support: development and usability study. *JMIR Formative Research*, 4(10):e17895, 2020.

[52] Leah R Rosenzweig and Molly Offer-Westort. Testing interventions to address vaccine hesitancy on facebook in east and west africa. *Open Science Framework*, 2022.

[53] Kevin Mugoye, Henry Okoyo, and Sylvester Mcoyowo. Smart-bot technology: Conversational agents role in maternal healthcare support. In *2019 IST-Africa Week Conference (IST-Africa)*, pages 1–7. IEEE, 2019.

[54] Walelign Sewunetie, Assefa Beza, Hailemariam Abebe, Tesfamariam M Abuhay, Wasyihun Admass, Hayat Hassen, Tsion Haile, Hana Hailemariam, Lydia Debebe, Nurlign Moges, et al. Large language models for sexual, reproductive, and maternal health rights. In *2024 IEEE 12th International Conference on Healthcare Informatics (ICHI)*, pages 568–573. IEEE, 2024.

[55] D. Tresner-Kirsch, C. Yinka-Banjo, M. Akinyemi, and O. Ajayi. Auditing natural language processing for gender equality in sub-saharan african healthcare systems: Framework development and evaluation. *JMIR Preprints*, August 2023. Preprint.

[56] Oladapo Oyebode and Rita Orji. Likita: a medical chatbot to improve healthcare delivery in africa. *HCI Across Borders (HCIxB)*, 2018.

[57] C. Maria Keet. Natural language generation requirements for social robots in sub-saharan africa. In *2021 IST-Africa Conference (IST-Africa)*, pages 1–8, 2021.

[58] Jeanne Elizabeth Daniel. *Applications of natural language processing for low-resource languages in the healthcare domain*. PhD thesis, Stellenbosch: Stellenbosch University., 2020.

[59] Richard Todd Lester, Matthew Manson, Muhammed Semakula, Hyeju Jang, Hassan Mugabo, Ali Magzari, Junhong Ma Blackmer, Fanan Fattah, Simon Pierre Niyonsenga, Edson Rwagasore, et al. Natural language processing to evaluate texting conversations between patients and healthcare providers during covid-19 home-based care in rwanda at scale. *medRxiv*, pages 2024–08, 2024.

[60] Thokozile Manaka, Terence Van Zyl, Deepak Kar, and Alisha Wade. Multi-step transfer learning in natural language processing for the health domain. *Neural Processing Letters*, 56(3):177, 2024.

[61] Sefater Gbashi, Oluwafemi Ayodeji Adebo, Wesley Doorsamy, Patrick Berka Njobeh, et al. Systematic delineation of media polarity on covid-19 vaccines in africa: computational linguistic modeling study. *JMIR medical informatics*, 9(3):e22916, 2021.

[62] Taiwo Olapeju Olaleye, Segun Michael Akintunde, Chidi Akparanta, Tijani Agidibo Avovome, OF Oluyen, and AO Akparanta. Opinion mining analytics for spotting omicron fear-stimuli using reptree classifier and natural language processing. *International Journal for Research in Applied Science & Engineering Technology*, pages 995–1005, 2022.

[63] Kyle Stone, Sunday Ojo, and Chunling Tu. Natural language processing of south african online question-answering health forum hiv and aids dataset using topic modeling. In *Intelligent Sustainable Systems: Selected Papers of WorldS4 2022, Volume 2*, pages 387–395. Springer, 2023.

[64] Aboubacar Sidiki Magassouba, Sylla Younoussa, Toure Almamy Amara, Leno Niouma Nestor, Diallo Boubacar Djelo, Camara Gnoume, Nabé Aly Badara, Diallo Mamadou Diao, Afe Abayomi Joseph, and Diallo Ousmane Oumou. A mixed cross-sectional study with natural language processing analysis on computer literacy and access among healthcare workers in guinea. *Journal of Health Informatics in Developing Countries*, 17(02), 2023.

[65] Tobi Olatunji, Tejumade Afonja, Aditya Yadavalli, Chris Chinenye Emezue, Sahib Singh, Bonaventure FP Dossou, Joanne Osuchukwu, Salomey Osei, Atnafu Lambebo Tonja, Naome Etori, et al. Afrispeech-200: Pan-african accented speech dataset for clinical and general domain asr. *Transactions of the Association for Computational Linguistics*, 11:1669–1685, 2023.

[66] Ada Health GmbH. World's first AI health guidance app in swahili, n.d.

[67] Ada Health GmbH. Ada supporting mothers in south africa, 2023.

[68] AfriMedQA. The largest study on llms in african healthcare, n.d.

[69] Aurum Institute. Aurum institute ghana receives grand challenges grant for catalyzing equitable artificial intelligence (AI) use, n.d.

[70] Jacaranda Health. Jacaranda launches open-source LLM in five african languages, n.d.

[71] Digital Umuganda. Mbaza ai chatbot, n.d.

[72] Elsa Health. Dr. elsa, n.d.

[73] Disrupt Africa. Kenya's sophiebot selected for merck nairobi accelerator, September 2017.

A Complete Search Terms for MEDLINE via PubMed

For this scoping review, the search terms applied are a conjunction of three sets of search terms in disjunction forms: **Public Health-Telated terms** AND **NLP-Related Terms** AND **Africa-Related Terms**.

Public Health-Related Terms: "Public Health"[MeSH Terms] OR "Population Health"[MeSH Terms] OR "Global Health"[MeSH Terms] OR "Public Health Surveillance"[MeSH Terms] OR "Environmental Monitoring"[MeSH Terms] OR "Disease Outbreaks"[MeSH Terms] OR "Epidemiologic Methods"[MeSH Terms] OR "Health Status Indicators"[MeSH Terms] OR "Population Surveillance"[MeSH Terms] OR "Biological Monitoring"[MeSH Terms] OR "Contact Tracing"[MeSH Terms] OR "Communicable Diseases"[MeSH Terms] OR "Emergencies"[MeSH Terms] OR "Pandemics"[MeSH Terms] OR "Disaster Planning"[MeSH Terms] OR "Emergency Medical Services"[MeSH Terms] OR "Disaster Medicine"[MeSH Terms] OR "Natural Disasters"[MeSH Terms] OR "Floods"[MeSH Terms] OR "Tsunamis"[MeSH Terms] OR "Earthquakes"[MeSH Terms] OR "Droughts"[MeSH Terms] OR "Famine"[MeSH Terms] OR "Health Policy"[MeSH Terms] OR "Public Health Administration"[MeSH Terms] OR "Health Planning"[MeSH Terms] OR "Legislation, Medical"[MeSH Terms] OR "Legislation as Topic"[MeSH Terms] OR "Population Health Management"[MeSH Terms] OR "Healthcare Financing"[MeSH Terms] OR "Community Health Planning"[MeSH Terms] OR "Healthcare Disparities"[MeSH Terms] OR "Health Inequities"[MeSH Terms] OR "Global Health"[MeSH Terms] OR "Environmental Exposure"[MeSH Terms] OR "Occupational Health"[MeSH Terms] OR "Food Safety"[MeSH Terms] OR "Water Quality"[MeSH Terms] OR "Air Pollution"[MeSH Terms] OR "Water Pollution"[MeSH Terms] OR "Environmental Pollution"[MeSH Terms] OR "Radiation Protection"[MeSH Terms] OR "Risk Assessment"[MeSH Terms] OR "Environmental Indicators"[MeSH Terms] OR "Preventive Health Services"[MeSH Terms] OR "Vaccination"[MeSH Terms] OR "Communicable Disease Control"[MeSH Terms] OR "Noncommunicable Diseases"[MeSH Terms] OR "Primary Prevention"[MeSH Terms] OR "Early Diagnosis"[MeSH Terms] OR "Mass Screening"[MeSH Terms] OR "Primary Health Care"[MeSH Terms] OR "Health Education"[MeSH Terms] OR "Health Behavior"[MeSH Terms] OR "Healthy Lifestyle"[MeSH Terms] OR "Social Determinants of Health"[MeSH Terms] OR "Health Promotion"[MeSH Terms] OR "Health Literacy"[MeSH Terms] OR "Awareness"[MeSH Terms] OR "Community Participation"[MeSH Terms] OR "Social Support"[MeSH Terms] OR "Public Opinion"[MeSH Terms] OR "Community Networks"[MeSH Terms] OR "Community Health Workers"[MeSH Terms] OR "Social Participation"[MeSH Terms] OR "Social Discrimination"[MeSH Terms] OR "Intersectional Framework"[MeSH Terms] OR "Health Workforce"[MeSH Terms] OR "Health Personnel"[MeSH Terms] OR "Education, Medical"[MeSH Terms] OR "Internship and Residency"[MeSH Terms] OR "Community Health Workers"[MeSH Terms] OR "Physicians"[MeSH Terms] OR "Nurses"[MeSH Terms] OR "Volunteers"[MeSH Terms] OR "Midwifery"[MeSH Terms] OR "General Practitioners"[MeSH Terms] OR "Traditional Medicine Practitioners"[MeSH Terms] OR "Medicine, Traditional"[MeSH Terms] OR "Working Conditions"[MeSH Terms] OR "Quality of Health Care"[MeSH Terms] OR "Healthcare Disparities"[MeSH Terms] OR "Patient Safety"[MeSH Terms] OR "Health Equity"[MeSH Terms] OR "Health Inequities"[MeSH Terms] OR "Vulnerable Populations"[MeSH Terms] OR "Health Care Quality, Access, and Evaluation"[MeSH Terms] OR "Health Services Accessibility"[MeSH Terms] OR "Delivery of Health Care"[MeSH Terms] OR

"Biomedical Research"[MeSH Terms] OR "Program Evaluation"[MeSH Terms] OR "Outcome Assessment, Health Care"[MeSH Terms] OR "Evidence-Based Medicine"[MeSH Terms] OR "Health Services Research"[MeSH Terms] OR "Public Health Systems Research"[MeSH Terms] OR "Drug Utilization"[MeSH Terms] OR "Equipment and Supplies"[MeSH Terms] OR "Medical Device Legislation"[MeSH Terms] OR "Telemedicine"[MeSH Terms] OR "Medical Informatics"[MeSH Terms] OR "Digital Health"[MeSH Terms] OR "Equipment and Supplies"[MeSH Terms] OR "Health Resources"[MeSH Terms] OR "Health Care Sector"[MeSH Terms] OR "Pharmaceutical Preparations"[MeSH Terms] OR "supply and distribution"[MeSH Terms] OR "Drugs, Generic"[MeSH Terms] OR "Neglected Diseases"[MeSH Terms] OR "community health" OR "Disease Epidemiology" OR "Outbreak surveillance" OR "Outbreak Monitoring" OR "Infectious disease" OR "disaster response" OR "disaster preparedness" OR "emergency management" OR "emergency preparedness" OR "fast onset climate change" OR "rapid onset climate change" OR "natural hazards" OR "volcano" OR "health governance" OR "health decision making" OR "health laws" OR "health legislation" OR "health management" OR "health budgeting" OR "health financing" OR "occupational safety and health" OR "soil pollution" OR "health protection" OR "environmental quality" OR "air quality" OR "health risk" OR "inoculation" OR "disease prevention" OR "early detection" OR "health screening" OR "family planning" OR "health behaviour change" OR "health behavior change" OR "behavioural health" OR "health messaging" OR "public engagement" OR "community engagement" OR "community health" OR "community mobilisation" OR "healthcare staff" OR "doctors" OR "public health officials" OR "public health workers" OR "clinicians" OR "female health workers" OR "traditional healer" OR "herbalist" OR "village healer" OR "healer" OR "continued professional development" OR "labor shortages" OR "labour shortages" OR "health outcomes" OR "healthcare quality" OR "health disparities" OR "healthcare access" OR "access to health" OR "healthcare availability" OR "health systems" OR "health research" OR "intervention evaluation" OR "health evidence" OR "essential medicines" OR "health supply chain" OR "health equipment" OR "health supplies" OR "health products" OR "Pharmaceuticals" OR "neglected tropical diseases" OR "NTD"

NLP-Related Terms: "Natural Language Processing"[MeSH Terms] OR "Data Mining"[MeSH Terms] OR "Sentiment Analysis"[MeSH Terms] OR "Expert Systems"[MeSH Terms] OR "Voice Recognition"[MeSH Terms] OR "Speech Recognition Software"[MeSH Terms] OR "Answering Services"[MeSH Terms] OR "language processing" OR "NLP" OR "computational linguistics" OR "chat" OR "language models" OR "chatgpt" OR "LLM" OR "LLMs" OR "language technology" OR "language technologies" OR "text mining" OR "text analysis" OR "sentiment analysis" OR "information extraction" OR "topic modeling" OR "keyword extraction" OR "opinion mining" OR "emotion detection" OR "affective computing" OR "clinical entity recognition" OR "name extraction" OR "medication extraction" OR "symptom extraction" OR "voice recognition" OR "named entity recognition" or "NER" OR "automated transcription" OR "machine translation" OR "information retrieval" OR "chatbot*" OR "virtual assistant*" OR "dialogue system*" OR "natural language understanding" OR "NLU" OR "NLG" OR "natural language generation" OR "misinformation detection"

Africa-Related Terms: "ethiopia"[MeSH Terms] OR "ethiopia"[All Fields] OR "gabon"[MeSH Terms] OR "gabon"[All Fields] OR "gambia"[MeSH Terms] OR "gambia"[All Fields] OR "ghana"[MeSH Terms] OR "ghana"[All Fields] OR "guinea"[MeSH Terms] OR "guinea"[All Fields] OR "equatorial guinea"[All Fields] OR "guinea-bissau"[MeSH Terms] OR "guinea-bissau"[All Fields] OR "kenya"[MeSH Terms] OR "kenya"[All Fields] OR "lesotho"[MeSH Terms] OR "lesotho"[All Fields] OR "liberia"[MeSH Terms] OR "liberia"[All Fields] OR "libya"[MeSH Terms] OR "libya"[All Fields] OR "madagascar"[MeSH Terms] OR "madagascar"[All Fields] OR "malawi"[MeSH Terms] OR "malawi"[All Fields] OR "mali"[MeSH Terms] OR "mali"[All Fields] OR "mauritania"[MeSH Terms] OR "mauritania"[All Fields] OR "mauritius"[MeSH Terms] OR "mauritius"[All Fields] OR "morocco"[MeSH Terms] OR "morocco"[All Fields] OR "mozambique"[MeSH Terms] OR "mozambique"[All Fields] OR "namibia"[MeSH Terms] OR "namibia"[All Fields] OR "niger"[MeSH Terms] OR "niger"[All Fields] OR "nigeria"[MeSH Terms] OR "nigeria"[All Fields] OR "rwanda"[MeSH Terms] OR "rwanda"[All Fields] OR "sao tome and principe"[MeSH Terms] OR "sao tome and principe"[All Fields] OR "senegal"[MeSH Terms] OR "senegal"[All Fields] OR "seychelles"[MeSH Terms] OR "seychelles"[All Fields] OR "sierra leone"[MeSH Terms] OR "sierra leone"[All Fields] OR "somalia"[MeSH Terms] OR "somalia"[All Fields] OR "south africa"[MeSH Terms] OR "south africa"[All Fields] OR "south sudan"[MeSH Terms] OR "south sudan"[All Fields] OR "sudan"[MeSH Terms] OR "sudan"[All Fields] OR "tanzania"[MeSH Terms] OR "tanzania"[All Fields] OR "togo"[MeSH Terms] OR "togo"[All Fields]

OR “tunisia”[MeSH Terms] OR “tunisia”[All Fields] OR “uganda”[MeSH Terms] OR “uganda”[All Fields] OR “zambia”[MeSH Terms] OR “zambia”[All Fields] OR “zimbabwe”[MeSH Terms] OR “zimbabwe”[All Fields] OR “africa”[MeSH Terms] OR “africa”[All Fields] OR “south africa”[MeSH Terms] OR (“south”[All Fields] AND “africa”[All Fields]) OR “south africa”[All Fields] OR “cote d’ivoire”[MeSH Terms] OR (“cote”[All Fields] AND “d’ivoire”[All Fields]) OR “cote d’ivoire”[All Fields] OR (“ivory”[All Fields] AND “coast”[All Fields]) OR “ivory coast”[All Fields] OR eswatini[All Fields] OR western[All Fields] AND (“africa, northern”[MeSH Terms] OR (“africa”[All Fields] AND “northern”[All Fields]) OR “northern africa”[All Fields] OR “sahara”[All Fields]) AND (“arabs”[MeSH Terms] OR “arabs”[All Fields] OR “arab”[All Fields]) OR “swaziland”[All Fields] OR “algeria”[MeSH Terms] OR “algeria”[All Fields] OR “benin”[MeSH Terms] OR “benin”[All Fields] OR (“benin”[All Fields] AND “republic”[All Fields]) OR “benin republic”[All Fields] OR “benin”[MeSH Terms] OR “benin”[All Fields] OR “togo”[MeSH Terms] OR “togo”[All Fields] OR “angola”[MeSH Terms] OR “angola”[All Fields] OR “botswana”[MeSH Terms] OR “botswana”[All Fields] OR “burkina faso”[MeSH Terms] OR (“burkina”[All Fields] AND “faso”[All Fields]) OR “burkina faso”[All Fields] OR “eritrea”[MeSH Terms] OR “eritrea”[All Fields] OR sahrawi[All Fields] OR “burundi”[MeSH Terms] OR “burundi”[All Fields] OR “egypt”[MeSH Terms] OR “egypt”[All Fields] OR “djibouti”[MeSH Terms] OR “djibouti”[All Fields] OR “democratic republic of the congo”[MeSH Terms] OR (“democratic”[All Fields] AND “republic”[All Fields] AND “congo”[All Fields]) OR “democratic republic of the congo”[All Fields] OR “comoros”[MeSH Terms] OR “comoros”[All Fields] OR “cabo verde”[MeSH Terms] OR (“cabo”[All Fields] AND “verde”[All Fields]) OR “cabo verde”[All Fields] OR (“cape”[All Fields] AND “verde”[All Fields]) OR “cape verde”[All Fields] OR “cameroon”[MeSH Terms] OR “cameroon”[All Fields] OR “central african republic”[MeSH Terms] OR (“central”[All Fields] AND “african”[All Fields] AND “republic”[All Fields]) OR “central african republic”[All Fields] OR “chad”[MeSH Terms] OR “chad”[All Fields] OR “arabic” OR “pidgin” OR “hausa” OR “swahili” OR “amharic” OR “yoruba” OR “oromo” OR “lingala” OR “igbo” OR “zulu” OR “somali” OR “wolof” OR “xhosa” OR “afrikaans” OR “fulfulde” OR “kinyarwanda” OR “chichewa” OR “bamanankan” OR “akan” OR “setswana” OR “sotho” OR “rundi” OR “jula” OR “kituba” OR “moore” OR “ganda” OR “shona” OR “ibibio” OR “tsonga” OR “tigrigna” OR “kanuri” OR “gikuyu” OR “sukuma” OR “kongo” OR “kabyle” OR “krio” OR “malagasy” OR “lubakasai” OR “pulaar” OR “zarma” OR “kimbundu” OR “tachelhit” OR “dagbani” OR “éwé” OR “baoulé” OR “kamba” OR “dholuo” OR “sango” OR “sidamo” OR “pular” OR “swati” OR “tiv” OR “bemba” OR “lomwe” OR “yao” OR “bangala” OR “tamazight” OR “ndebele” OR “venda” OR “soga” OR “kikongo” OR “ateso” OR “maay” OR “chokwe” OR “afar” OR “efik” OR “mende” OR “susu” OR “izon” OR “sénoufo” OR “chiga” OR “tumbuka” OR “soninke” OR “fon” OR “themne” OR “ebira” OR “lango” OR “mandinka” OR “edo” OR “sena” OR “makonde” OR “hadiyya” OR “kimîîru” OR “tarifit” OR “lugbara” OR “haya” OR “kipsigis” OR “zande” OR “maasai” OR “alur” OR “anyin” OR “nuer” OR “bulu” OR “gamo” OR “igala” OR “acholi” OR “nsenga” OR “dan” OR “gun” OR “tonga” OR “ndau” OR “gourmanchéma” OR “gedeo” OR “oshiwambo” OR “abron” OR “silte” OR “dinka” OR “berom” OR “tamajaq” OR “kafa” OR “fur” OR “gbagyi” OR “aja” OR “ha” OR “bukusu” OR “hehe” OR “kigiryama” OR “nyaneka” OR “urhobo” OR “chopi” OR “fang” OR “gogo” OR “nyemba” OR “kibala” OR “turkana” OR “tswa” OR “dangme” OR “bisa” OR “songe” OR “afri*” OR “afro*”
